# Coordination engineering of iridium nanocluster bifunctional electrocatalyst for highly efficient and pH-universal overall water splitting

**DOI:** 10.1038/s41467-020-18064-w

**Published:** 2020-08-25

**Authors:** Qilun Wang, Cong-Qiao Xu, Wei Liu, Sung-Fu Hung, Hong Bin Yang, Jiajian Gao, Weizheng Cai, Hao Ming Chen, Jun Li, Bin Liu

**Affiliations:** 1 School of Chemical and Biomedical Engineering, Nanyang Technological University, Singapore, 637459 Singapore; 2 Department of Chemistry, Southern University of Science and Technology, Shenzhen, 518055 China; 3 Dalian Institute of Chemical Physics, Chinese Academy of Sciences, Dalian, 116023 China; 4 Department of Chemistry, National Taiwan University, Taipei, 10617 Taiwan; 5 Institute for Materials Science and Devices, Suzhou University of Science and Technology, Suzhou, 215009 China; 6 Department of Chemistry and Key Laboratory of Organic Optoelectronics and Molecular Engineering of Ministry of Education, Tsinghua University, Beijing, 100084 China

**Keywords:** Energy, Electrocatalysis, Nanoscale materials

## Abstract

Water electrolysis offers a promising energy conversion and storage technology for mitigating the global energy and environmental crisis, but there still lack highly efficient and pH-universal electrocatalysts to boost the sluggish kinetics for both cathodic hydrogen evolution reaction (HER) and anodic oxygen evolution reaction (OER). Herein, we report uniformly dispersed iridium nanoclusters embedded on nitrogen and sulfur co-doped graphene as an efficient and robust electrocatalyst for both HER and OER at all pH conditions, reaching a current density of 10 mA cm^−2^ with only 300, 190 and 220 mV overpotential for overall water splitting in neutral, acidic and alkaline electrolyte, respectively. Based on probing experiments, operando X-ray absorption spectroscopy and theoretical calculations, we attribute the high catalytic activities to the optimum bindings to hydrogen (for HER) and oxygenated intermediate species (for OER) derived from the tunable and favorable electronic state of the iridium sites coordinated with both nitrogen and sulfur.

## Abstract (web summary)

Water electrolysis offers a promising energy conversion technology, although there is still a need to understand the catalysis on the atomic-level. Here, the authors report Ir nanoclusters coordinated with both N and S as an efficient and pH-universal electrocatalyst for overall water splitting.

## Introduction

Electrochemical water splitting to hydrogen powered by renewable electricity offers a promising strategy to develop a global-scale, sustainable, and fossil-free energy system^1–3^. However, the scalable industrial application of water splitting is still hampered by the huge energy penalty resulting from the sluggish kinetics of the two water electrolysis half-reactions, i.e., the cathodic hydrogen evolution reaction (HER) and anodic oxygen evolution reaction (OER)^4,5^. Thus, developing highly efficient bifunctional electrocatalysts for both HER and OER in the same electrolyte with low overpotentials to drive catalysis is of great significance owing to their integrated merits in simplifying the device fabrication and reducing the cost^6–8^. Moreover, considering the inevitable variation of proton concentration during the water electrolysis process, a desirable electrocatalyst is also required to function well in a wide range of pH conditions so that the operation can be more reliable and energy-efficient^9–11^. Unfortunately, most of the current intriguing electrocatalysts such as layer double hydroxides (LDHs) (e.g., NiFe LDHs)^12^, transition-metal oxides, (oxy)hydroxides (e.g., FeOOH)^13^, and phosphides (e.g., Rh_2_P, PdP_2_)^14,15^ have specific limitations and cannot satisfy all requirements as stated above. Therefore, it is appealing to rationally design cost-effective, highly efficient, and pH-universal bifunctional electrocatalyst for overall water splitting.

By comparing the experimentally measured and theoretically calculated volcano relationships for HER^16^ and OER^17^, we find that iridium (Ir) can be a promising candidate as a bifunctional electrocatalyst for overall water splitting because it sits very near the vertex of both volcanoes (Sabatier principle). Although substantial progresses have been achieved over the past decade, fabrication of an Ir catalyst with adequate mass activity and stability is still a long-term goal, impeded by the elusive nature of active sites and the adverse reconstruction under working conditions^18,19^. To overcome these obstacles, we propose to fix Ir by non-metal element(s) to improve the catalytic stability and at the same time engineer the coordination environment of Ir to tune the adsorption energy of reaction intermediates for promoting intrinsic catalytic activity. Unfortunately, direct fingerprint of intermediates adsorption energy as well as insights into the coordination and electronic structure evolutions under reaction conditions are rarely available, thus an overall comprehension of the active sites corresponded with related adsorbate binding energy by probing experiments and in situ measurements under operating conditions is urgently needed to understand and further design next-generation efficient electrocatalysts^20^.

Herein, we report an efficient and durable bifunctional electrocatalyst of uniformly dispersed, ultrafine, and N,S-coordinated Ir nanoclusters embedded on N,S-doped graphene (denoted as Ir-NSG) for both HER and OER. The as-synthesized Ir-NSG catalyst exhibits superior activities to most of the reported state-of-the-art HER and OER catalysts at all pH conditions, as well as exceptional mass activities outperforming the benchmark commercial Pt/C and Ir/C catalysts. The results of underpotentially deposited hydrogen (H_upd_) and methanol oxidation experiments unravel that the high intrinsic catalytic activities originate from the optimized hydrogen and oxygen intermediates binding energies, which can accelerate both kinetics of HER and OER. At the atomic level, density functional theory (DFT) and operando X-ray absorption fine structure (XAFS) spectroscopy investigations validate that such optimum binding energies are induced by the unique electronic state and coordination environment of Ir sites bonded with both N and S. Interestingly, the Ir-NSG catalyst displays superb performance when integrated directly as both the anode and cathode electrodes in a water electrolysis cell at all pH values, reaching a geometric current density of 10 mA cm^−2^ with as low as 300, 190, and 220 mV overpotential for overall water splitting in neutral, acidic, and alkaline electrolyte, respectively, serving as a promising candidate for next-generation water-splitting technologies^21^.

## Results

### Synthesis and structural characterization of Ir-NSG catalyst

**Fig. 1:**
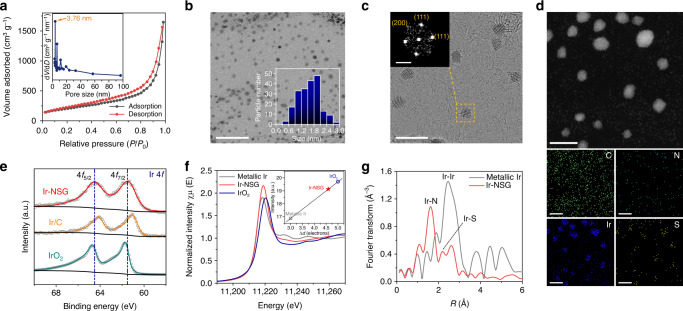
Structural characterization of Ir nanoclusters dispersed on N,S-doped graphene. **a** Nitrogen adsorption isotherm of Ir-NSG measured at 77 K. The inset shows the corresponding BJH pore size distribution curve. **b** Bright-field TEM image (inset shows the corresponding particle size distribution of Ir nanoclusters based on a count of 200 in the sample area), scale bar: 20 nm. **c** Bright-field HRSTEM image and the corresponding FFT of the selected area in the yellow square (inset), scale bars: 5 nm and 5 nm^−1^ for the TEM and FFT pattern, respectively. **d** Dark-field STEM image and EDX elemental mappings, scale bars: 5 nm. **e** High-resolution XPS Ir 4 *f* spectra. **f** Ir L_3_-edge XANES spectrum of Ir-NSG with reference metallic Ir and IrO_2_ (inset shows the integrated while line (WL) intensity as a function of 5*d*-band holes (Δ*d*), which is calibrated from metallic Ir (5*d*^7^) and IrO_2_ (5*d*^5^) standards). **g** Fourier transformation of the EXAFS spectra of Ir-NSG and metallic Ir.

The Ir nanoclusters embedded on N,S-doped graphene catalyst was synthesized via pyrolyzing a homogenous mixture of melamine, amino acid (l-cysteine) and Ir precursor (iridium(III) chloride) in an argon atmosphere. The obtained Ir-NSG has a three-dimensional (3D), mesoporous, and entangled sheet-like structure, which can be clearly observed in field-emission scanning electron microscope (FESEM) (Supplementary Figs. 1 and 2). The measured specific Brunauer-Emmett-Teller (BET) surface area is ~730 m^2^ g^−1^ (Fig. 1a), which is significantly higher than that of the commercial Ir/C catalyst (~170 m^2^ g^−1^) (Supplementary Fig. 3). The corresponding pore size distribution derived from Barrett-Joyner-Halenda (BJH) method shows the presence of mesopores with a pore volume of 2.546 cm^3^ g^−1^ and an average pore diameter of 3.76 nm (inset of Fig. 1a). The unique morphology of Ir-NSG was further examined by transmission electron microscopy (TEM). As revealed in the bright-field TEM images, the Ir nanoclusters are found uniformly distributed within the graphene matrix (Supplementary Fig. 4 and Fig. 1b), which have an average diameter of ~1.55 nm with a narrow size distribution (inset in Fig. 1b). Furthermore, it is interesting to note that the Ir nanoclusters are not encapsulated by a shell of graphitic carbon as commonly observed in graphene-supported transition-metal catalysts^22^, but are intimately embedded in the graphene matrix to ensure their direct exposure to the external surrounding^23^. The bright-field high-resolution scanning TEM (HRSTEM) images, combined with the corresponding fast-Fourier transform (FFT) pattern clearly show the nanoscale crystalline structure of the Ir nanoclusters (Fig. 1c and Supplementary Fig. 5). The interplanar crystal lattice spacings are measured at 0.222 and 0.192 nm, which correspond to the (111) and (200) planes of the face-centered cubic (FCC) Ir, respectively^24^. The apparent clustering of Ir and the homogeneous spatial distribution of Ir nanoclusters in N,S-doped graphene framework were confirmed by STEM coupled with energy-dispersive X-ray spectroscopy (EDX) elemental mappings and line scans (Fig. 1d and Supplementary Fig. 6). It is noticeable that the intensities of N and S are proportional to the content of Ir along the edge but negligible in the bulk of the nanocluster, suggesting that the N and S may immobilize Ir nanoclusters with a core-shell structure by formation of Ir-N and/or Ir-S bonds at the shell region, whereas Ir-Ir bond in the core^25–27^. Electron energy-loss spectroscopy (EELS) measurements were performed to gain more information about the electronic structure of Ir-NSG. As shown in Supplementary Fig. 7, the peaks at ~54.0–60.0 eV can be ascribed to Ir O, whereas the peaks at 63.8 and 66.8 eV are for the Ir N_7_ and N_6_ transitions, respectively, implying an oxidized state of Ir^28^.

Supplementary Fig. 8 displays the powder X-ray diffraction (XRD) pattern of Ir-NSG. The relatively weaker and broader peak at ~26.2° (belonging to graphite-like carbon (002) plane (JCPDS no. 75-1621)) for Ir-NSG as compared with that for commercial Ir/C suggests that doping N and S results in a lower degree of graphitic crystallinity and defect-rich structure for the graphene substrate, which is further confirmed by broader D and G bands at ca. 1330 and 1570 cm^−1^ as well as a higher corresponding intensity ratio *I*_D_/*I*_G_ of the Raman spectrum than those for commercial Ir/C (Supplementary Fig. 9). The wide-angle XRD pattern also shows that the Ir nanoclusters have a metallic FCC structure (JCPDS no. 06-0598), and the peaks at 40.7° and 47.3° can be well indexed to the (111) and (200) planes of Ir, respectively. All of the structural insights obtained from XRD and Raman measurements are consistent with the results of SEM, BET, and TEM. The chemical composition and valence states at the surface of Ir-NSG were further examined by X-ray photoelectron spectroscopy (XPS) (Supplementary Fig. 10). The mass content of Ir in Ir-NSG determined by XPS is ∼8.34 wt.% and close to that measured by inductively coupled plasma atomic emission spectroscopy (ICP-AES) (7.33 wt.%), which is the optimized sample on the basis of Ir loading amount (Supplementary Figs. 11 and 12). The high-resolution XPS N 1*s* spectrum could be deconvoluted into Ir-N (~398.2 eV), pyridinic (~398.3 eV), pyrrolic (~399.6 eV), graphitic (~400.9 eV) and oxidized (~402.3 eV) N species, respectively (Supplementary Fig. 10c), and the N of Ir-N is in the pyridinic form^25,29,30^. Intriguingly, negative shift is observed for S 2*p*_3/2_ (163.9 eV) and S 2*p*_1/2_ (165.0 eV) in the corresponding high-resolution XPS S 2*p* spectrum (Supplementary Fig. 10d), implying that S might be immobilized through bonding in -C-S- and Ir-S in consideration of its larger electronegativity than those of C and Ir^27^. The core level Ir 4 *f* spectra of Ir-NSG, iridium(IV) oxide (IrO_2_) and commercial Ir/C are recorded in Fig. 1e, it should be noted that the full widths at half maximum of Ir 4*f*_5/2_ and Ir 4*f*_7/2_ for Ir-NSG are obviously broader than those for IrO_2_ and Ir/C, suggesting coexistence of various valence states due to the different electronegativities of N and S atoms^31^. Moreover, the corresponding binding energy of Ir for Ir-NSG is lower than that for IrO_2_ but higher than that for Ir/C (approximately dominated by the peaks of metallic Ir^0^) as highlighted by the dash line, indicating partially oxidized Ir sites in the shell of Ir-NSG.

To further elucidate the local electronic and coordination structure of the as-obtained Ir-NSG, we conducted XAFS measurements in which reference spectra from IrO_2_ (5*d*^5^6*s*^0^) and metallic Ir (5*d*^7^6*s*^2^) were collected to fingerprint the features. The three X-ray absorption near edge structure (XANES) spectra at the Ir L_3_-edge (Fig. 1f) show similar characteristic features except for the peak positions and relative intensities. The prominent peak of Ir L_3_-edge, which is historically called the white line (WL), corresponds to the electron transition from the occupied 2*p*_3/2_ orbital to the partially occupied Ir 5*d* orbitals and the magnitude of its integrated intensity is directly proportional to the density of unoccupied 5*d* orbitals (Supplementary Fig. 13)^32,33^. The inset in Fig. 1f shows the relationship of corresponding integrated intensities for the three samples indicated, it is obvious that the WL intensity of the Ir L_3_-edge for Ir-NSG is considerably higher than that for metallic Ir but lower than that for IrO_2_, revealing that the number of unoccupied states in the 5*d* band for Ir-NSG is between those of IrO_2_ and metallic Ir, which has been utilized to correlate the catalytic activities of noble metal-based electrocatalysts to changes in their local electronic states^33,34^. The Fourier transforms of the phase-uncorrected extended X-ray absorption fine structure (EXAFS) spectra are plotted in Supplementary Fig. 14 and Fig. 1g to probe the local environment of Ir. Peaks at ~2.5 Å, which are associated with the Ir–Ir interaction, appear in both Ir-NSG and metallic Ir. For Ir-NSG, the main peak at ~1.65 Å corresponds to the scattering interaction between Ir and N, and an additional small peak appearing at ~2.2 Å can be ascribed to Ir-S bond, which is consistent with the fact that the length of metal-S bond is longer than metal-N bond as previously reported^35^. Combining the above results, the vacancy of electrons generated by the formation of 5*d* holes as well as Ir-N and Ir-S coordination around the Ir shell sites can be identified in Ir-NSG.

### HER and OER performances of Ir-NSG catalyst

**Fig. 2:**
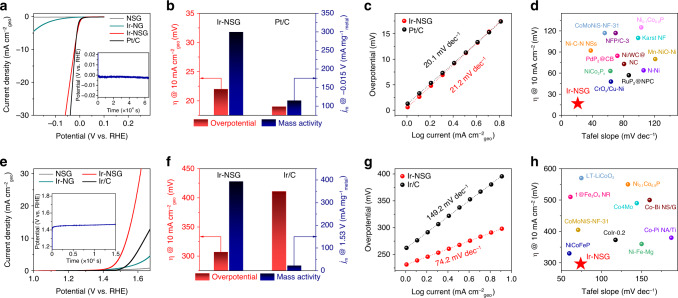
HER and OER performance of the Ir-NSG catalyst in neutral electrolyte. **a** HER polarization curves of Ir-NSG and Pt/C acquired at a sweep rate of 1 mV s^−1^ in Ar-saturated 1 M PBS. Inset shows the chronopotentiometry of Ir-NSG. **b** Comparison of overpotential (*η*) to drive a current density of 10 mA cm^−2^ and metal-mass specific activity (*j*_m_) at −0.015 V (vs. RHE) between Ir-NSG and Pt/C. **c** The corresponding Tafel plots for Ir-NSG and Pt/C. **d** Comparison of overpotential (@ 10 mA cm^−2^) and Tafel slope for various state-of-the-art HER catalysts in neutral medium. **e** Polarization curves of Ir-NSG and Ir/C acquired at a sweep rate of 1 mV s^−1^ in 1 M PBS. Inset shows the chronopotentiometry of Ir-NSG. **f** Comparison of overpotential to drive a current density of 10 mA cm^−2^ and metal-mass specific activity at 1.53 V (vs. RHE) between Ir-NSG and Ir/C. **g** The corresponding Tafel plots for Ir-NSG and Ir/C. **h** Comparison of overpotential (@ 10 mA cm^−2^) and Tafel slope for various state-of-the-art OER catalysts in neutral medium. All measurements were calibrated with *iR*-compensation. Catalyst loading: 0.3 mg cm^−2^. Rotation speed: 1600 rpm. The overpotential of Ir-NSG was determined from LSV curve obtained at a scan rate of 5 mV s^−1^ and other values were plotted from references in Supplementary Table 1 and Supplementary Table 4 for (**d**) and (**h**), respectively.

Inspired by the unique structural features of Ir-NSG, we evaluated its intrinsic electrocatalytic performances for both HER and OER in comparison with pristine NSG substrate (Supplementary Fig. 15) and Ir nanoclusters loaded on N-doped graphene (Ir-NG) (Supplementary Figs. 16 and 17) in aqueous 1 M phosphate buffer saline (PBS), 0.1 M HClO_4_ and 1 M KOH electrolytes on a rotating disk electrode (RDE). Among these conditions, accelerating kinetics of HER and OER in neutral-pH media, which is more environmentally benign with practical perspectives, is most challenging, as catalysts in neutral-pH media usually exhibit 2‒3 orders of magnitude lower in activities as compared with those operated in acidic or basic media^36,37^. In this regard, we specifically focused on HER and OER under neutral-pH conditions in this work. We first conducted the HER tests of Ir-NSG (7.33 wt.%) in Ar-saturated 1 M PBS electrolyte at ambient conditions, reference measurements of commercial Pt/C (20 wt.%) were also provided as benchmarks under the same conditions (Fig. 2a). The overpotential of Ir-NSG at current density of 10 mA cm^−2^ is ~22 mV and similar to that of Pt/C (19 mV), whereas at an overpotential of 15 mV, Ir-NSG generates a mass specific activity of 299.52 mA mg_Ir_^−1^, which is 2.6 times larger than that of Pt/C (114.92 mA mg_Pt_^−1^) (Fig. 2b). The former merit evaluates the potential for practical applications, whereas the latter reveals the intrinsic electrocatalytic activity. In addition, the corresponding Tafel plots were recorded to assess the underlying reaction kinetics (Fig. 2c). The resulting Tafel slope of Ir-NSG is ~21.2 mV decade^−1^ and slightly larger than that of Pt/C (20.1 mV decade^−1^), suggesting that HER pathways for both catalysts follow the Volmer-Tafel mechanism with discharging of two adsorbed hydrogen atoms (Tafel) as the rate-determining step (RDS)^38,39^. The HER activities of Ir-NSG were also investigated in Ar-saturated 0.1 M HClO_4_ and 1 M KOH electrolytes (Supplementary Figs. 18 and 19), where it only requires an overpotential of 17 and 18.5 mV to deliver a current density of 10 mA cm^−2^, respectively, which are lower than those of Pt/C (20 and 23.5 mV, respectively). The Ir-NSG also displays low Tafel slopes of 19.2 and 28.3 mV decade^−1^ in acidic and alkaline media, comparable to those of Pt/C (19.6 and 27.9 mV decade^−1^) and illustrate the same HER mechanism with the desorption of adsorbed hydrogen as the RDS as discussed before. Impressively, the Ir-NSG exhibits lower onset overpotentials as well as higher mass specific activities of 333.51 and 382.39 mA mg_Ir_^−1^ at an overpotential of 15 mV in acidic and alkaline solution, which are 4.5 and 3.9 times greater than those of Pt/C, respectively, indicating a higher intrinsic HER activity of Ir-NSG. Notably, the HER catalytic activity of Ir-NSG is also superior to most of the recently reported excellent HER catalysts (Fig. 2d, Supplementary Figs. 18d and 19d, Supplementary Tables 1–3). Furthermore, the stability of Ir-NSG for HER was examined by chronopotentiometry in 1 M PBS, 0.1 M HClO_4_, and 1 M KOH electrolytes by holding at a constant current density of −45.47 mA mg_Ir_^−1^ as depicted in the insets of Fig. 2a, Supplementary Figs. 18a and 19a, respectively. In all three conditions, Ir-NSG is demonstrated as a very robust electrocatalyst with negligible degradation of activity after 10 h of continuous reaction.

Besides HER, the as-prepared Ir-NSG also displays remarkable intrinsic OER activity in 1 M PBS as shown in the representative polarization curve (Fig. 2e), where a sharp OER onset can be evidently observed. As displayed in Fig. 2f, Ir-NSG delivers a current density of 10 mA cm^−2^ at an overpotential of 307 mV, much lower than that for the commercial Ir/C (20 wt.%) OER catalyst (411 mV). Furthermore, Ir-NSG achieves a specific OER activity of 393.42 mA mg_Ir_^−1^ at 1.53 V vs. RHE, which is 17.6-fold higher than that for Ir/C (22.35 mA mg_Ir_^−1^), verifying a dramatic improvement of the intrinsic catalytic activity. In addition, Ir-NSG also possesses a lower Tafel slope of 74.2 mV decade^−1^ as compared with 149.2 mV decade^−1^ for Ir/C (Fig. 2g), indicating better OER kinetics of Ir-NSG. When electrolyte is switched to 0.1 M HClO_4_ and 1 M KOH, the comparisons of the overpotentials to drive 10 mA cm^−2^, the Ir-mass specific activities at 1.53 V vs. RHE, and the Tafel slopes in the low overpotential region confirm Ir-NSG as an outstanding OER catalyst at all pH conditions (Supplementary Figs. 20 and 21). Indeed, the OER activity of Ir-NSG surpasses most of the state-of-the-art OER catalysts reported in recent literatures as listed in Fig. 2h, Supplementary Fig. 20d as well as Supplementary Tables 4 and 5. In addition, we further probed the long-term electrochemical durability via chronopotentiometry at 45.47 mA mg_Ir_^−1^ in 1 M PBS, 0.1 M HClO_4_, and 1 M KOH electrolytes, respectively, finding that the real-time potential keeps nearly constant over a continuous operation of 6000 s, which establishes the high stability of Ir-NSG for OER in a wide pH range (insets in Fig. 2e, Supplementary Figs. 20a and 21a).

### Experimental approaches to probe HER and OER intermediates

**Fig. 3:**
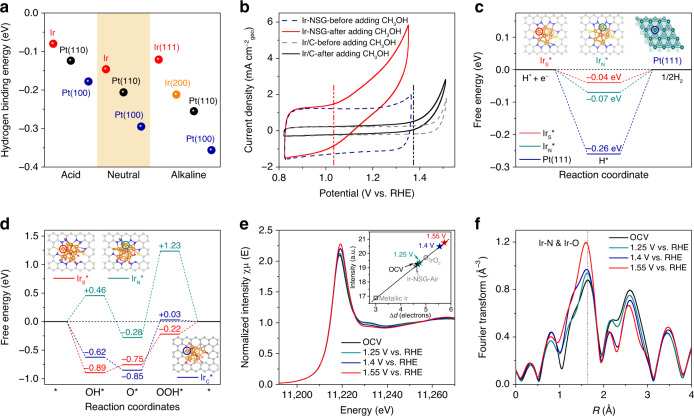
Free-energy calculations and operando X-ray absorption spectroscopy. **a** Comparison of HBE for Ir-NSG and Pt/C in a full range of pH. **b** Steady-state CV curves of Ir-NSG and Ir/C in 1 M PBS with (solid line) and without (dotted line) 0.1 M methanol recorded at a sweep rate of 50 mV s^−1^. The dash-dot lines serve as the base for the onset potential of methanol oxidation. **c** DFT calculation of the predicted free-energy diagrams for HER at *U* = 0 V on Ir_S_*, Ir_N_*, and Pt (111). **d** DFT calculation of the predicted free-energy diagrams for OER at *U* = 1.23 V on Ir_S_*, Ir_N_*, and Ir_C_* sites. The balls in gray, blue, yellow, pink, red, orange, and cyan represent C, N, S, H, O, Ir, and Pt atoms, respectively. **e** Normalized operando Ir L_3_-edge XANES spectra at various biases (applied potential versus RHE) in 1 M PBS at ambient conditions. Inset shows integrated WL intensities as a function of 5*d*-band holes derived from Supplementary Fig. 30, ex situ data for Ir/C, Ir-NSG, and IrO_2_ are provided as the reference. **f** *k*^2^-weighted Fourier transform magnitudes of EXAFS spectra collected at the Ir L_3_-edge of Ir-NSG under various potential biases. The dash-dot lines serve as the eye guide for comparison of the Ir-N (O) bond length. All data were collected with *i*–*R* correction.

Systematic studies combining both experimental data and DFT calculations have demonstrated that the hydrogen binding energy (HBE) provides a key descriptor for HER activities, and HER activity decreases monotonically with HBE for Tafel-limiting mechanism^40,41^. To gain a thorough insight into the principle that governs the exceptional performance of Ir-NSG in HER, correlations were constructed between HER activities and experimentally measured HBE using the CV method under the corresponding electrochemical conditions. Supplementary Fig. 22 shows the CV curves of Ir-NSG and the reference Pt/C in Ar-saturated 1 M PBS, 0.1 M HClO_4_, and 1 M KOH, which reveal well-defined patterns including adsorption/desorption of underpotentially deposited hydrogen (H_upd_) below 0.4 V (vs. RHE) and a wide double-layer potential region between 0.4 and 0.6 V (vs. RHE). As reported previously, the potential of H_upd_ desorption peak (*E*_peak_) could be directly related to the HBE of active site by Δ*H* = − *FE*_peak_^42^, which yielded an superior HBE of −0.146 eV for Ir-NSG as compared with that of Pt/C (−0.206 eV for Pt(110) and −0.295 eV for Pt(100)) in 1 M PBS^40^. Similar trends of HBEs are also observed in 0.1 M HClO_4_ and 1 M KOH electrolytes for the two samples and the results are summarized in Fig. 3a. Thus, the enhanced HER intrinsic activity of Ir-NSG can be ascribed to a weaker H chemisorption strength as compared to Pt/C, triggering acceleration of the hydrogen desorption step. Meanwhile, Ir-NSG also has a higher electrochemically active surface area (ECSA) as compared with Pt/C evidenced by a larger double-layer capacitance (*C*_dl_) as shown in Supplementary Fig. 22, leading to the more exposure of catalytic sites to the reactants.

To shed light on the origins of the superior OER activity of Ir-NSG, we experimentally measured the adsorption energy of the first activated oxygen intermediate OH* (the asterisk denotes the adsorption site) on Ir-NSG using a recently developed methanol oxidation method and compared it with that for Ir/C^43^. Figure 3b shows typical current profiles for methanol oxidation reaction in neutral-pH media. These curves reflect that the methanol oxidation onset potentials are 1.03 and 1.37 V (vs. RHE) for Ir-NSG and Ir/C, respectively, indicating a higher adsorption energy of OH* on Ir-NSG, which could be derived from a larger intermolecular hardness factor of the bond between Ir shell site and OH induced by the lower electronegativity of S ligand^35,44^. The higher OH* binding energy is supposed to correlate with binding energies of other oxygen intermediates (e.g., O* and OOH*) and hence OER activities. Moreover, the as-obtained high concentration of surficial OH* species can also accelerate OER as precursor for the formation of O^(II−δ)−^ active sites^45,46^. In acidic and alkaline electrolytes, the two samples also behave in similar manners as shown in Supplementary Fig. 23. In addition, the ECSAs of Ir-NSG and Ir/C were estimated to be ~238 and 50 m^2^ g^−1^, respectively, by the measurements of CO stripping coulometry in 1 M KOH electrolyte (Supplementary Fig. 24). The noticeably larger ECSA reveals more accessible active sites generated on Ir-NSG compared with Ir/C. These experimental observations suggest that the enhanced binding energy of oxygenated intermediates as well as the exposed large abundance of active sites contribute to the activity improvement of OER on Ir-NSG.

### Theoretical insights and operando XAFS measurements

From the polarization curves of HER and OER, it can be found that Ir-NSG can effectively boost both HER and OER in comparison with pristine NSG. Moreover, although Ir-NG can enhance the activities of HER and OER, they are still far inferior to those of Ir-NSG. All these results imply that Ir coordinated with both N and S could be the real active site in Ir-NSG. Therefore, DFT calculations were further carried out to understand the nature of reaction mechanism and the actual active sites at the atomic level for HER and OER on Ir-NSG. An Ir_13_ nanocluster embedded in N,S-doped graphene sheet (Ir_13_@NSG) with distorted icosahedral structure was chosen as a simplified model, which is mostly coordinated with N or S atoms as shown in Supplementary Fig. 25. Because Tafel step has been confirmed as the RDS for HER at all pH conditions for Ir-NSG and Pt/C, we studied the Gibbs free energy for H adsorption (Δ*G*_H*_) on all possible atomic sites of Ir_13_@NSG (Supplementary Fig. 26), and the calculated free-energy diagrams for HER on two selected active sites of Ir_13_@NSG in comparison with Pt(111) surface are presented in Fig. 3c. The Ir site coordinated with both N and S (Ir_S_*) exhibits near-zero Δ*G*_H*_ (−0.04 eV), a little smaller than that for Ir site coordinated with two N atoms (Ir_N_*) (−0.07 eV) but smaller in magnitude than that for Pt(111) surface (−0.26 eV), which is consistent with the experimental observation in Fig. 3a. Furthermore, the result of charge density difference analysis (Supplementary Fig. 27) shows obvious electron transfer from Ir sites to the adjacent N and S atoms, indicating that Ir atom is positively charged with an average Bader charge of 0.22 in Ir_13_@NSG. Thus, compared with metallic Pt, the H adsorption/desorption on surficial Ir active sites of Ir-NSG can be balanced by the coordinated N and S atoms via electronic modulation^47,48^.

The phenomenon of oxygen pre-adsorption on non-oxide active sites during OER has been reported and is evidenced by operando EXAFS spectroscopy in Fig. 3f^49^. In this regard, Ir_13_O_11_@G and Ir_13_O_11_@NSG configurations were constructed to investigate the effect of coordination environment on OER performance as shown in Supplementary Fig. 28. Figure 3d depicts the predicted free-energy profiles for OER on Ir_S_*, Ir_N_*, and Ir_C_* sites at 1.23 V, respectively. Obviously, the third proton-electron transfer step of forming OOH* from O* is the potential determining step for all the three evaluated active sites, and the Ir_S_* site possesses a remarkably lower energy barrier of 0.53 eV than that for Ir_N_* (1.51 eV) and Ir_C_* site (0.88 eV). Moreover, we can see that the decrease in the energy barrier is beneficial from the strong binding energy of OOH* on Ir_S_*, which can be correlated with the high OH* binding energy probed in methanol oxidation measurement. The projected density of states (PDOS) in Supplementary Fig. 29 reveals strong interactions between Ir_13_O_11_ cluster and the support in Ir_13_O_11_@NSG, suggesting that the coordination of Ir with N and S can be well retained during OER. In addition, there observe apparent downshifted distributions of Ir_S_* and overlaps between OOH* and Ir_S_* below the Fermi level, which represent the strongest adsorption of OOH* on the Ir_S_* site. Thus, we can conclude that the Ir site tuned by neighboring N and S atoms is responsible for the excellent OER activity owing to its favorable bindings to the OH* and OOH* intermediates.

Ir L_3_-edge XANES and EXAFS spectra on Ir-NSG were collected under operando conditions to gain insights into the intermediate electronic structure and coordination environment of active sites during OER (Fig. 3e, f). In situ XANES profiles in Fig. 3e clearly show a trend of higher WL energy position and intensity when the applied potential is stepped from open-circuit voltage (OCV) to 1.55 V vs. RHE, and the inset of Fig. 3e displays a pronounced upshift of the 5*d*-band holes count to more than five when the applied potential is increased above 1.40 V vs. RHE, corresponding to the formation of a more catalytically active type of Ir with valence state >+4 during OER process, which may be induced by the adsorption of additional oxygen on Ir sites^49–51^. Figure 3f shows the distortion of local geometric structure around Ir shell sites at applied potentials. The intensity of the peak at ~1.65 Å increases significantly owing to a contribution from the Ir-O bond, which is overlapped with the Ir-N bond, as a result of the oxidation of Ir sites during OER. Moreover, a shrinkage of Ir-O bond is observed with the bias stepping to a higher potential and can be ascribed to a shorter effective radius of Ir sites at over-oxidation states^52^. Therefore, both the substantially higher intensity and shorter distance of the Ir-N/O bond demonstrate the formation of oxo-ligands and a larger number of 5*d*-band holes for surficial Ir sites during OER. As proposed in previous literatures, increasing number of Ir 5*d* vacancies and contracted Ir-O ligands could lead to the formation of O 2*p* holes, and the electron deficient intermediate O species (generally named O^(II−δ)−^) would appear as terminal ligands^50^. Such O^(II−δ)−^ ligands with more electrophilic nature are susceptible to nucleophilic attack to facilitate the RDS of O-O bond formation, which can be considered as another key factor in the enhanced catalytic reactivity of OER for our Ir-NSG catalyst^53,54^.

### pH-universal overall water-splitting cell

**Fig. 4:**
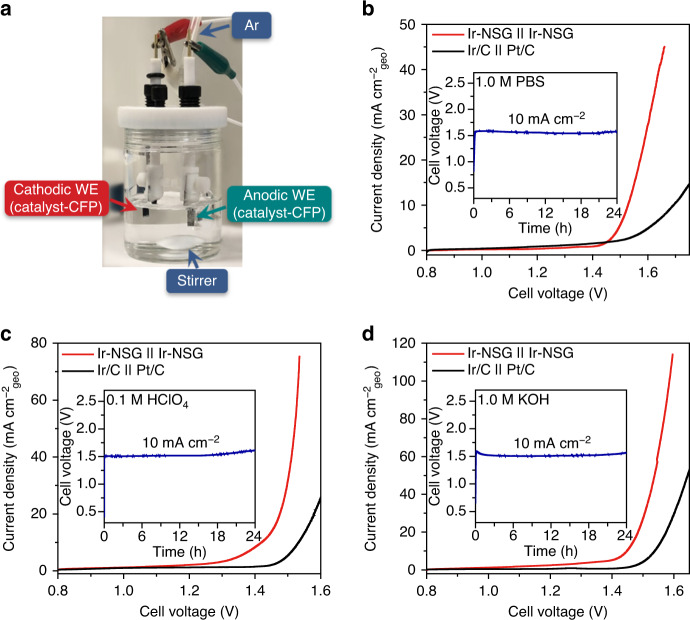
Overall water-splitting performance. **a** Digital photograph of the homemade two-electrode cell for water splitting. *WE* working electrode, *CFP* carbon fiber paper, and *Ar* pure argon gas (99.99%). LSV curves of Ir-NSG and Ir/C-Pt/C coupled water electrolysis cell at a scan rate of 5 mV s^−1^ in **b** 1 M PBS, **c** 0.1 M HClO_4_, and **d** 1 M KOH. Inset shows the corresponding chronopotentiometric curves at 10 mA cm^−2^. Catalyst loading: 73.3 μg_metal_ cm^−2^. All measurements were conducted in Ar-saturated electrolytes and corrected for *i*–*R* drop.

On the basis of the above results, we propose that decorating Ir nanoclusters onto N,S-doped graphene provides a favorable mesoporous morphology, surface electronic structure and coordination environment that synergistically enable advanced pH-universal water reduction and oxidation catalysis. To demonstrate the practical applications, we loaded the Ir-NSG catalyst on carbon fiber paper (CFP) as both the anode and cathode to construct a two-electrode water electrolysis cell in 1 M PBS, 0.1 M HClO_4_, and 1 M KOH solutions and acquired their activities by linear sweep voltammograms (LSVs) as shown in Fig. 4. Remarkably, the cell with the Ir-NSG anode and cathode requires only 1.53, 1.42, and 1.45 V to reach a current density of 10 mA cm^−2^ in neutral, acidic, and alkaline electrolyzers, respectively, outperforming the coupled benchmark Ir/C | | Pt/C electrolyzer (Fig. 4b–d). Specially, we further explored the water electrolysis performance at large current densities (500 and 1000 mA cm^−2^) as shown in Supplementary Fig. 31, highlighting the potential of Ir-NSG for practical overall water splitting. Insets in Fig. 4b–d manifest that the electrolyzer assembled with Ir-NSG anode and cathode can retain a current density of 10 mA cm^−2^ over long-term continuous tests with slight voltage decay and no detectable structure degradation at all pH values (Supplementary Fig. 32), which is among the most robust overall water-splitting catalysts reported to date, especially in acidic media (Supplementary Table 6). The excellent pH-universal activity and stability of overall water splitting make Ir-NSG as a potential candidate for practical water electrolysis applications with satisfactory operability, safety, and environmental friendliness.

## Discussion

In summary, we have developed uniformly dispersed, ultrafine, and coordination-engineered Ir nanoclusters anchored on N,S-doped graphene as an outstanding pH-universal bifunctional electrocatalyst for both HER and OER, which requires voltages of as low as 1.53, 1.42, and 1.45 V to reach a geometric current density of 10 mA cm^−2^ for overall water splitting in neutral, acidic, and alkaline electrolytes, respectively. Based on experimental investigations and theoretical calculations, we conclude that the impressive activities originate from the optimized adsorption energy and states of intermediates, which can be ascribed to the flexible redox states and coordination ligands on the surficial sites of Ir nanoclusters. Accordingly, the discovery of Ir-NSG catalyst offers a unique insight into the guiding principle for the rational design of efficient active sites by modulating electronic and geometric structure to tailor the binding of intermediates. We anticipate that these findings will pave the way for the development of renewable energy applications in future.

## Methods

### Chemicals

Melamine (99%, CAS no. 108-78-1), l-cysteine (≥98%, CAS no. 52-90-4), iridium(III) chloride hydrate (99.9% trace metals basis, CAS no. 14996-61-3), sodium phosphate dibasic (≥99.0%, CAS no. 7558-79-4), sodium phosphate monobasic (≥99.0%, CAS no. 7558-80-7), perchloric acid (70%, CAS no. 7601-90-3), potassium hydroxide (pellets, 99.99% trace metals basis, CAS no. 1310-58-3), methanol (≥99.9%, CAS no. 67-56-1), platinum on Vulcan XC72 powder (20 wt.% loading), iridium on Vulcan XC72 powder (20 wt.% loading), iridium(IV) oxide (99.9% trace metals basis, CAS no. 12030-49-8), isopropanol (99.5%, CAS No. 67-63-0) and nafion 117 solution (~5% in a mixture of lower aliphatic alcohols and water) were purchased from Sigma-Aldrich and used without further purification.

### Synthesis of Ir-NSG catalyst

In a typical synthesis of Ir-NSG, a mixture of melamine (C_3_H_6_N_6_) (8 g), l-cysteine (C_3_H_7_NO_2_S) (1.5 g) and iridium(III) chloride hydrate (IrCl_3_·xH_2_O) (37.5 mg) was first ground into a homogeneous precursor using ball-milling in a nylon jar. Subsequently, the fine powder mixture was undergone a two-stage pyrolysis in a tubular furnace (Carbolite, UK) under argon atmosphere. The first stage was from 25 to 600 °C at a ramping rate of 2.5 °C min^−1^ and maintained at 600 °C for 2 h, whereas the second stage was from 600 to 800 °C at a ramping rate of 2.5 °C min^−1^ and maintained at 800 °C for 1 h. After cooling to room temperature, the product was washed thoroughly with deionized water and ethanol and dried at 60 °C in an oven overnight.

### Characterization

The morphological information was collected by FESEM (JEOL JSM-6700F) and TEM (JEOL JEM-2100F). Specific surface area was measured based on a N_2_ adsorption–desorption method using Autosorb-6B (Quantachrome) at 77 K. Sub-ångström-resolution high-angle annular dark-field scanning transmission electron microscopy (HAADF-STEM) characterization was conducted on a JEOL JEMARM200F STEM/TEM with a guaranteed resolution of 0.08 nm. EELS spectra were collected using a Gatan imaging filter (Quantum 965). Elemental analysis was performed with a Thermo Scientific Flash 2000 analyzer. The crystal structure was examined by XRD (Bruker AXS D8 Advance) using Cu Kα radiation (*λ* = 1.5406 Å) with a LYNXEYE detector at 30 kV and 10 mA. The mass content of Ir in Ir-NSG was quantified by ICP-AES (PerkinElmer). Detailed chemical compositions were analyzed by XPS on an ESCALAB 250 photoelectron spectrometer (Thermo Fisher Scientific) using a monochromatic Al Kα X-ray beam (1486.6 eV). All binding energies were calibrated to the C 1*s* peak (284.6 eV) arising from the adventitious carbon-containing species. XAFS spectroscopy measurements including XANES and EXAFS were performed by employing synchrotron radiation light source at BL-12B2 beam line of the Japan Synchrotron Radiation Research Institute (JARSI) in SPring 8 (Japan), in which the electron storage ring was operated at 8.0 GeV. The data were collected at Ir L_3_-edge (11215 eV) with the samples held at ambient condition and the EXAFS spectra were fitted using the FEFF 6.0 code.

Operando XAFS measurements were conducted in a specially designed Teflon container with a window sealed by Kapton tape under identical conditions as the electrochemical measurements. For the operando experiments, the cell was filled with electrolyte (1 M PBS), Pt foil and saturated calomel electrode (SCE) were used as the counter and reference electrode, respectively. The catalyst was hand-brushed onto a CFP electrode with the loading amount of 1 mg cm^−2^, followed by pressing the electrode between the electrolyte compartment of the cell. OCV, 1.25 V vs. RHE, 1.4 V vs. RHE, and 1.55 V vs. RHE were chosen as four representative conditions. The electrode was maintained under each condition for 30 min before measuring the spectrum. The Ir L_3_-edge XANES and EXAFS spectra were collected in total-fluorescence-yield mode at room temperature using BL-12B2 beam line at Spring-8, JARSI.

### Electrochemical measurements

All electrochemical measurements were carried out at ambient temperature and pressure with a CHI 760e potentiostat. Glass cell was utilized in neutral and acidic media, whereas in alkaline media, all cell components were plastic in order to prevent contaminations from Fe leaching. To mitigate impurities from previous experiments, cells and other glassware were thoroughly cleaned by soaking in aqua regia when not in use and rinsed with water and corresponding electrolyte reagent for several times prior to use. 1 M PBS was prepared by diluting a mixture of 0.62 mol Na_2_HPO_4_ and 0.38 mol NaH_2_PO_4_ to 1 L using ultrapure water (15 MΩ, Milli-Q). The 0.1 M HClO_4_ and 1 M KOH electrolyte solutions were prepared from appropriate 70 wt.% double-distilled HClO_4_ and KOH pellets with ultrapure water, respectively. Specially, for all HER tests, the electrolytes were saturated with Ar (99.99%) by purging Ar into the aqueous solutions for 30 min, and then maintaining the flow of Ar throughout the entire electrochemical measurements. It should also be noted that Ir might catalyze the reduction of ClO_4_^−^ ions into Cl^−^ in a potential range where ClO_4_^−^ ions were adsorbed on the catalyst surface. In order to keep chloride species at a reasonably negligible level, measurements in acidic media were performed within 2–3 hours and a potential of 0.4 V (vs. RHE) was imposed during all holding periods. Electrocatalyst inks were prepared by dispersing 5 mg of catalyst into a solution containing 25 μL of 5% Nafion 117 solution (as conducting binder) and 975 μL of ultrapure water-isopropanol solution with equal volumes of water and isopropanol, followed by ultrasonication for 3 h. A three-electrode cell configuration was employed with a working electrode of glassy carbon RDE with 5 mm diameter, a counter electrode of graphite rod and a SCE as the reference electrode, the tip of the reference electrode was placed close to the working electrode to minimize solution resistance. Before each experiment, the glassy carbon electrode was polished to mirror shine with 0.05 μm alumina and cycled ~50 times from −0.2 to 1.5 V (vs. RHE) at a sweep rate of 300 mV s^−1^ in 0.5 M H_2_SO_4_. Then an aliquot of 12 μL of the catalyst ink was drop-casted on the glassy carbon electrode (catalyst loading: 0.3 mg cm^−2^) and allowed to dry in air. To ensure the accuracy of reversible hydrogen electrode (RHE), we calibrated the reference electrode in H_2_-saturated 1 M PBS, 0.1 M HClO_4_, and 1 M KOH electrolytes periodically before conducting experiments. A RHE was performed using two Pt plates as both the working and counter electrode (cleaned in aqua regia prior each use) with H_2_ bubble over the working electrode during the period. To operate the calibration, the clean Pt electrode is cycled ± (5–15) mV at a slow sweep rate of 10 mV s^−1^ around the expected value for the RHE in each electrolyte to be used for the catalyst testing until the CV curve reached constant. Then the RHE potentials in the three electrolytes were determined from the corresponding open-circuit potentials (−0.629 V for 1 M PBS, − 0.299 V for 0.1 M HClO_4_ and −1.056 V for 1 M KOH, respectively) as shown in Supplementary Fig. 33. All electrode potentials reported herein were converted to the RHE scale using *E*(vs. RHE) = *E*(vs. SCE) + 0.629 V, *E*(vs. RHE) = *E*(vs. SCE) + 0.299 V and *E*(vs. RHE) = *E*(vs. SCE) + 1.056 V for the measurements in neutral, acidic and alkaline media, respectively. The overpotential *η* was calculated by *η* = *E*(vs. RHE) V for HER and *η* = *E*(vs. RHE) –1.23 V for OER, respectively. In addition, for all the *i*–*E* curves reported in this study, the solution resistance *R* was measured via *iR* compensation command by applying the test potential, step amplitude, compensation level and overshoot level as 0 V, 0.05 V, 100% and 2%, respectively, which was subsequently used to correct the solution Ohmic loss by *E* = *E*_measured_ – *iR*. The reported current densities were either normalized to the geometrical area of electrode (mA cm_geo_^−2^) or the amount of the metal in the catalyst (mA mg_metal_^−1^).

Several fast CV scans (50 mV s^−1^) between 0 and 1.4 V (vs. RHE) were applied to remove the surface contaminants and electrochemically activate the catalysts to achieve a stable performance before each measurement. In cases where we aimed to assess the activity from polarization curves, we performed CV measurements at a rotating speed of 1600 rpm and a very low scan rate of 1 mV s^−1^ and obtained steady polarization curves based on an average of the current from the forward and reverse sweeps to eliminate the capacitive background, which mainly originated from the large specific surface area of the graphene substrate and ultra-small size of Ir nanoclusters. We collected the log current-overpotential (Tafel) data using controlled potential electrolysis. The electrode potentials were adjusted from high to low values with a fixed decrement across the linear Tafel region. At each potential step, data was collected until steady state was reached where the current did not change with time. All measurements were repeated for three times to ensure reproducibility.

CO stripping measurements in 1 M KOH were utilized to determine the ECSAs of Ir-NSG and Ir/C. The electrolyte solution was first purged with CO (99.99%) for 5 min to poison the catalyst sufficiently, followed by purging Ar (99.99%) for another 5 min to remove any excess CO from the solution. Then the first two cycles of CV curves were collected at a scan rate of 50 mV s^−1^ for further calculation. The ECSAs were estimated using CO* stripping coulometry by assuming a charge density of 420 μC cm_Ir_^−2^ for electrooxidation of one CO* monolayer.

### Computational details

DFT calculations were performed using the Vienna Ab-initio Simulation Package^55,56^ with the projector augmented wave (PAW) method^57^ and a cutoff kinetic energy of 400 eV for plane-wave basis set. The generalized gradient approximation (GGA) with PBE functional^58^ was used. An energy difference within 1.0 × 10^−5^ eV and force threshold of 0.02 eV Å^−1^ for the maximal component were set as the convergence criteria for solving for wavefunctions and geometry optimization, respectively. The reciprocal Brillouin zones were sampled by the *Γ* point as the unit cell is sufficiently huge. Because we mainly focus on the role of the first coordination shell in this work, an icosahedral Ir_13_ was used as the simplified model of the iridium nanocluster and Ir_13_ placed in one hole of a 9 × 9 graphene sheet was used for Ir_13_@G. The hole of Ir_13_@G was created by removing twelve carbon atoms of graphene to capture Ir_13_. The twelve carbon atoms coordinated to Ir_13_ were replaced by ten nitrogen atoms and two sulfur atoms to mimic the doping system abbreviated as NSG. As the iridium nanocluster is oxidized for OER, twelve oxygen atoms were attached to the iridium atoms at the surface of Ir_13_ to simulate the oxidized cluster. Thus, Ir_13_O_11_@G or Ir_13_O_11_@NSG with one iridium active site was described as the catalyst for OER.

The Tafel slopes of Ir-NSG and Pt/C for HER indicate that the Tafel step (2H* → H_2_ + 2*) is the RDS. Thus, we considered the widely used H adsorption as the key descriptor for the HER activity^41^. As introduced by Nørskov and co-workers^59^, the OER mechanism was described by a four-electron transfer process with the following steps, where the symbol “*” represents the active site.

1 $$\ast + {\mathrm{H}}_2{\mathrm{O}} \to {\mathrm{OH}}^ \ast + {\mathrm{H}}^ + + {\mathrm{e}}^ -$$

2 $${\mathrm{OH}}^ \ast \to {\mathrm{O}}^ \ast + {\mathrm{H}}^ + + {\mathrm{e}}^ -$$

3 $${\mathrm{O}}^ \ast + {\mathrm{H}}_2{\mathrm{O}} \to {\mathrm{OOH}}^ \ast + {\mathrm{H}}^ + + {\mathrm{e}}^ -$$

4 $${\mathrm{OOH}}^ \ast \to \ast + {\mathrm{O}}_2 + {\mathrm{H}}^ + + {\mathrm{e}}^ -$$

To derive the reaction free energy (∆*G*), differences in zero-point energy (∆ZPE) and entropy effects are taken into account as follows: ∆*G* = ∆*E* + ∆ZPE – *T*∆*S*, where ∆(*PV*) = 0 for the solution system. The reaction energy ∆*E* is available from quantum mechanical calculations (e.g., DFT) and change in entropy (∆*S*) at *T* = 298.15 K is obtained from vibrational frequency calculations. Free energies are used for all absorbed species^41,60^, whereas the corrections for gas phase molecules are taken from standard thermodynamics tables^61^. The standard hydrogen electrode (SHE) is used as the reference electrode to define the potential. The free-energy change of 1/2H_2_ → H^+^ + e^−^ reaction will be zero at the potential of 0 V and 1/2 *G*(H_2_) is used to represent the free energy of proton and electron. The free energy of gaseous O_2_ is derived as *G*(O_2_) = 2 *G*(H_2_O) – 2 *G*(H_2_) + 4.92 (eV)^62^. The free energy at an applied overpotential *U* is calculated as *G*_U_ = *G* – *n*e*U*, where *n* is the number of (H^+^ + e^−^) pairs involved and e is the transferred electron.

## Supplementary information

Supplementary Information

Peer Review File

## Data Availability

The data that support the findings of this study are available from the corresponding author upon reasonable request.
