# Supplementary material for: Coordination engineering of iridium nanocluster bifunctional electrocatalyst for highly efficient and pH-universal overall water splitting
Source: Nat Commun. 2020 Aug 25;11:4246. doi: 10.1038/s41467-020-18064-w (PMC7447631; doi:10.1038/s41467-020-18064-w)
Supplement: Supplementary file 1 — Supplementary Information [file 41467_2020_18064_MOESM1_ESM.pdf]

Supplementary Information

Coordination engineering of iridium nanocluster bifunctional electrocatalyst for highly efficient and pH-universal overall water splitting

Wang et al.

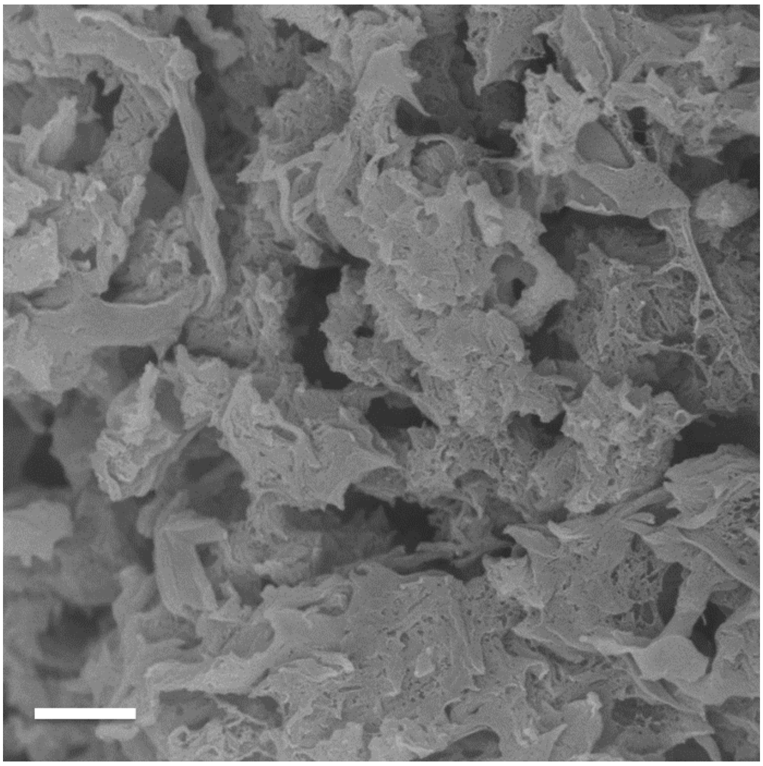

Supplementary Figure 1. A typical SEM image showing the as-prepared Ir-NSG. Scale bar is 1 μm.

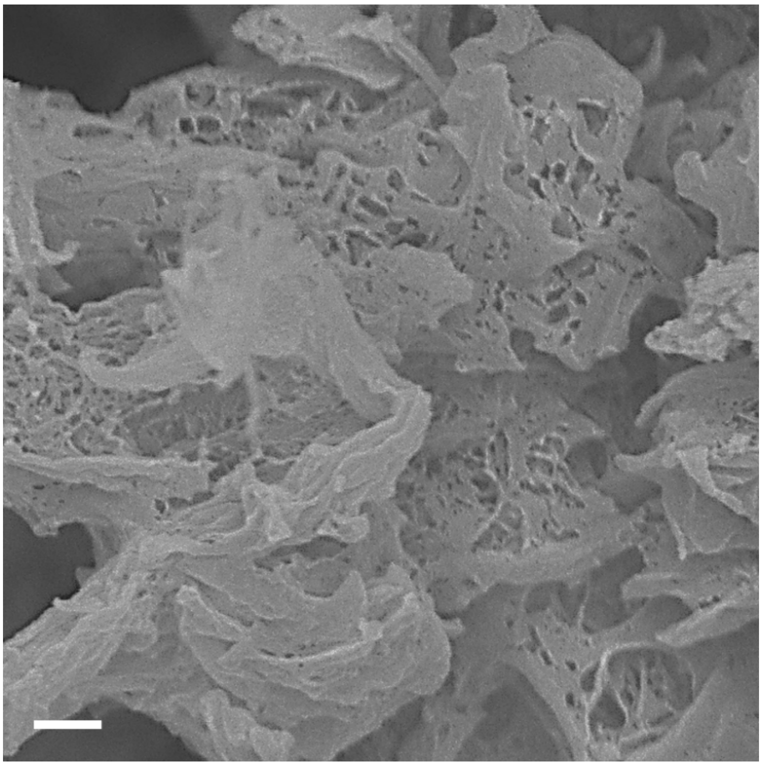

Supplementary Figure 2. High magnification SEM image of the as-prepared Ir-NSG. Scale bar is 200 nm.

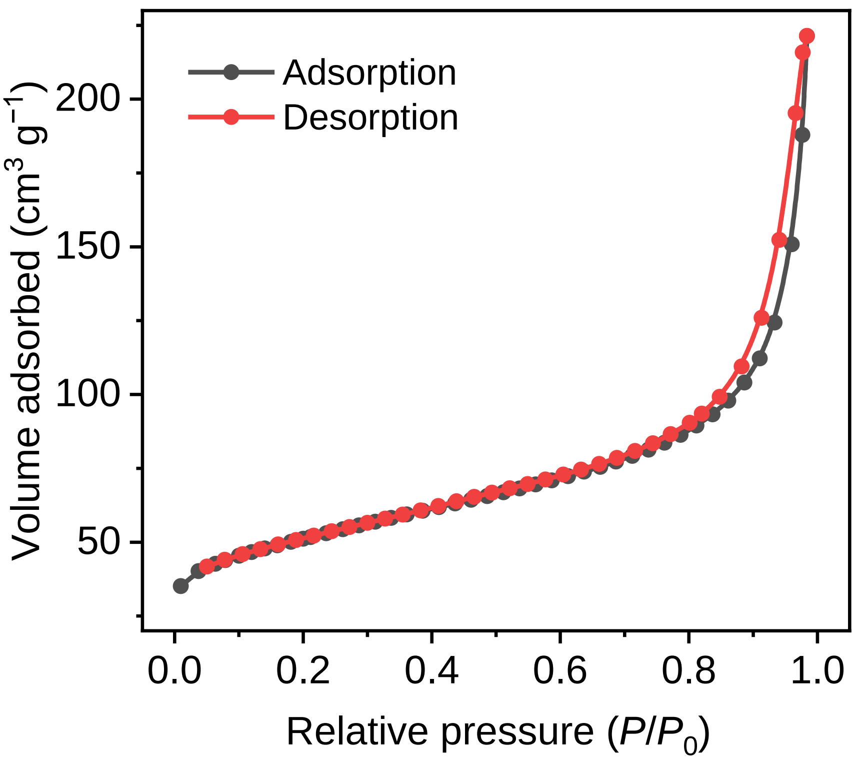

Supplementary Figure 3. Nitrogen adsorption-desorption isotherm of commercial Ir/C measured at 77 K.

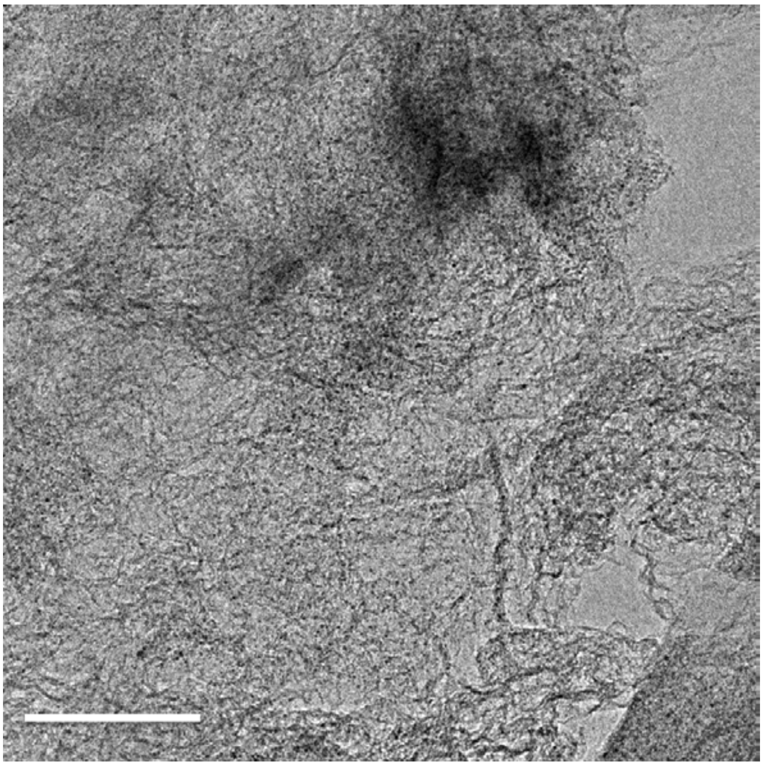

Supplementary Figure 4. A bright-field TEM image showing the Ir-NSG. Scale bar is 100 nm.

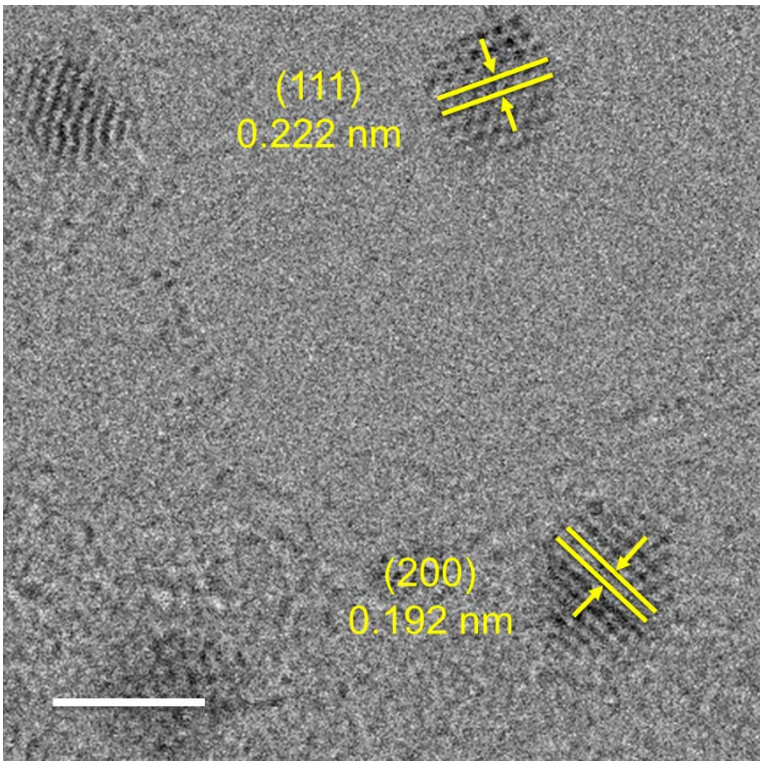

Supplementary Figure 5. A bright-field atomic-resolution STEM image of Ir-NSG. Scale bar is 2 nm.

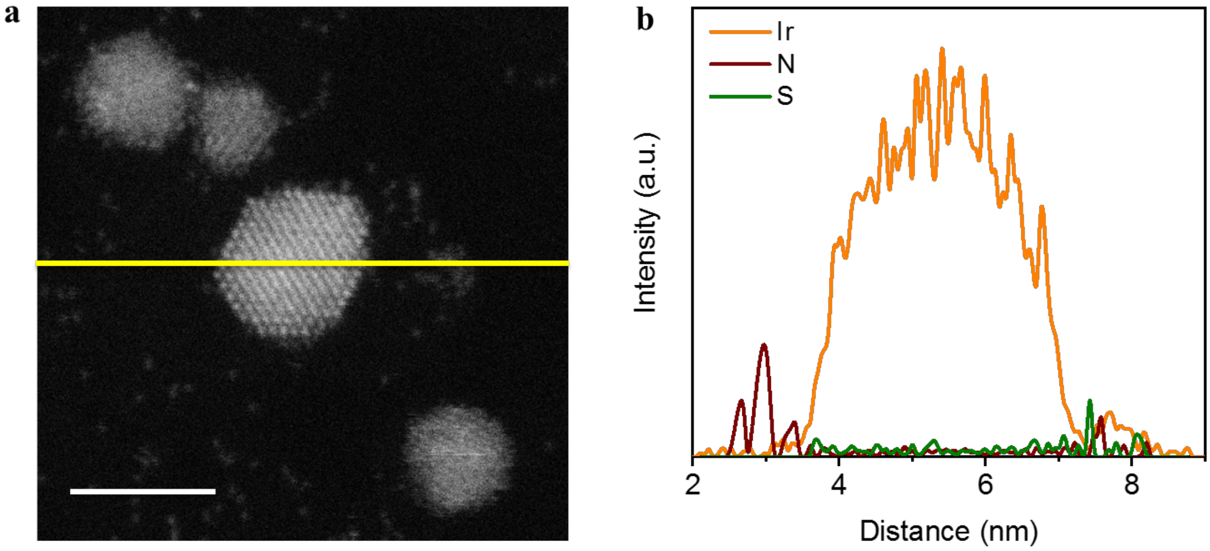

Supplementary Figure 6. Line scans of Ir-NSG. a, Aberration-corrected high-angle annular dark field (HAADF) STEM image of Ir-NSG. Scale bar is 3 nm. **b,** The corresponding intensity profile of Ir, N and S elements along the line as shown in **a**. The intensities of N and S were graphed relative to that of Ir.

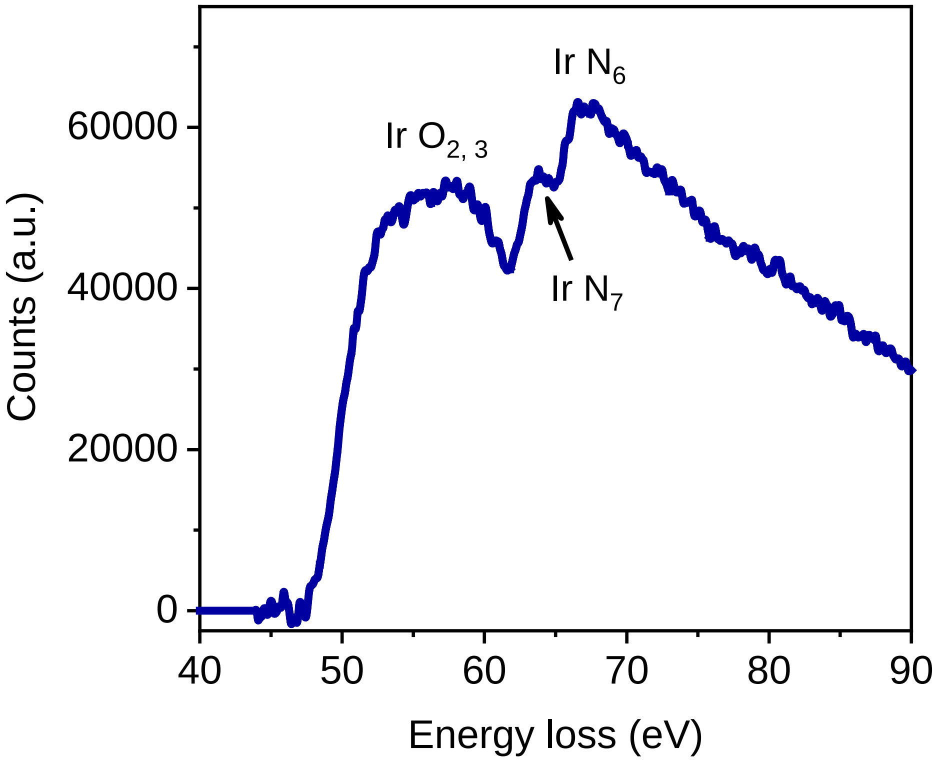

Supplementary Figure 7. Power-law background subtracted EELS spectrum of Ir-NSG. The labels of Ir $N_{6,7}$ and Ir $O_{2,3}$ represent the fourth and fifth electron shell of Ir atom, respectively.

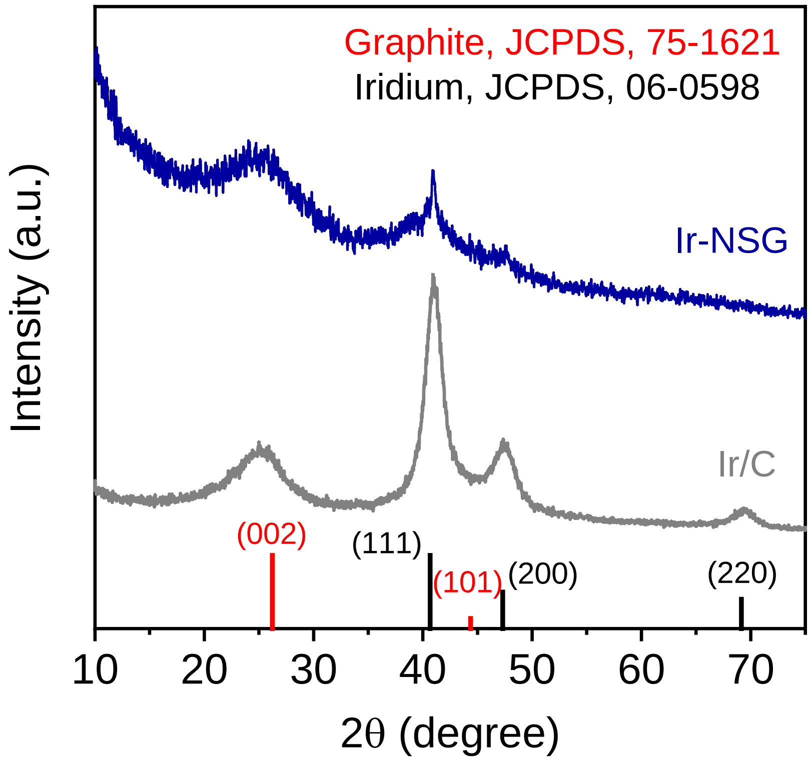

Supplementary Figure 8. X-ray diffraction patterns of Ir-NSG and commercial Ir/C. The JCPDS profiles of graphite (red) and metallic iridium (black) are displayed for reference.

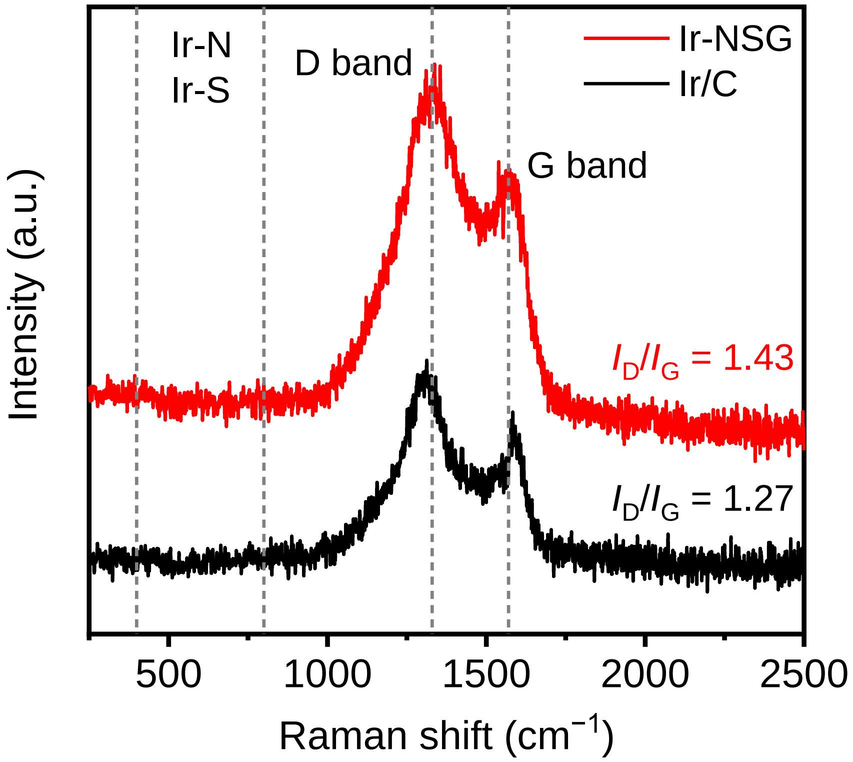

Supplementary Figure 9. Raman spectra of Ir-NSG and commercial Ir/C. No obvious vibrations are observed in the range of 400–800 cm^{-1}, implying the dispersive and ultrafine characteristic of Ir nanoclusters in the sample.

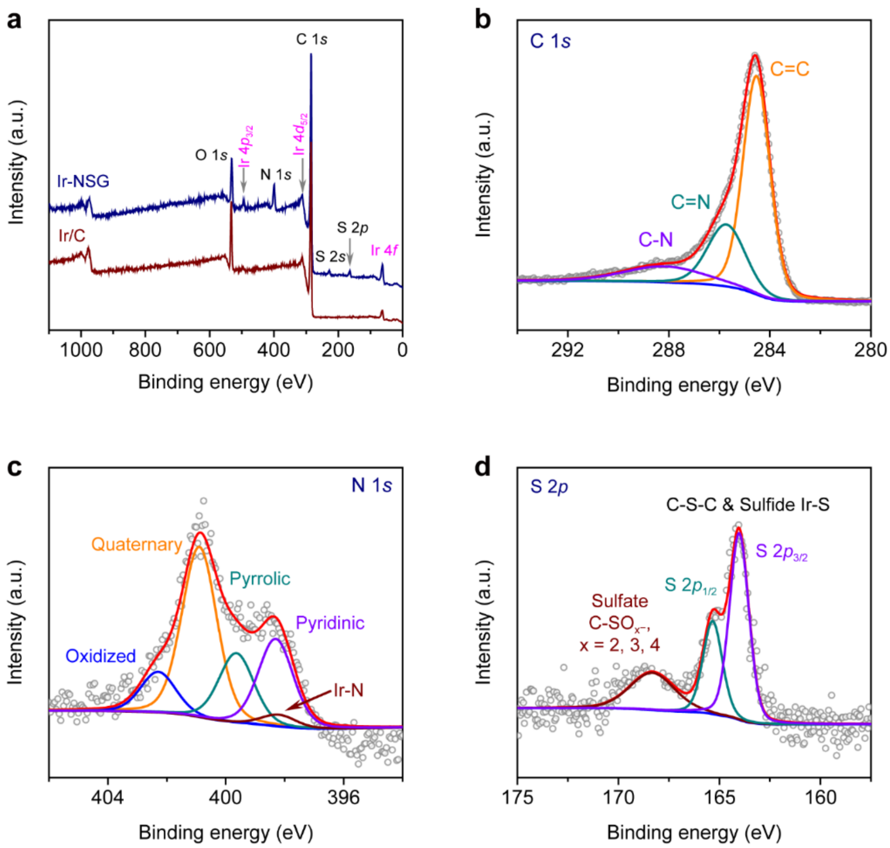

Supplementary Figure 10. XPS spectra of Ir-NSG catalyst. a, Survey spectra of Ir-NSG and commercial Ir/C. Compared to the corresponding spectrum of commercial Ir/C, the full survey spectrum of Ir-NSG confirms the presence of C, N, S, O and Ir elements, revealing the successful doping of N and S into graphene while the source of O is mostly related to the edge groups of N,S-doped graphene matrix as well as physically adsorbed and/or trapped oxygen and moisture.[1,2] **b,** High-resolution C 1*s* spectra. The peak at ~284.5 eV, which is assigned to the graphitic sp^2 C, has the largest intensity, indicating the construction of graphene structure during the pyrolysis process. On the other hand, the small peaks at 285.7 and 288.2 eV obtained by peak fitting imply the bond formation of doped N and S with sp^2-C and sp^3-C atoms, respectively.[3] **c,** High-resolution N 1*s* spectra. **d,** High-resolution S 2*p* spectra. The S $2p_{3/2}$ peak of Ir-NSG is at 163.90 eV, which is 0.10 eV left-shifted compared with pure S (S $2p_{3/2}$,164.0 eV), implying the formation of Ir-S bonds between Ir nanoclusters and surrounding S.[4] Additionally, the peak at 168.3 eV can be ascribed to the sulfate species (C-SO_x-, x=2, 3, 4) at the edge of graphene.[4,5]

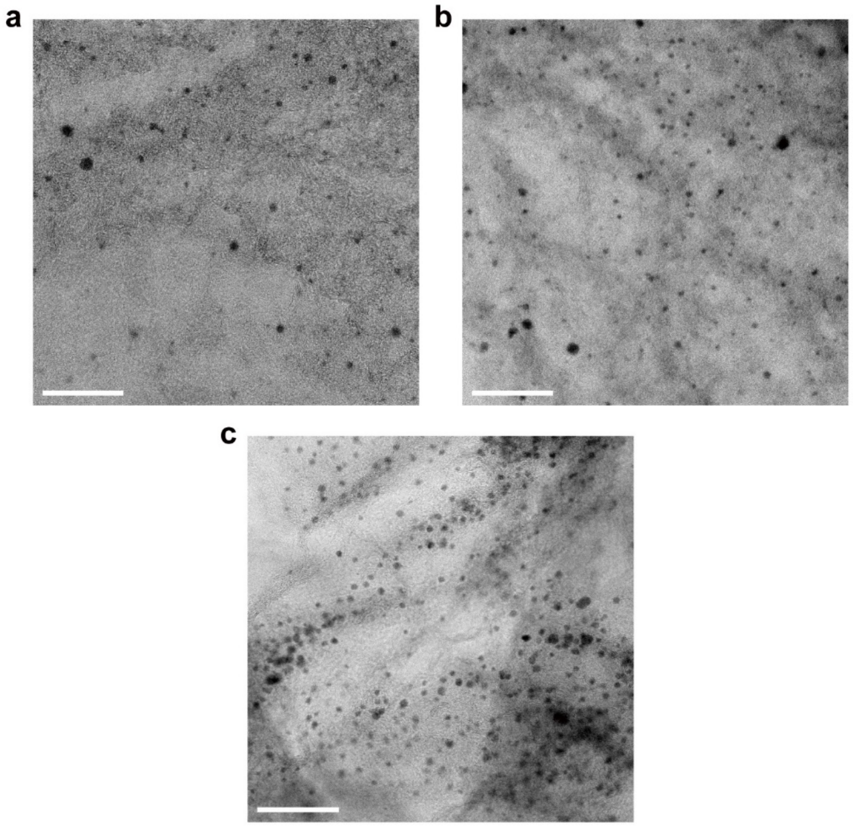

Supplementary Figure 11. Bright-field TEM images of samples with various Ir loading amounts. a, Ir-NSG-4.28 wt. %. **b,** Ir-NSG-5.97 wt. %. **c,** Ir-NSG-8.59 wt. %. Scale bars: 20 nm.

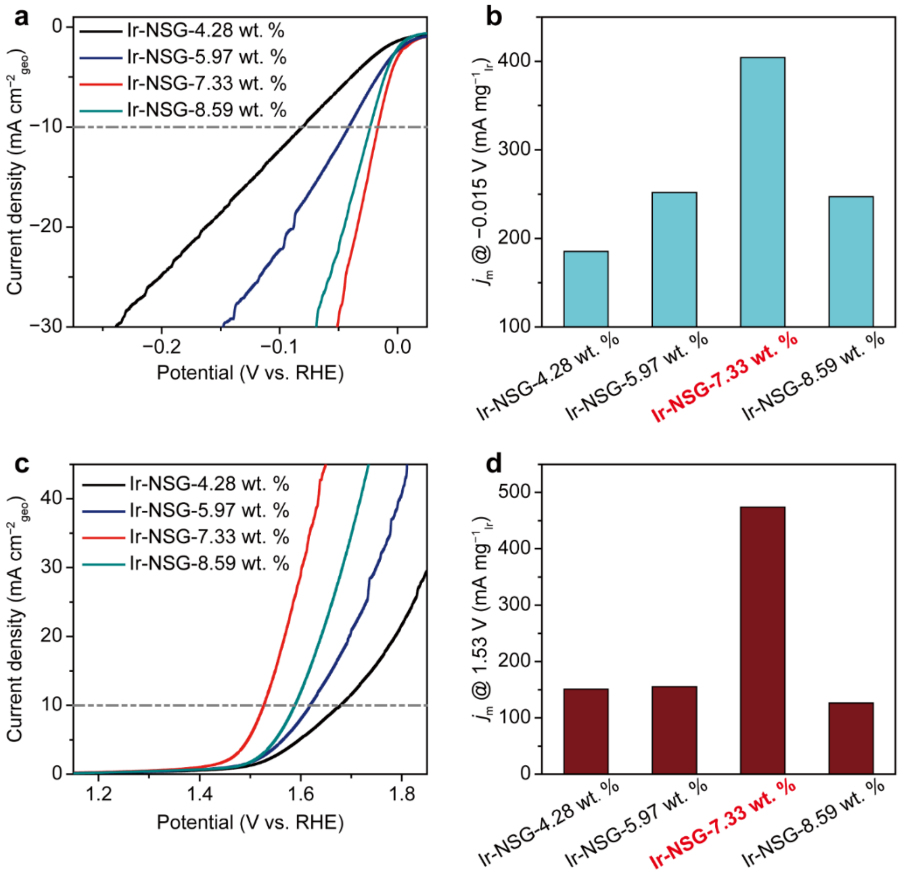

Supplementary Figure 12. HER and OER performance of Ir-NSG electrocatalysts with different Ir mass contents in neutral electrolyte. a, HER polarization curves acquired at a sweep rate of 5 mV s^{-1} in Ar-saturated 1 M PBS. The dash-dot line serves as the eye guide for the comparison of the overpotentials at 10 mA cm^{-2}. **b,** Comparison of Ir-mass specific activities at −0.015 V (vs. RHE). **c,** OER polarization curves acquired at a sweep rate of 5 mV s^{-1} in 1 M PBS. The dash-dot line serves as the eye guide for the comparison of the overpotentials at 10 mA cm^{-2}. **d,** Comparison of Ir-mass specific activities at 1.53 V (vs. RHE). As a result, Ir-NSG-7.33 wt. % is the optimized sample with the best HER and OER performance because of its lowest overpotential at 10 mA cm^{-2} and largest mass activities.

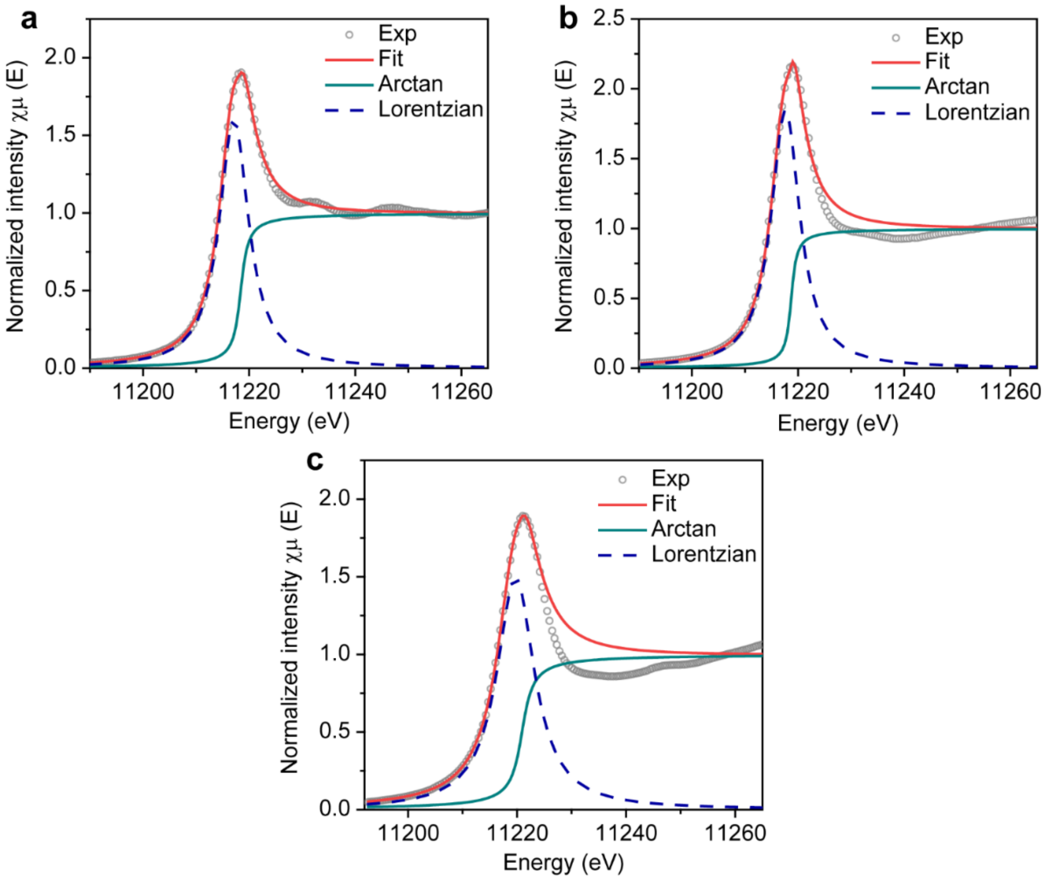

Supplementary Figure 13. Fitting of XANES spectra for Ir-based samples using a set of arctangent function (green solid line) and Lorentzian function (blue dashed line). The gray dots and red solid lines represent the experimental data and fitting curves, respectively. **a,** metallic Ir. **b,** Ir-NSG. **c,** IrO_2. The arctangent function is applied to model the continuum step at the L_3 absorption edge, which is centered at the absorption edge with a fixed unit amplitude in all fits.[6] The integral area was then calculated over the range between experimental data and arctangent function, which is approximately Lorentzian in shape.[7]

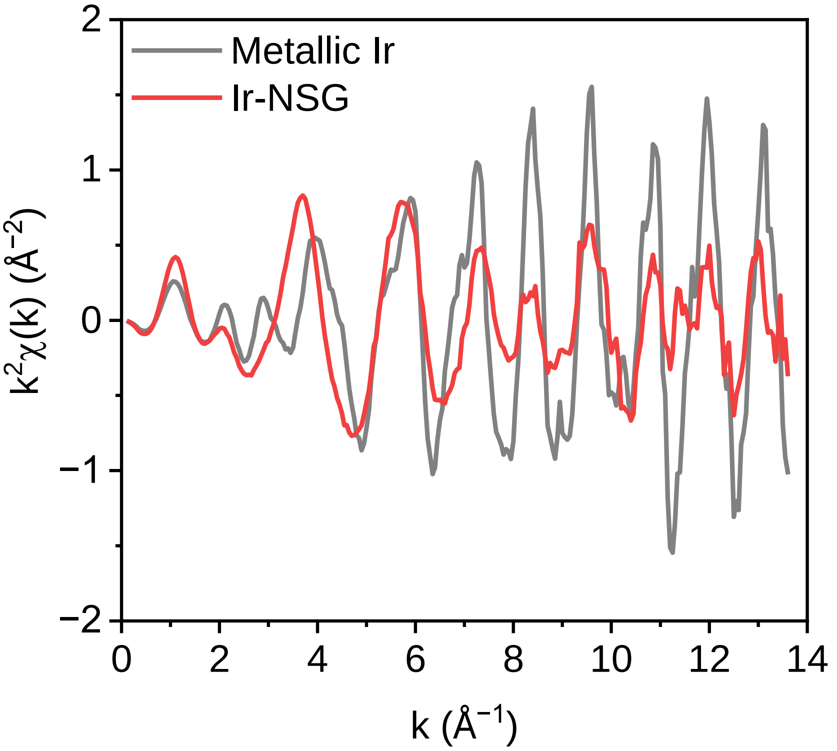

Supplementary Figure 14. k^2-weighted k-space spectra of Ir-NSG and metallic Ir.

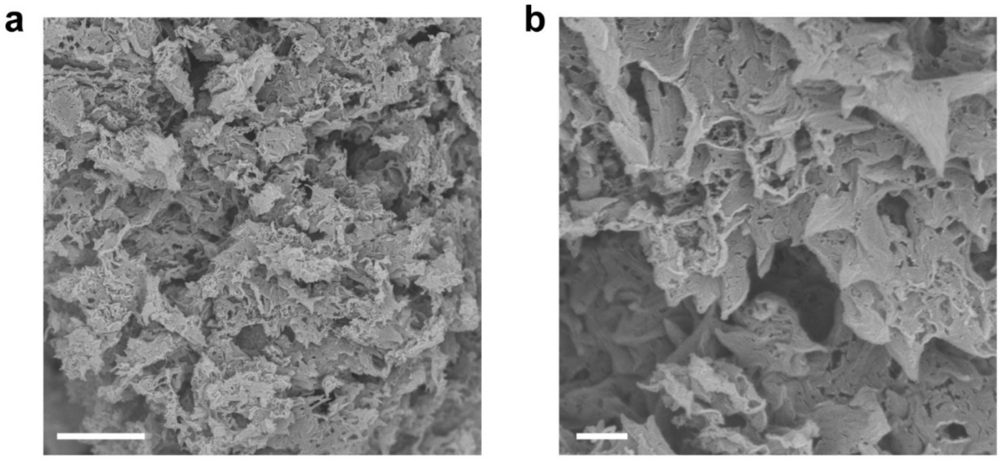

Supplementary Figure 15. Typical SEM images of NSG, showing the same morphology with Ir-NSG. Scale bars are 3 μm and 300 nm for **a** and **b**, respectively.

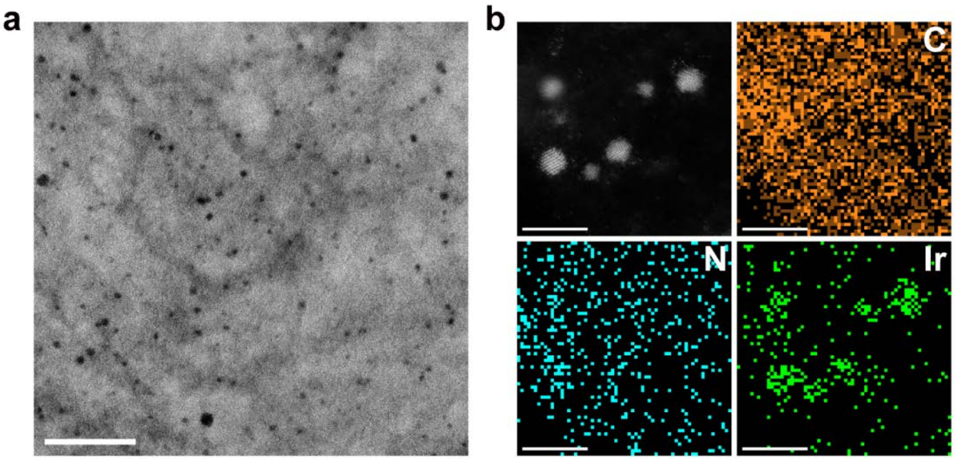

Supplementary Figure 16. Morphology of Ir-NG. a, Bright-field TEM image of Ir-NG (with 7.07 wt. %), showing that Ir nanoclusters are homogeneously distributed in the N-doped graphene framework. Scale bar: 20 nm. **b,** Dark-field STEM image and EDX elemental mappings, scale bars: 5 nm.

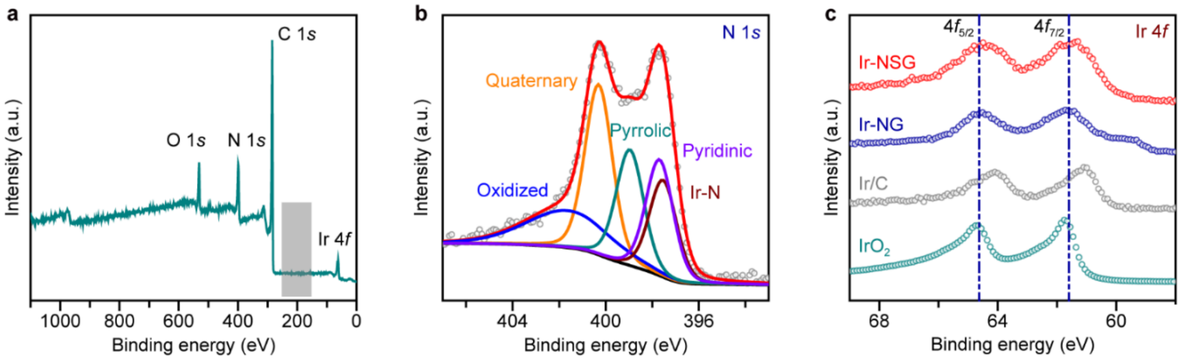

Supplementary Figure 17. XPS spectra of Ir-NG. a, Survey spectrum of Ir-NG, revealing the absence of S (shadow area) and successful doping of N into graphene. **b,** High-resolution XPS N 1*s* spectrum, confirming the presence of Ir-N coordination in the pyridinic form. **c,** High-resolution XPS Ir 4*f* spectra. The corresponding binding energy of Ir for Ir-NG is between that for Ir-NSG and IrO_2 as highlighted by the dash line, in accordance with the medium electronegativity of N comparing to that of O and S.

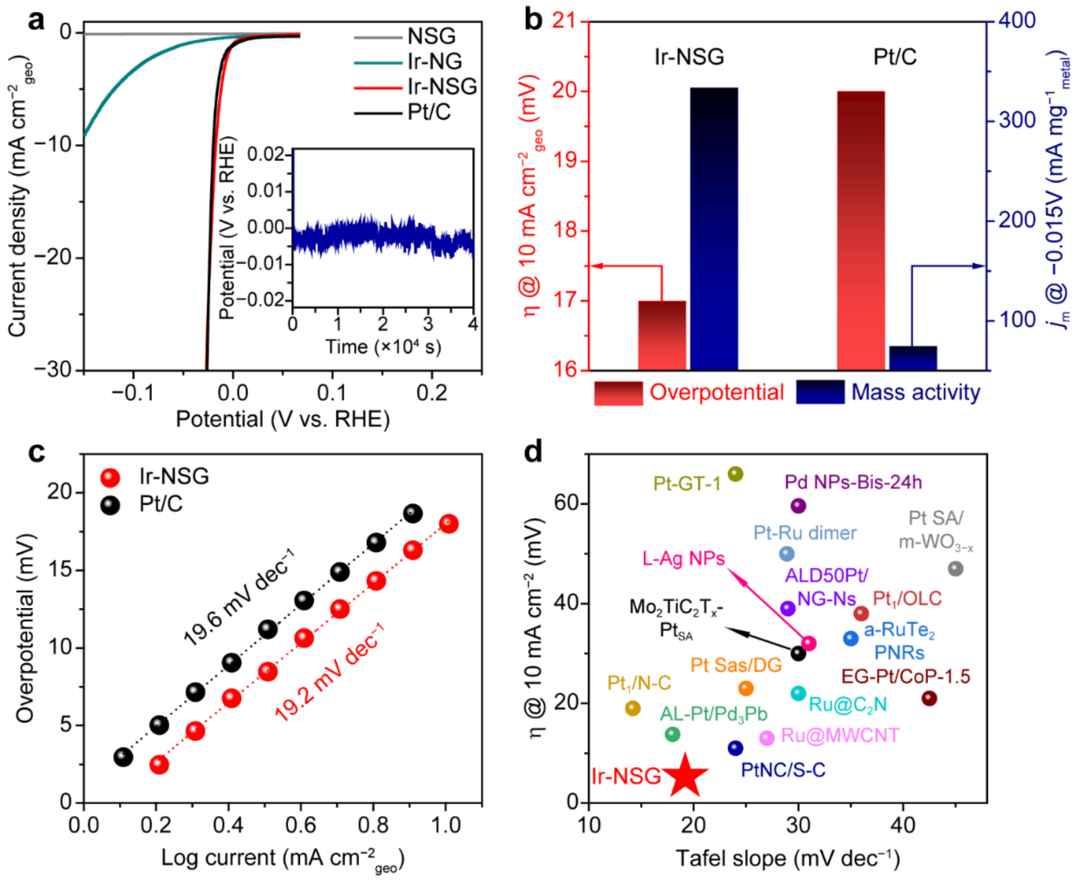

Supplementary Figure 18. HER performance of the Ir-NSG catalyst in acidic electrolyte. a, Polarization curves of Ir-NSG and commercial Pt/C acquired at a sweep rate of 1 mV s^{-1}. The inset shows chronopotentiometry of Ir-NSG. Catalyst loading: 0.3 mg cm^{-2}. Rotation speed: 1,600 rpm. **b,** The comparisons of overpotentials to drive a current density of 10 mA cm^{-2} and metal-mass specific activities at −0.015 V (vs. RHE) between Ir-NSG and commercial Pt/C. **c,** Corresponding Tafel plots for Ir-NSG and commercial Pt/C. All the measurements were operated in Ar-saturated 0.1 M $HClO_4$ with *iR*-compensation. **d,** Comparisons of overpotential (@ 10 mA cm^{-2}) and Tafel slope for various state-of-the-art noble metal-based HER catalysts in acidic medium. The overpotential of Ir-NSG was collected from LSV curves obtained at the scan rate of 5 mV s^{-1} and other values were plotted from references in Supplementary Table 2.

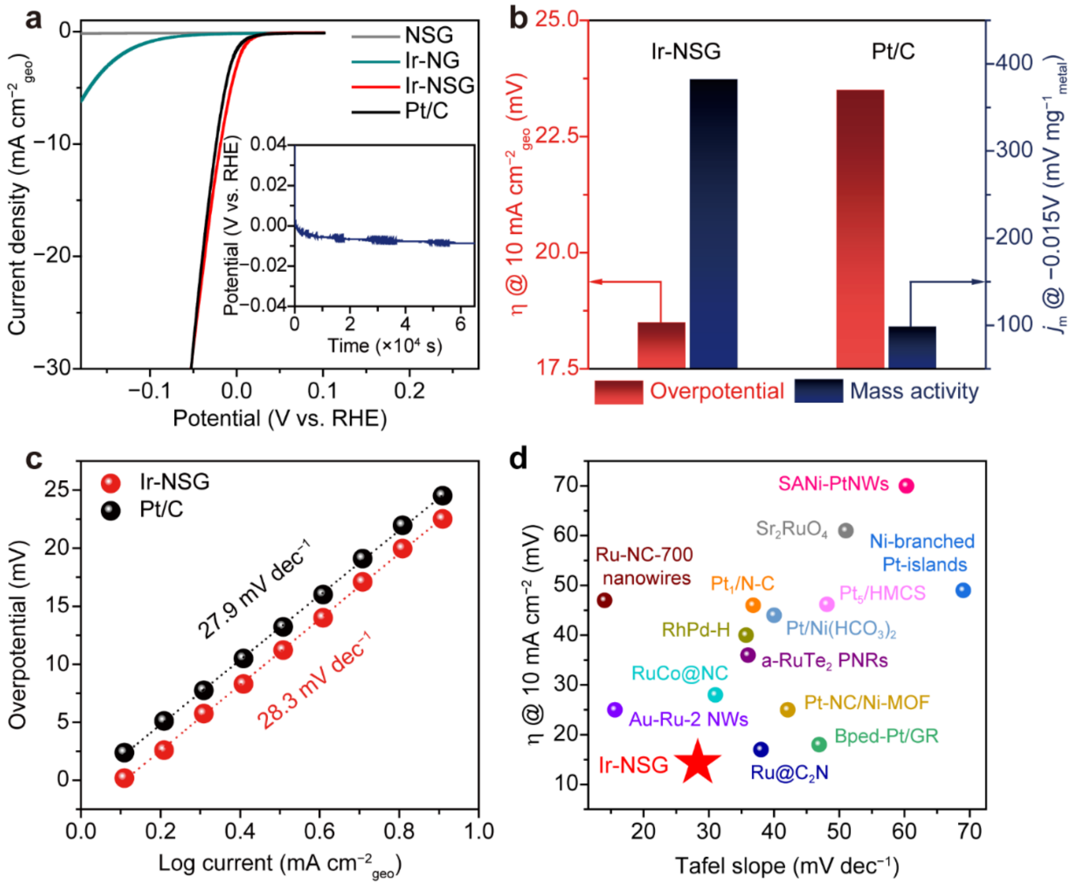

Supplementary Figure 19. HER performance of the Ir-NSG catalyst in alkaline electrolyte. a, Polarization curves of Ir-NSG and commercial Pt/C acquired at a sweep rate of 1 mV s^{-1}. The inset shows chronopotentiometry of Ir-NSG. Catalyst loading: 0.3 mg cm^{-2}. Rotation speed: 1,600 rpm. **b,** The comparisons of overpotentials to drive a current density of 10 mA cm^{-2} and metal-mass specific activities at −0.015 V (vs. RHE) between Ir-NSG and commercial Pt/C. **c,** Corresponding Tafel plots for Ir-NSG and commercial Pt/C. All the measurements were operated in Ar-saturated 1 M KOH with *iR*-compensation. **d,** Comparisons of overpotential (@ 10 mA cm^{-2}) and Tafel slope for various state-of-the-art noble metal-based HER catalysts in alkaline medium. The overpotential of Ir-NSG was collected from LSV curves obtained at the scan rate of 5 mV s^{-1} and other values were plotted from references in Supplementary Table 3.

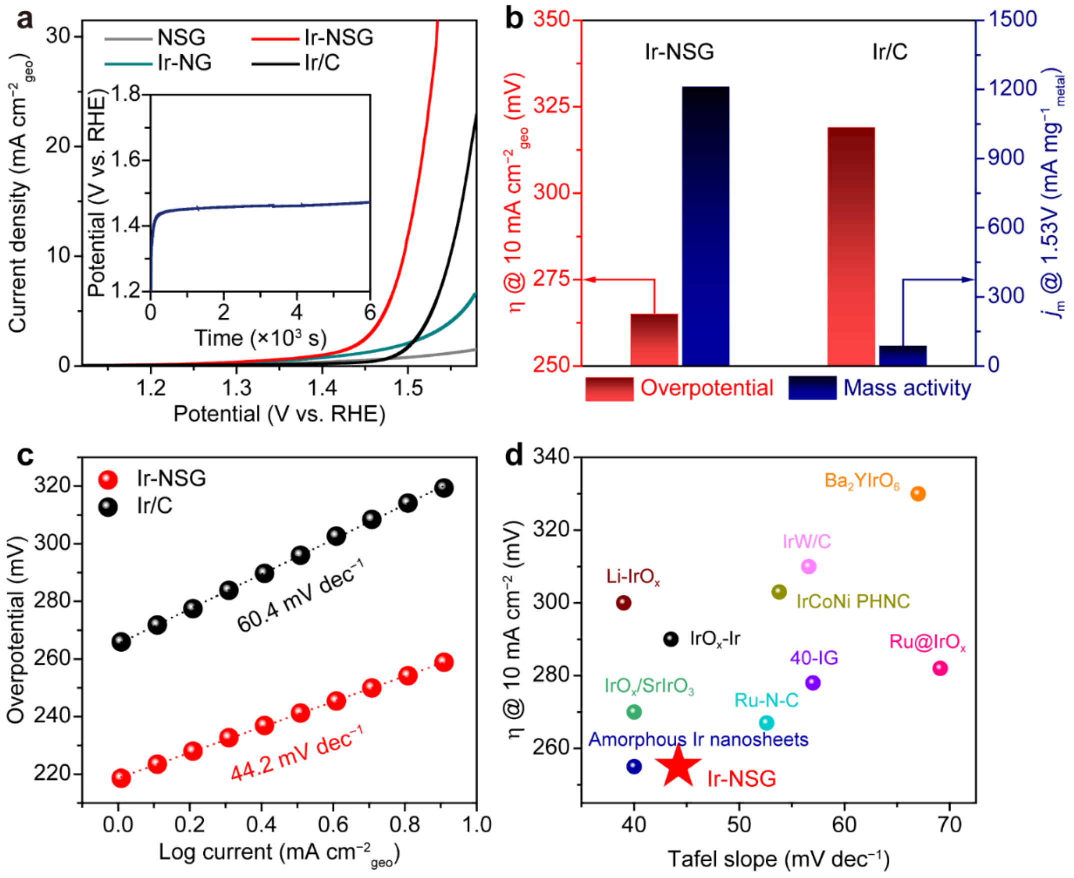

Supplementary Figure 20. OER performance of the Ir-NSG catalyst in acidic electrolyte. a, Polarization curves of Ir-NSG and commercial Ir/C acquired at a sweep rate of 1 mV s^{-1}, showing a lower onset potential for Ir-NSG than that for commercial Ir/C. The inset shows chronopotentiometry of Ir-NSG, the slight decay of current density is caused by the gradual detachment of catalyst from the RDE at a rotation speed of 1,600 rpm. Catalyst loading: 0.3 mg cm^{-2}. Rotation speed: 1,600 rpm. **b,** The comparisons of overpotentials to drive a current density of 10 mA cm^{-2} and metal-mass specific activities at 1.53 V (vs. RHE) between Ir-NSG and commercial Ir/C. Overpotential for Ir-NSG is 265 mV, better than that for commercial Ir/C (319 mV). Meanwhile, Ir-NSG obtains an Ir-mass specific activity of 1211.62 mA mg_{metal}^{-1}, which is 13.8-fold higher than that for commercial Ir/C (87.83 mA mg_{metal}^{-1}). **c,** The corresponding Tafel plots for Ir-NSG and commercial Ir/C. All the data were collected with *i*-*R* correction. **d,** Comparisons of overpotential (@ 10 mA cm^{-2}) and Tafel slope for various state-of-the-art noble metal-based OER catalysts in acidic medium. The overpotential of Ir-NSG was collected from LSV curves obtained at the scan rate of 5 mV s^{-1} and other values were plotted from references in Supplementary Table 5.

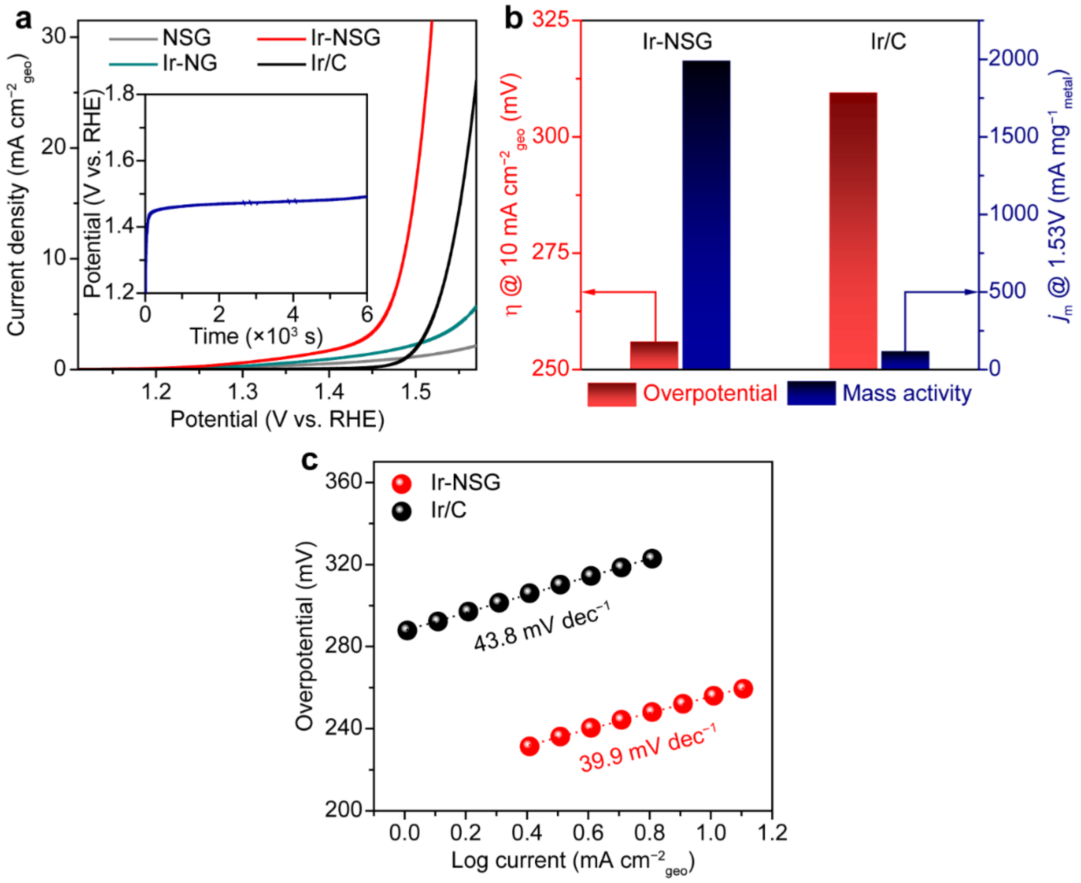

Supplementary Figure 21. OER performance of the Ir-NSG catalyst in alkaline electrolyte. a, Polarization curves of Ir-NSG and commercial Ir/C acquired at a sweep rate of 1 mV s^{-1}, showing a lower onset potential for Ir-NSG than that for commercial Ir/C. The inset shows chronopotentiometry of Ir-NSG. Catalyst loading: 0.3 mg cm^{-2}. Rotation speed: 1,600 rpm. **b,** The comparisons of overpotentials to drive a current density of 10 mA cm^{-2} and metal-mass specific activities at 1.53 V (vs. RHE) between Ir-NSG and commercial Ir/C. Overpotential for Ir-NSG is 256 mV, better than that for commercial Ir/C (309.5 mV). Meanwhile, Ir-NSG obtains an Ir-mass specific activity of 1989.92 mA mg_{metal}^{-1}, which is 16.9-fold higher than that for commercial Ir/C (117.58 mA mg_{metal}^{-1}). **c,** The corresponding Tafel plots for Ir-NSG and commercial Ir/C. All the data were collected with *i*-*R* correction.

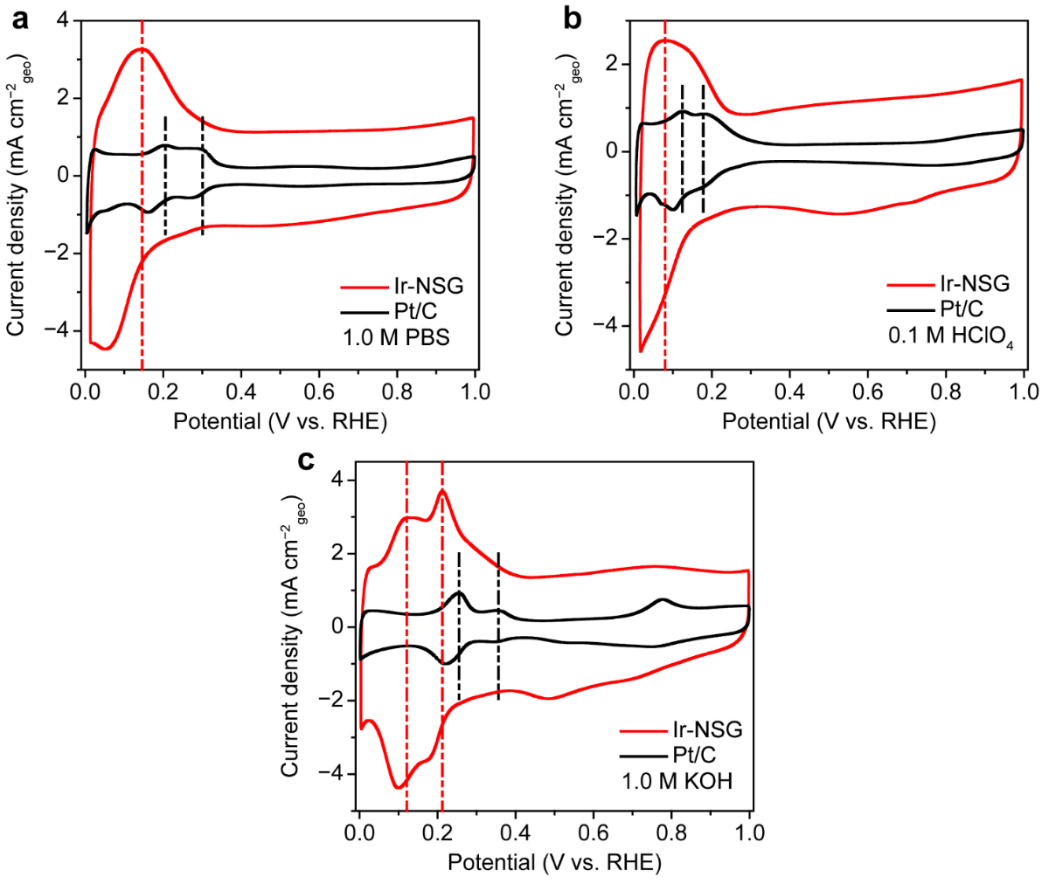

Supplementary Figure 22. Underpotentially deposited hydrogen of the Ir-NSG catalyst and commercial Pt/C. Steady-state CV curves of Ir-NSG and commercial Pt/C collected at a sweep rate of 50 mV s^{-1} in the potential range of 0.0 to 1.0 V (vs. RHE) in **a,** 1 M PBS. The HBEs are determined to be −0.146 eV for Ir-NSG, while −0.206 and −0.295 eV for Pt(110) and Pt(100), respectively. **b,** 0.1 M $HClO_4$. The HBEs are determined to be −0.080 eV for Ir-NSG, while −0.124 and −0.178 eV for Pt(110) and Pt(100), respectively. **c,** 1 M KOH. The HBEs are determined to be −0.121 and −0.212 eV for Ir(111) and Ir(200), while −0.255 and −0.356 eV for Pt(110) and Pt(100), respectively.[8] Additionally, peaks above ~0.6 V are related to OH^- anion adsorption/desorption.[9] Catalyst loading: 0.3 mg cm^{-2}. All the measurements were operated in Ar-saturated electrolytes with *iR*-compensation. The dashed lines serve as the eye guide for comparison. H_{upd} desorption region is chosen for analysis because the H_{upd} adsorption process is susceptible to the effect of pre-adsorbed species whereas the H_{upd} desorption process remains essentially stable.[8] The broad feature of the H_{upd} peaks for Ir-NSG could be interpreted as the existence of a variety of Ir sites.

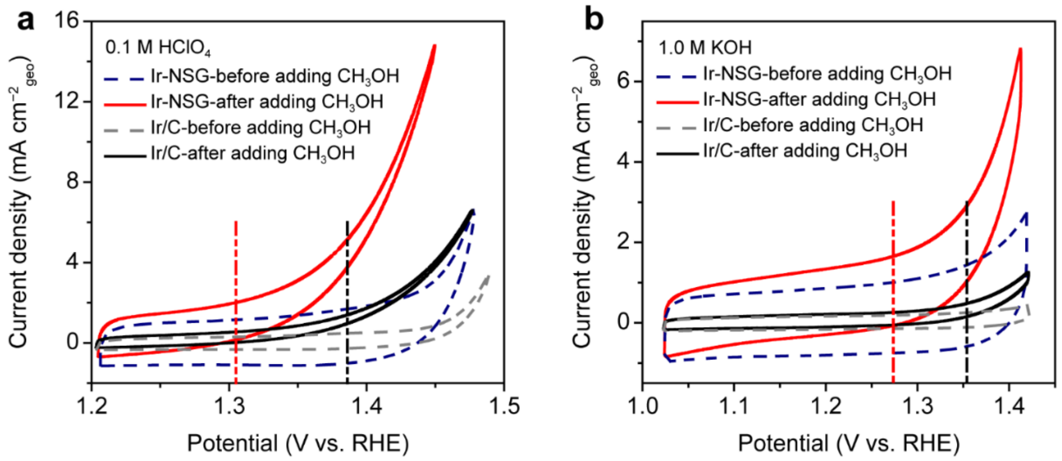

Supplementary Figure 23. Onset potential of methanol oxidation for the Ir-NSG catalyst and commercial Ir/C. Steady-state CV curves of Ir-NSG and commercial Ir/C with (solid line) and without (dotted line) 0.1 M methanol recorded at a sweep rate of 50 mV s^{-1} in **a,** 0.1 M $HClO_4$ and **b,** 1 M KOH. The dash-dot lines serve as the eye guide for the onset potential of methanol oxidation. All the data were collected with *i*-*R* correction.

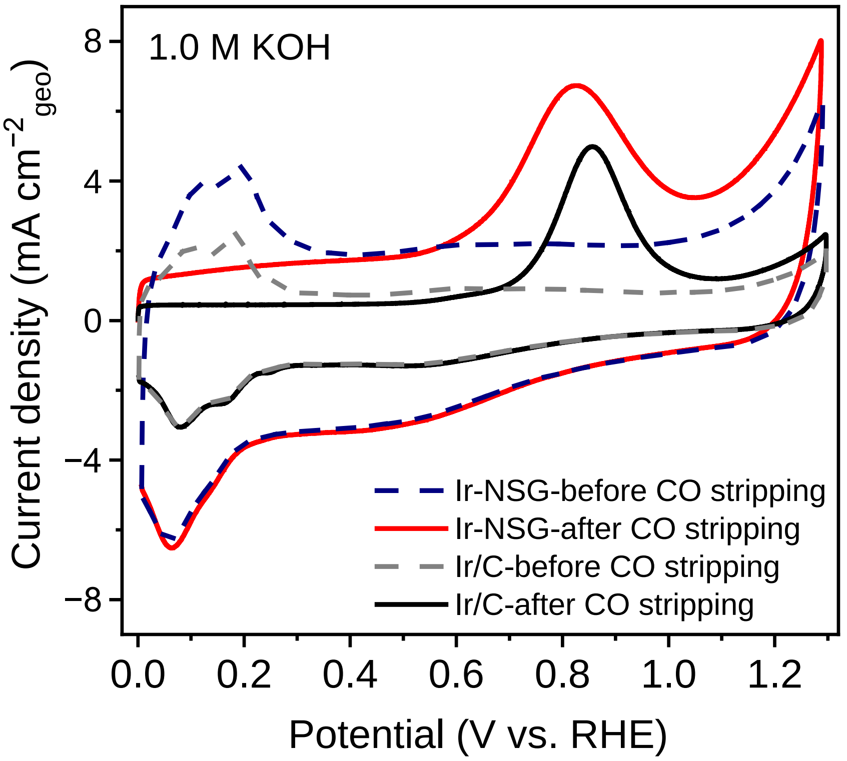

Supplementary Figure 24. CVs collected before (dotted line) and after (solid line) CO stripping of Ir-NSG and Ir/C, assuming that the electrooxidation of a CO* monolayer requires 420 μC cm^{-2}.[10] All the measurements were performed in 1 M KOH at a sweep rate of 50 mV s^{-1}. The CV curves have been corrected for solution resistance.

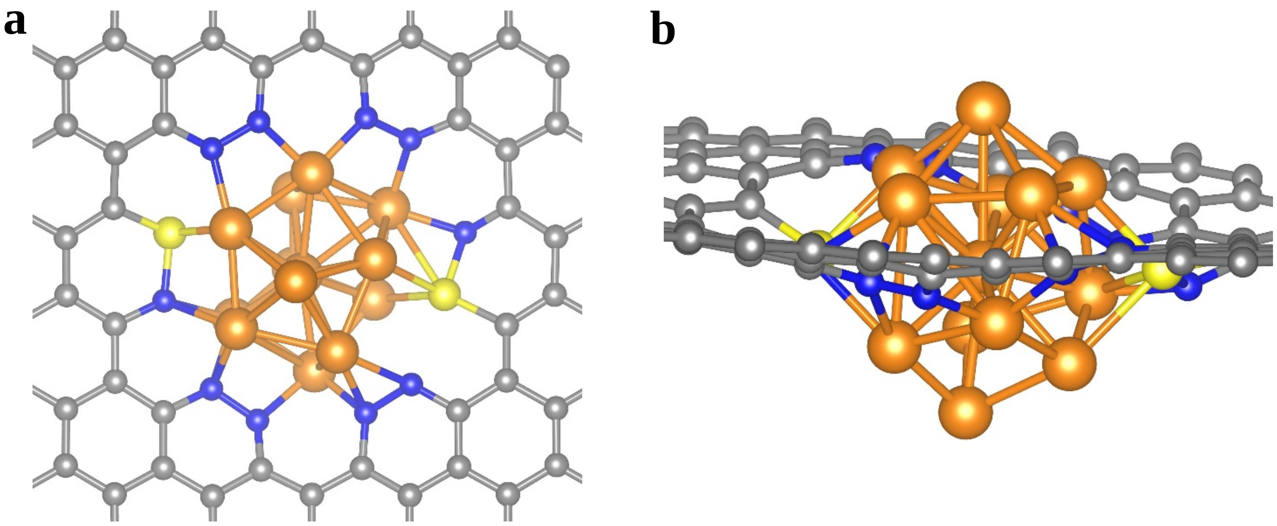

Supplementary Figure 25. Geometric structure of Ir_{13}@NSG model. a, top view. **b,** side view. The C atoms are in grey, N in blue, S in yellow and Ir in orange, respectively.

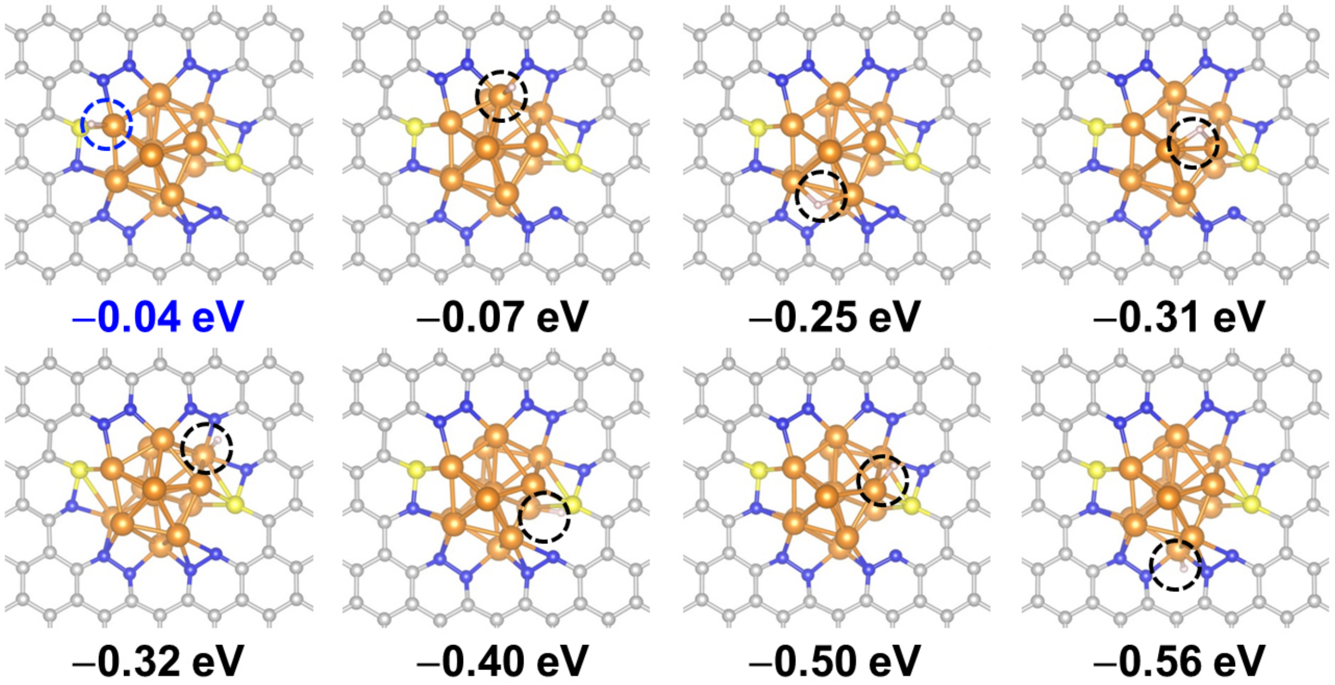

Supplementary Figure 26. Hydrogen adsorption configurations (H*) on atomic Ir sites and the corresponding Gibbs free energies (ΔG_{H*}) of Ir_{13}@NSG. All the possibilities of H* structures are considered while those with $|\Delta G_{H*}| > 0.56$ eV are not shown. The C atoms are in grey, N in blue, S in yellow and Ir in orange, respectively.

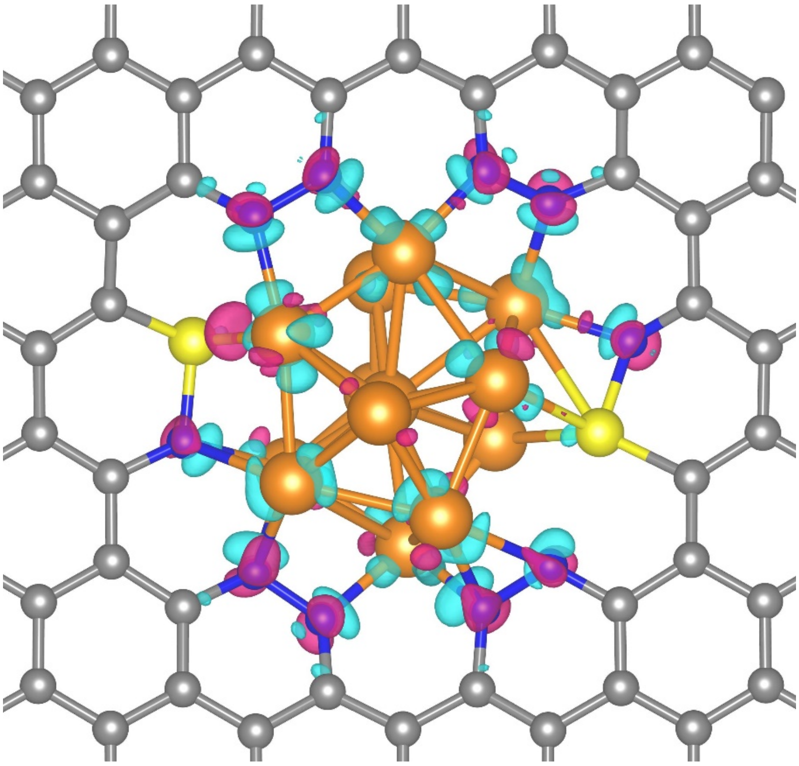

Supplementary Figure 27. The isosurface of the charge density difference for Ir_{13}@NSG. The C atoms are in grey, N in blue, S in yellow and Ir in orange, respectively. The purple and cyan regions refer to electron accumulation and depletion, respectively.

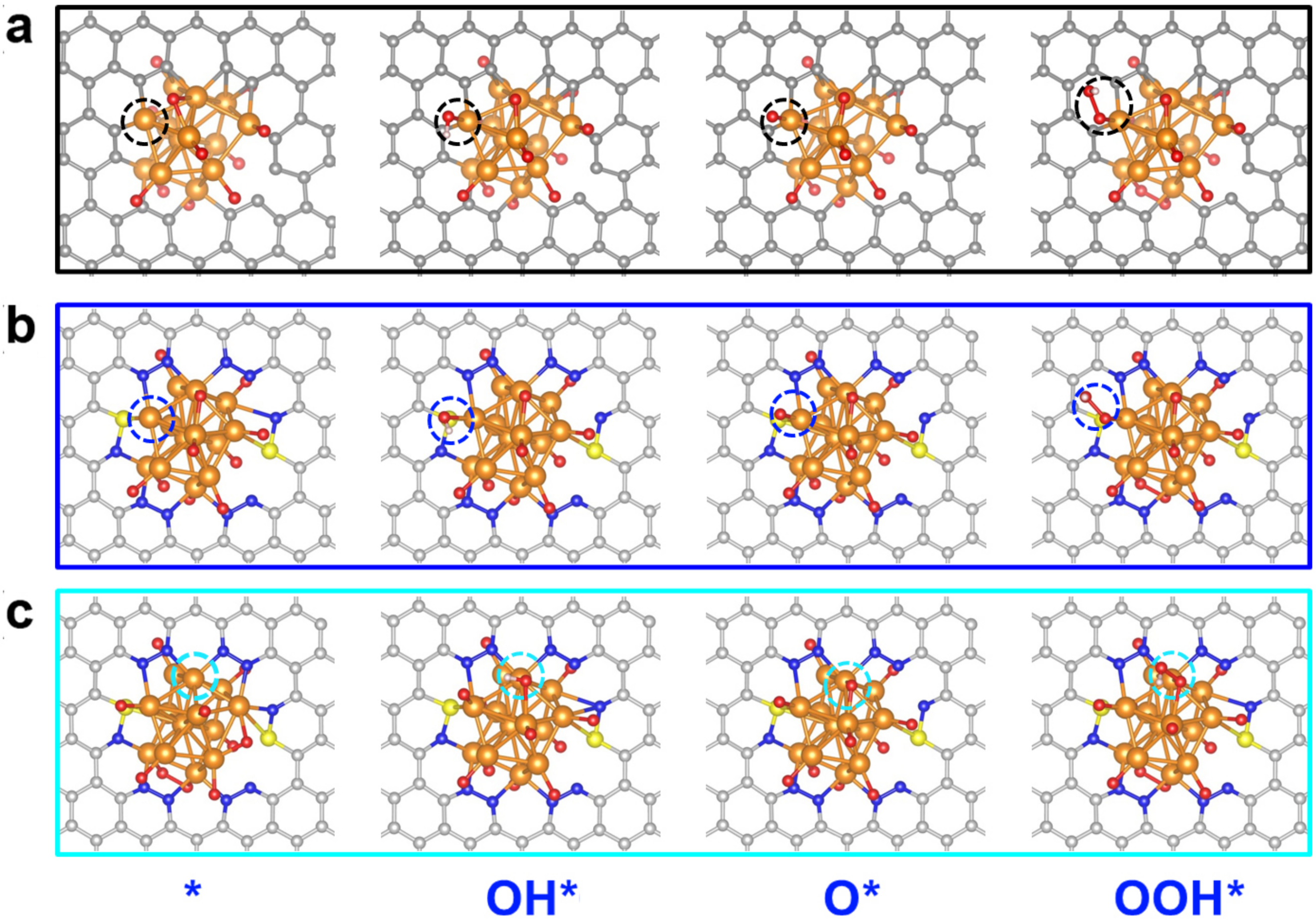

Supplementary Figure 28. Schematic of the OER mechanism and surface configurations on different active sites. a, Ir atom coordinated with C in $Ir_{13}O_{11}$@G (Ir_C*). **b,** Ir atom coordinated with N and S in $Ir_{13}O_{11}$@NSG (Ir_S*). **c,** Ir atom coordinated with N in $Ir_{13}O_{11}$@NSG (Ir_N*). The C atoms are in grey, N in blue, S in yellow, O in red, and Ir in gold, respectively.

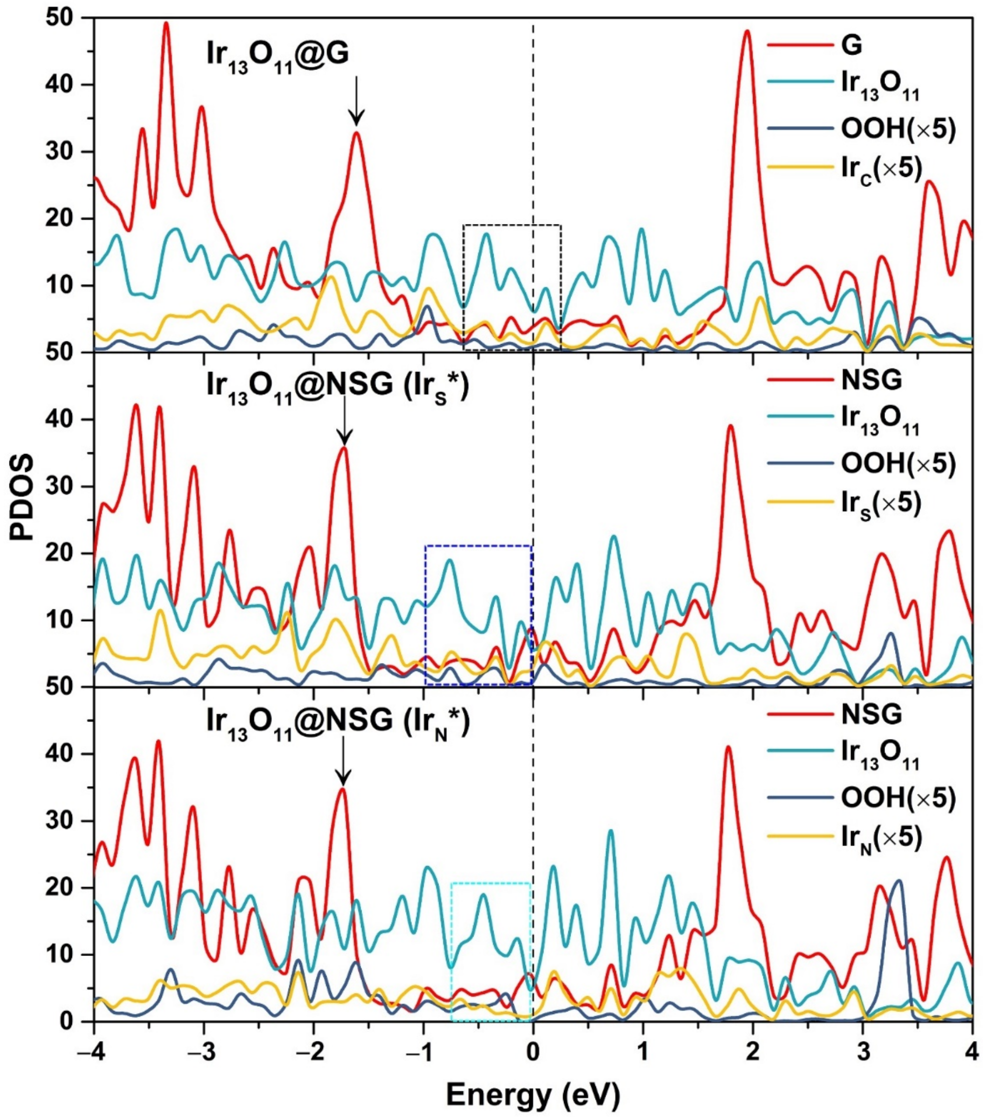

Supplementary Figure 29. The projected density of states (PDOS) distributions of OOH* for $Ir_{13}O_{11}$@G, $Ir_{13}O_{11}$@NSG with N,S-coordinated Ir active site (Ir_S*) and N-coordinated Ir active site (Ir_N*). The Fermi level is set to zero, as shown by the dashed line. The PDOS for OOH and Ir active sites are enlarged by five times for clarity.

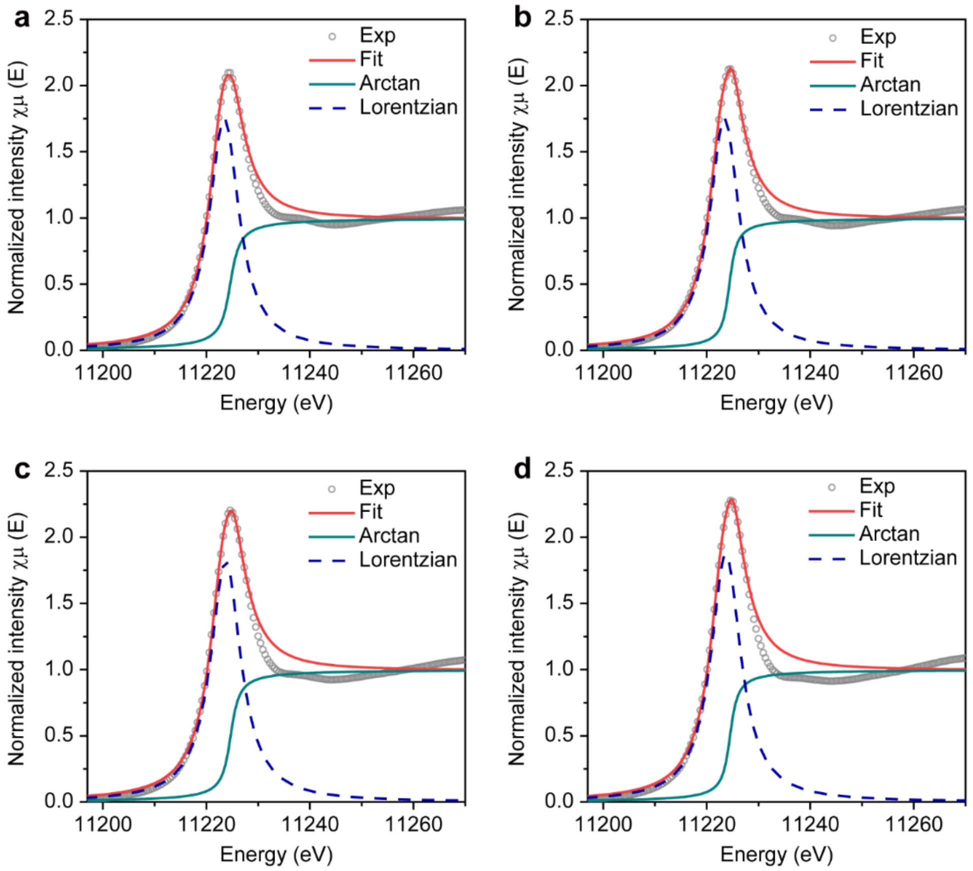

Supplementary Figure 30. Fitting of operando XANES spectra for Ir-NSG at various biases using a set of arctangent function (green solid line) and Lorentzian function (blue dashed line). The gray dots and red solid lines represent the experimental data and fitting curves, respectively. Conditions are **a,** open-circuit voltage (OCV). **b,** 1.25 V vs. RHE. **c,** 1.4 V vs. RHE. **d,** 1.55 V vs. RHE. The arctangent function is applied to model the continuum step at the L_3 absorption edge, which is centered at the absorption edge with a fixed unit amplitude in all the fits.[6]

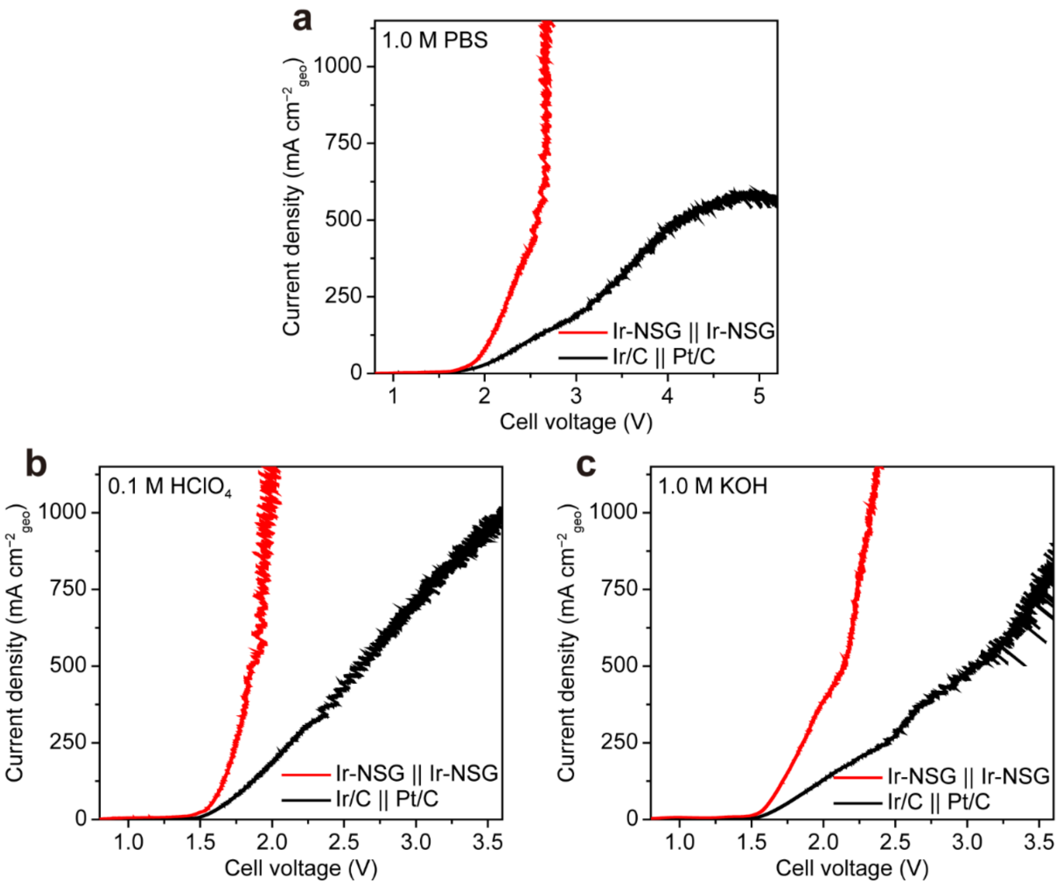

Supplementary Figure 31. Overall-water-splitting performance. LSV curves of Ir-NSG and Ir/C-Pt/C coupled water electrolysis cell at a scan rate of 10 mV s^{-1} in **a,** 1 M PBS, **b,** 0.1 M $HClO_4$, and **c,** 1 M KOH. Catalyst loading: 73.3 μg_{metal} cm^{-2}. All measurements were conducted in Ar-saturated electrolytes and corrected for 85% *i*-*R* drop.

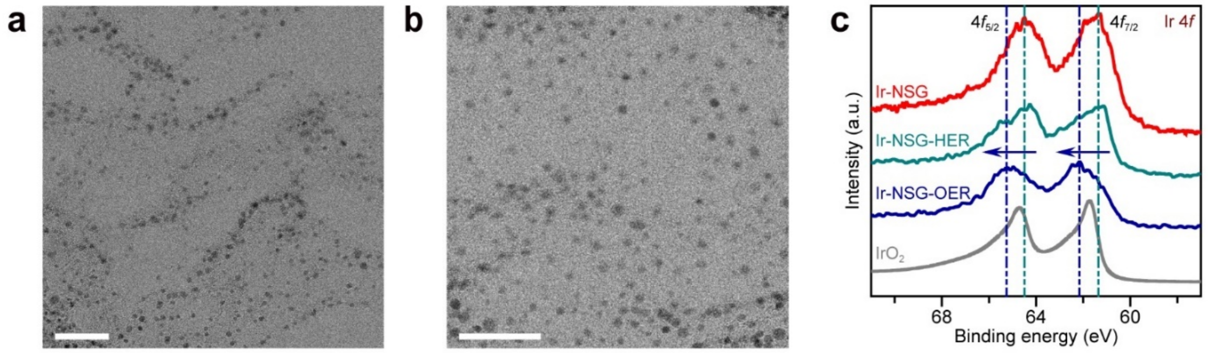

Supplementary Figure 32. Structure of Ir-NSG after long-term stability test. a, Bright-field TEM image of Ir-NSG after HER test, scale bar: 20 nm. **b,** Bright-field TEM image of Ir-NSG after OER test, scale bar: 20 nm. As shown in **a** and **b**, the Ir-NSG catalyst keeps uniformly dispersed nanoclusters without aggregation after undergoing long-term HER and OER. **c,** High-resolution XPS Ir 4*f* spectra. The corresponding binding energy of Ir highlighted by the dash line reveals that the valence state of Ir keeps almost constant during HER (green dash line) while becoming higher than that of IrO_2 after OER (blue dash line), which is in accordance with the results of *in-situ* XAFS measurements.

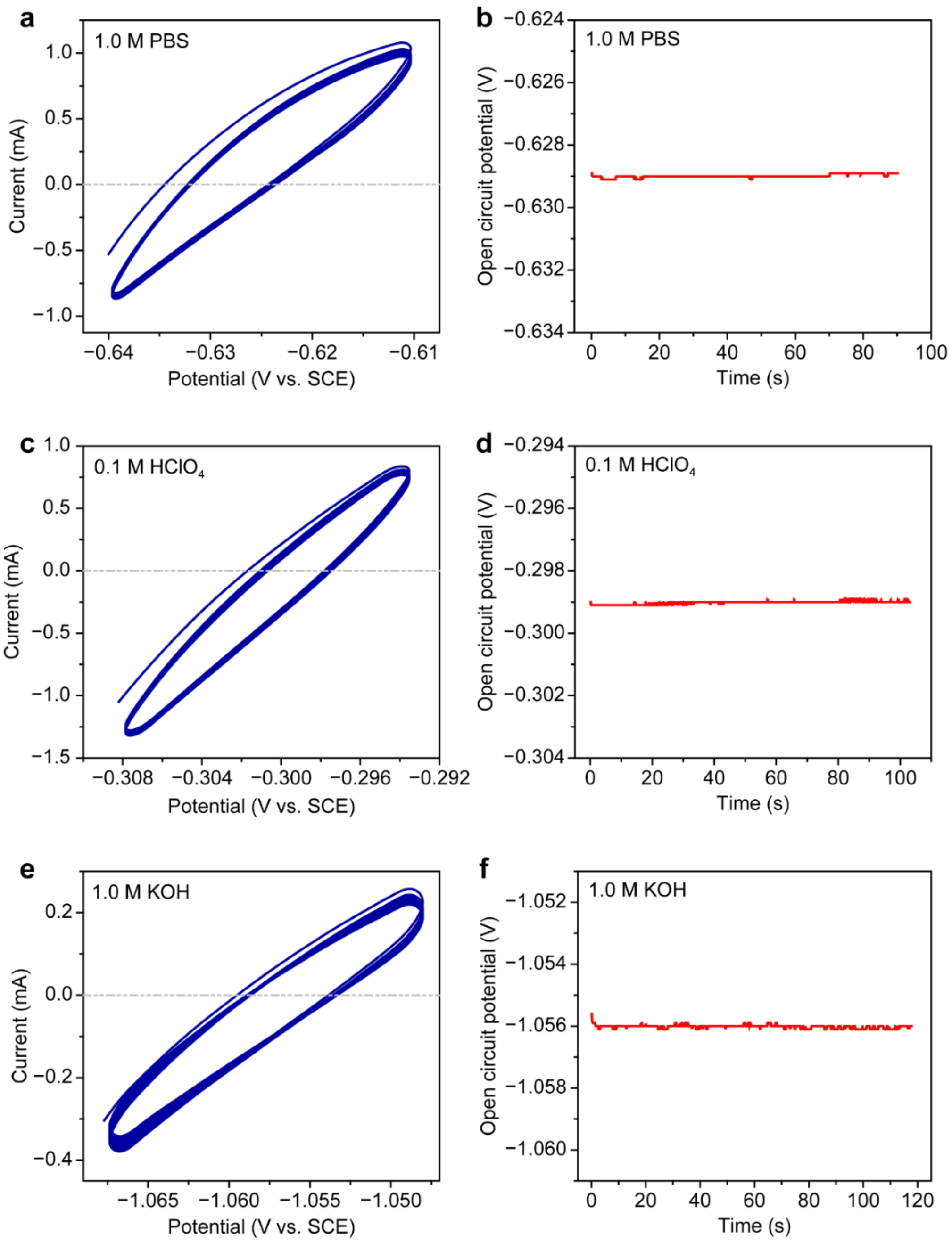

Supplementary Figure 33. Calibration of the saturated calomel reference electrode (SCE) with saturated KCl salt bridge by RHE. a, c, e, Cyclic voltammograms of Pt electrode at a scan rate of 10 mV s^{-1} in H_2 saturated 1 M PBS (**a**), 0.1 M $HClO_4$ (**c**) and 1 M KOH (**e**) solutions. **b, d, f,** Open circuit potentials of Pt electrode in H_2 saturated 1 M PBS (**b**), 0.1 M $HClO_4$ (**d**) and 1 M KOH (**f**) electrolytes along with time.

Supplementary Table 1. Comparison of HER activities with various recently reported state-of-the-art catalysts in neutral electrolyte.

Catalyst	Medium and electrode	Scan rate ($mV\ s^{-1}$)	η @ 10 mA cm^{-2} (mV)	Tafel slope ($mV\ dec^{-1}$)	Reference
Ir-NSG	1 M PBS; glassy carbon	1	22	21.2	This work
		5	16.8		
CrO_x/Cu-Ni	1 M PBS; 3D CuO nanowire scaffold	N/A	48	64	*Nat. Energy* **4**, 107-114 (2019)
CoP/Co-MOF	1 M PBS; carbon fiber paper	1	49	63	*Angew. Chem. Int. Ed.* **58**, 4679-4684 (2019)
Li_xNiO/Ni	1 M PBS; glassy carbon	5	50	66	*J. Am. Chem. Soc.* DOI: 10.1021/jacs.0c00241 (2020)
RuP_2@NPC	1 M PBS; glassy carbon	5	57	87	*Angew. Chem. Int. Ed.* **129**, 11717-11722 (2017)
$NiCo_2P_x$	1 M PBS; carbon felt	5	63	63.3	*Adv. Mater.* **29**, 1605502 (2017)
N-Ni	1 M PBS; Ni foam	N/A	64	106	*J. Am. Chem. Soc.* **139**, 12283-12290 (2017)
Ni/WC@NC	1 M PBS; N/A	5	73	80.9	*Energy Environ. Sci.* **11**, 2114-2123 (2018)
B-CoP/CNT	1 M PBS; glassy carbon	2	79	80	*Angew. Chem. Int. Ed.* **59**, 4154-4160 (2020)
Mn-NiO-Ni	1 M PBS; Ni foam	1	80	121	*Energy Environ. Sci.* **11**, 1898-1910 (2018)
PdP_2@CB	1 M PBS; glassy carbon	5	84.6	72.3	*Angew. Chem. Int. Ed.* **57**, 14862-14867 (2018)
Ni-C-N NSs	1 M PBS; glassy carbon	2	92.1	38	*J. Am. Chem. Soc.* **138**, 14546-14549 (2016)
Ru@CN-0.16	1 M PBS; glassy carbon	5	ca. 100	N/A	*Energy Environ. Sci.* **11**, 800-806 (2018)
Karst NF	1 M PBS; Ni foam	5	110	99	*Energy Environ. Sci.* **13**, 174-182 (2020)
CoMoNiS-NF-31	1 M PBS; Ni foam	5	117	56	*J. Am. Chem. Soc.* **141**, 10417-10430 (2019)
NFP/C-3	1 M PBS; carbon fiber paper	5	117	70	*Sci. Adv.* **5**, eaav6009 (2019)
Li-$IrSe_2$	1 M PBS; glassy carbon	2	120	N/A	*Angew. Chem. Int. Ed.* **58**, 14764-14769 (2019)
$Ni_{0.1}Co_{0.9}P$	1 M PBS; carbon fiber paper	5	125	103	*Angew. Chem. Int. Ed.* **130**, 15671-15675 (2018)
MHCF-CNTs@PEMAc	1 M PBS; glassy carbon	5	278	108	*Angew. Chem. Int. Ed.* **58**, 16217-16222 (2019)

Supplementary Table 2. Comparison of HER activities with various recently reported state-of-the-art noble metal-based catalysts in acidic electrolyte.

Catalyst	Medium and electrode	Scan rate (mV s^{-1})	η @ 10 mA cm^{-2} (mV)	Tafel slope (mV dec^{-1})	Reference
Ir-NSG	0.1 M $HClO_4$; glassy carbon	1	17	19.2	This work
		5	5.3		
PtNC/S-C	0.5 M H_2SO_4; glassy carbon	2	11	24	*Nat. Commun.* **10**, 4977 (2020)
Ru@MWCNT	0.5 M H_2SO_4; glassy carbon	5	13	27	*Nat. Commun.* **11**, 1278 (2020)
AL-Pt/Pd_3Pb	0.5 M H_2SO_4; glassy carbon	N/A	13.8	18	*J. Am. Chem. Soc.* **141**, 19964-19968 (2019)
Pt-GT-1	0.5 M H_2SO_4; glassy carbon	5	15	N/A	*Nat. Energy* **3**, 773-782 (2018)
Pt_1/hNCNC-2.92	0.5 M H_2SO_4; glassy carbon	5	15	24	*Nat. Commun.* **10**, 1657 (2019)
Pt_1/N-C	0.5 M H_2SO_4; glassy carbon	5	19	14.2	*Nat. Commun.* **11**, 1029 (2020)
RuCu NSs	0.5 M H_2SO_4; glassy carbon	5	19	26	*Angew. Chem. Int. Ed.* **58**, 13983-13988 (2019)
Pt_5/HMCS	0.5 M H_2SO_4; glassy carbon	1	20.7	28.3	*Adv. Mater.* **32**, 1901349 (2020)
EG-Pt/CoP-1.5	0.5 M H_2SO_4; carbon fiber paper	5	21	42.5	*Energy Environ. Sci.* **12**, 2298-2304 (2019)
PtW_6O_{24}/C	0.5 M H_2SO_4; glassy carbon	5	22	29.8	*Nat. Commun.* **11**, 490 (2020)
Ru@C_2N	0.5 M H_2SO_4; glassy carbon	5	22	30	*Nat. Nanotechnol.* **12**, 441-446 (2017)
Pt Sas/DG	0.5 M H_2SO_4; carbon fiber paper	5	23	25	*J. Am. Chem. Soc.* **141**, 4505-4509 (2019)
A-CoPt-NC	0.5 M H_2SO_4; glassy carbon	5	27	31	*Angew. Chem. Int. Ed.* **58**, 9404-9408 (2019)
Pt-AC/DG-3	0.1 M $HclO_4$; glassy carbon	5	29	34.8	*J. Am. Chem. Soc.* **142**, 5594-5601 (2020)
$Mo_2TiC_2T_x$-Pt_{SA}	0.5 M H_2SO_4; carbon paper	5	30	30	*Nat. Catal.* **1**, 985-992 (2018)
L-Ag NPs	0.5 M H_2SO_4; glassy carbon	5	32	31	*Nat. Catal.* **2**, 1107-1114 (2019)
a-$RuTe_2$ PNRs	0.5 M H_2SO_4; glassy carbon	N/A	33	35	*Nat. Commun.* **10**, 5692 (2019)
Pt_1/OLC	0.5 M H_2SO_4; glassy carbon	5	ca. 38	36	*Nat. Energy* **4**, 512-518 (2019)
ALD50Pt/NG-Ns	0.5 M H_2SO_4; glassy carbon	2	ca. 39	29	*Nat. Commun.* **7**, 13638 (2016)
Pt SA/m-WO_{3-x}	0.5 M H_2SO_4; glassy carbon	5	47	45	*Angew. Chem. Int. Ed.* **58**, 16038-16042 (2019)

Pt-Ru dimer	0.5 M H_2SO_4; glassy carbon	2	50	28.9	*Nat. Commun.* **10**, 4936 (2019)
Pd NPs-Bis-24h	0.5 M H_2SO_4; glassy carbon	5	59.6	30	*Adv. Mater.* **32**, 1902964 (2020)
Pt-GT-1	0.1 M $HclO_4$; glassy carbon	2	66	24	*Nat. Energy* **3**, 773-782 (2018)
IrNiTa/Si	0.5 M H_2SO_4; Ti	2	99	35	*Adv. Mater.* **32**, 1906384 (2020)

Supplementary Table 3. Comparison of HER activities with various recently reported state-of-the-art noble metal-based catalysts in alkaline electrolyte.

Catalyst	Medium and electrode	Scan rate (mV s^{-1})	η @ 10 mA cm^{-2} (mV)	Tafel slope (mV dec^{-1})	Reference
Ir-NSG	1 M KOH; glassy carbon	1	18.5	28.3	This work
		5	14.3		
Ru@MWCNT	1 M KOH; glassy carbon	5	17	27	*Nat. Commun.* **11**, 1278 (2020)
Ru@C_2N	1 M KOH; glassy carbon	5	17	38	*Nat. Nanotechnol.* **12**, 441-446 (2017)
Bped-Pt/GR	1 M KOH; glassy carbon	5	18	46.9	*Angew. Chem. Int. Ed.* **58**, 19060-19066 (2019)
Au-Ru-2 NWs	1 M KOH; glassy carbon	2	25	15.6	*Nat. Chem.* **10**, 456–461 (2018)
Pt-NC/Ni-MOF	1 M KOH; glassy carbon	5	25	42.1	*Chem* **5**, 2429-2441 (2019)
RuCo@NC	1 M KOH; glassy carbon	2	28	31	*Nat. Commun.* **8**, 14969 (2017)
a-$RuTe_2$ PNRs	1 M KOH; glassy carbon	N/A	36	36	*Nat. Commun.* **10**, 5692 (2019)
PtNi-O/C	1 M KOH; glassy carbon	5	39.8	78.8	*J. Am. Chem. Soc.* **140**, 9046-9050 (2018)
RhPd-H	1 M KOH; glassy carbon	5	40	35.7	*J. Am. Chem. Soc.* **142**, 3645-3651 (2020)
Pt_3Ni_2-NWs-S/C	1 M KOH; glassy carbon	10	42	N/A	*Nat. Commun.* **8**, 14580 (2017)
Pt/$Ni(HCO_3)_2$	1 M KOH; glassy carbon	5	44	40	*Angew. Chem. Int. Ed.* **58**, 5432-5437 (2019)
Pt_1/N-C	1 M KOH; glassy carbon	5	46	36.8	*Nat. Commun.* **11**, 1029 (2020)
Pt_5/HMCS	1 M KOH; glassy carbon	1	46.2	48.1	*Adv. Mater.* **32**, 1901349 (2020)
Ru-NC-700 nanowires	0.1 M KOH; glassy carbon	N/A	47	14	*Nat. Commun.* **10**, 631 (2019)
Ni-branched Pt-islands	0.1 M KOH; glassy carbon	10	49	69	*J. Am. Chem. Soc.* **141**, 16202-16207 (2019)
Ir_1@Co/NC	1 M KOH; glassy carbon	10	55	119	*Angew. Chem. Int. Ed.* **58**, 11868-11873 (2019)
Sr_2RuO_4	1 M KOH; glassy carbon	5	61	51	*Nat. Commun.* **10**, 149 (2019)
hcp Pt-Ni nano-multipods	0.1 M KOH; glassy carbon	10	65	78	*Nat. Commun.* **8**, 15131 (2017)
SANi-PtNWs	1 M KOH; glassy carbon	5	ca. 70	60.3	*Nat. Catal.* **2**, 495-503 (2019)

Supplementary Table 4. Comparison of OER activities with various recently reported state-of-the-art catalysts in neutral electrolyte.

Catalyst	Medium and electrode	Scan rate (mV s^{-1})	η @ 10 mA cm^{-2} (mV)	Tafel slope (mV dec^{-1})	Reference
Ir-NSG	1 M PBS; glassy carbon	1	307	74.2	This work
		5	297		
Li-$IrSe_2$	1 M PBS; carbon fiber paper	2	315	N/A	*Angew. Chem. Int. Ed.* **58**, 14764-14769 (2019)
NiCoFeP	0.5 M $KHCO_3$; Au-plated Ni foam	1	330	60	*Nat. Chem.* **10**, 149–154 (2017)
$Co_3(PO4)_2$ nanosheets	0.1 M PBS; glassy carbon	5	360	70	*Angew. Chem. Int. Ed.* **58**, 14599-14604 (2019)
Ni-Fe-Mg	0.5 M $KHCO_3$; Ni foam	1	360	150	*Adv. Mater.* **32**, 1906806 (2020)
CoIr-0.2	1 M PBS; glassy carbon	10	373	117.5	*Adv. Mater.* **30**, 1707522 (2018)
Co-Pi NA/Ti	1 M PBS; Ti mesh	5	380	187	*Angew. Chem. Int. Ed.* **56**, 1064-1068 (2017)
CoMoNiS-NF-31	1 M PBS; Ni foam	5	405	71	*J. Am. Chem. Soc.* **141**, 10417-10430 (2019)
$NiFe_2O_4/FeNi_2S_4$ HNSs	0.2 M PBS; carbon fiber paper	2	429	N/A	*J. Am. Chem. Soc.* **140**, 17624-17631 (2018)
Karst NF	1 M PBS; Ni foam	5	432	249	*Energy Environ. Sci.* **13**, 174-182 (2020)
IrO_2	0.5 M $NaHCO_3$; Ti foil	10	460	N/A	*Nat. Commun.* **6**, 7326 (2015)
Co4Mo	0.1 M PBS; glassy carbon	10	490	144	*Angew. Chem. Int. Ed.* **131**, 145-149 (2019)
Co-Bi NS/G	1 M PBS; glassy carbon	5	ca. 500	160	*Angew. Chem. Int. Ed.* **55**, 2488-2492 (2016)
**1**@Fe_3O_4 NR	0.1 M PBS; Ti mesh	5	510	61.4	*Angew. Chem. Int. Ed.* **58**, 18883-18887 (2019)
$Ni_{0.1}Co_{0.9}P$	1 M PBS; carbon fiber paper	5	550	133	*Angew. Chem. Int. Ed.* **130**, 15671-15675 (2018)
LT-$LiCoO_2$	1 M PBS; glassy carbon	5	570	75	*Energy Environ. Sci.* **9**, 184-192 (2016)

Supplementary Table 5. Comparison of OER activities with various recently reported state-of-the-art noble metal-based catalysts in acidic electrolyte.

Catalyst	Medium and electrode	Scan rate (mV s^{-1})	η @ 10 mA cm^{-2} (mV)	Tafel slope (mV dec^{-1})	Reference
Ir-NSG	0.1 M $HClO_4$; glassy carbon	1	265	44.2	This work
		5	255		
$CaCu_3Ru_4O_{12}$	0.5 M H_2SO_4; glassy carbon	5	171	40	*Nat. Commun.* **10**, 3809 (2019)
Ru_1-Pt_3Cu	0.1 M $HClO_4$; glassy carbon	N/A	ca. 220	N/A	*Nat. Catal.* **2**, 304-313 (2019)
Amorphous Ir nanosheets	0.1 M $HClO_4$; glassy carbon	5	255	40	*Nat. Commun.* **10**, 4855 (2019)
Ru-N-C	0.5 M H_2SO_4; glassy carbon	5	267	52.6	*Nat. Commun.* **10**, 4849 (2019)
$IrO_x/SrIrO_3$	0.5 M H_2SO_4; $SrIrO_3$ film	10	270	40	*Science* **353**, 1011-1014 (2016)
40-IG	0.5 M H_2SO_4; glassy carbon	5	278	57	*Angew. Chem. Int. Ed.* **58**, 12540-12544 (2019)
Ru@IrO_x	0.05 M H_2SO_4; gold electrode	5	282	69.1	*Chem* **5**, 445-459 (2019)
$Pr_2Ir_2O_7$	0.1 M $HClO_4$; glassy carbon	5	290	N/A	*Adv. Mater.* **31**, 1805104 (2019)
IrO_x-Ir	0.5 M H_2SO_4; glassy carbon	5	290	43.5	*Angew. Chem. Int. Ed.* **128**, 752-756 (2016)
Dtf-IrOs	0.1 M $HClO_4$; glassy carbon	10	ca. 290	N/A	*Nat. Commun.* **8**, 1449 (2017)
Li-IrO_x	0.5 M H_2SO_4; glassy carbon	1	300	39	*J. Am. Chem. Soc.* **141**, 3014-3023 (2019)
IrCoNi PHNC	0.1 M $HClO_4$; glassy carbon	5	303	53.8	*Adv. Mater.* **29**, 1703798 (2017)
IrW/C	0.1 M $HClO_4$; glassy carbon	5	ca. 310	56.6	*ACS Cent. Sci.* **4**, 1244-1252 (2018)
Ba_2YIrO_6	0.1 M $HClO_4$; gold electrode	10	ca. 330	67	*Nat. Commun.* **7**, 12363 (2016)
N-HC@G-900	0.5 M H_2SO_4; glassy carbon	5	350	88.1	*Angew. Chem. Int. Ed.* **130**, 16749-16753 (2018)
$W_{0.57}Ir_{0.43}O_{3-\sigma}$	1 M H_2SO_4; glassy carbon	20	370	125	*Energy Environ. Sci.* **10**, 2432-2440 (2017)
Sr_2CoIrO_6	0.1 M $HClO_4$; glassy carbon	10	ca. 370	N/A	*Angew. Chem. Int. Ed.* **131**, 4619-4623 (2019)
Rh_2P/C	0.5 M H_2SO_4; glassy carbon	20	550	N/A	*J. Am. Chem. Soc.* **139**, 5494-5502 (2017)

Supplementary Table 6. Comparison of overall-water-splitting activities and stabilities with various recently reported state-of-the-art electrocatalysts in acidic electrolyte.

Catalyst	Medium and electrode	Cell Voltage (V)	Durability @ 10 mA cm^{-2} (h)	Reference
Ir-NSG	0.1 M $HClO_4$; carbon fiber paper	1.42	24 h @ 10 mA cm^{-2}	This work
Li-$IrSe_2$	0.5 M H_2SO_4; carbon fiber paper	1.44	24 h @ 1.47 V	*Angew. Chem. Int. Ed.* **58**, 14764-14769 (2019)
CoMoNiS-NF-31	0.5 M H_2SO_4; carbon fiber paper	1.45	80 min @ 1.53 V	*J. Am. Chem. Soc.* **141**, 10417-10430 (2019)
IrW/C	0.5 M H_2SO_4; carbon fiber paper	1.45	8 h @ 10 mA cm^{-2}	*ACS Cent. Sci.* **4**, 1244-1252 (2018)
$RuIrO_x$	0.5 M H_2SO_4; carbon fiber paper	1.45	24 h @ 10 mA cm^{-2}	*Nat. Commun.* **10**, 4875 (2019)
RuCu NCs	0.5 M H_2SO_4; N/A	1.49	15 h @ 10 mA cm^{-2}	*Angew. Chem. Int. Ed.* **58**, 13983-13988 (2019)
Co-RuIr	0.1 M $HClO_4$; N/A	1.52	25 h @ 10 mA cm^{-2}	*Adv. Mater.* **31**, 1900510 (2019)
a-$RuTe_2$ PNRs	0.5 M H_2SO_4; N/A	1.52	24 h @ 7 mA cm^{-2}	*Nat. Commun.* **10**, 5692 (2019)
IrCoNi PHNC	0.1 M $HClO_4$; carbon fiber paper	ca. 1.66	ca. 3 h @ 5 mA cm^{-2}	*Adv. Mater.* **29**, 1703798 (2017)
p-FGDY	0.5 M H_2SO_4; carbon cloth	1.80	5 h @ 10 mA cm^{-2}	*Angew. Chem. Int. Ed.* **58**, 13897-13903 (2019)

Supplementary Note 1. Determination of hydrogen binding energy (HBE) from underpotentially deposited hydrogen (H_{upd}) using cyclic voltammogram (CV) method

Hydrogen binding energy (HBE) on metal surface ΔH is defined by the enthalpy change of dissociative adsorption of H_2[9]

$$\frac{1}{2}H_2 + * \rightarrow H^* \tag{1}$$

Since this is a simplified model to compare HBEs qualitatively, we assume H adsorption obeys Langmuir isotherm with only considering the configurational entropy.[11] Thus, for Rxn (1) at temperature T,

$$\Delta G_1 = \Delta H - T\Delta S_{conf} = \Delta H - k_b T \ln\frac{1-\theta}{\theta} \tag{2}$$

where k_b is the Boltzmann constant and θ is the coverage of H.

As the adsorption/desorption process of H can be evaluated via

$$H^+ + e^- + * \leftrightarrows H^* \tag{3}$$

Rxn (3) – Rxn (1) can give the overall reaction of HER

$$H^+ + e^- \leftrightarrows \frac{1}{2}H_2 \tag{4}$$

The Gibbs free energy (ΔG_4^0) of Rxn (4) is zero at a potential of 0 V under standard conditions (T = 298 K, p_{H_2} = 1 atm and pH = 0).

Then the correlation between HBEs and the H_{upd} peak potentials can be derived in CVs assuming adsorption/desorption equilibrium throughout the measurements.[8]

Following the computational hydrogen electrode (CHE) model by Nørskov et al., one can assume that $\Delta G(H^+ + e^-)$ is equal to $-e_0E$ at any potential E (vs. standard hydrogen electrode (SHE)), which circumvents the explicit treatment of solvated proton in electrolyte solution.[12]

For Rxn (3), at potential E_3 (vs. SHE), $\Delta G_3 = -FE_3$, where F is the Faraday constant.

Since $E_{RHE} = E_{SHE} - \frac{RT\ln[H^+]}{F} = E_{SHE} + \frac{2.303RT(\text{pH})}{F}$,

$$E = E_3 + \frac{2.303RT(\text{pH})}{F} = -\frac{\Delta G_3}{F} + \frac{2.303RT(\text{pH})}{F} \tag{5}$$

where E is on the reversible hydrogen electrode (RHE) scale.

Under reaction conditions (T = 298 K, p_{H_2} = 1 atm and different a_{H^+}),

$$\Delta G_4 = \Delta G_4^{o} + RT\ln\frac{p_{H_2}^{1/2}}{a_{H^+}} = 2.303RT(\text{pH})$$

Because $\Delta G_4 = \Delta G_3 - \Delta G_1$,

$$\Delta G_3 = \Delta G_1 + 2.303RT(\text{pH}) \tag{6}$$

Substitute Eqs (2) and (6) into Eq (5),

$$E = -\frac{\Delta H}{F} + \frac{k_b T}{F}\ln\frac{1-\theta}{\theta} \tag{7}$$

Differentiate Eq (7) with respect to θ,

$$\frac{dE}{d\theta} = -\frac{k_b T}{F}\frac{1}{\theta(1-\theta)} \tag{8}$$

Assuming transfer of one electron per adsorbed H, the relationship between the charge transferred per area Q and the coverage θ is

$$Q = Q_{tot}\theta \tag{9}$$

where Q_{tot} is e times of the densities of active sites in the surface layer.[11]

During CVs, the relationship between current density $i(t)$ and the coverage θ is as follows:

$$i(t) = \pm\frac{dQ}{dt} = \pm Q_{tot}\frac{d\theta}{dt} \tag{10}$$

Substituting Eq (8) into Eq (10),

$$i(t) = \pm Q_{tot}\frac{d\theta}{dt} = \pm Q_{tot}\frac{d\theta}{dE}\frac{dE}{dt} = \pm Q_{tot}\frac{dE}{dt}\frac{F}{k_b t}\theta(1-\theta) \tag{11}$$

where $\pm\frac{dE}{dt}$ is the potential sweep rate.

At peak potential, current density $i(t)$ reaches the maximum value, so d$i(t)$/dθ will be 0. In Eq (11), when d$i(t)$/dθ = 0, θ is 0.5.

In Eq (7), at θ = 0.5,

$$E_{peak} = -\frac{\Delta H}{F} + \frac{k_b T}{F}\ln\frac{1-\theta}{\theta} = -\frac{\Delta H}{F} \tag{12}$$

Therefore, a direct link between the HBEs on metal surfaces and experimental CVs has been built by Eq (12).

Supplementary Note 2. Chemical principle of probing adsorption energy of OH* with methanol oxidation reaction (MOR)

OH* adsorbed on metal catalysts is an essential species in electrooxidation of methanol as well as the first intermediate for OER catalytic cycle.[13,14] A method of using alcohol molecules to probe reactive OH* generated in OER has been proposed recently.[15] The reaction between methanol and surface adsorbed OH* takes place very fast at low potentials, while its selectivity declines after the raise of OER because of the faster deprotonation of OH* and favourable kinetics of OER than MOR at high potentials.[16]

The detailed explanation of this mechanism is discussed as follows:

$$CH_2H_\beta\text{-}OH_\alpha + OH^- \leftrightarrows CH_2H_\beta\text{-}O^- + H_\alpha OH \quad (13)$$

where OH^- is present in base and from water dissociation in neutral or acidic media (α and β indicate the protons bonded at O and C, respectively).

$$M^* + OH^- \leftrightarrows M\text{-}OH^* + e^- \quad (14)$$

This is the first step in OER cycle, M* refers to the active site.

$$CH_2H_\beta\text{-}O^- + M\text{-}OH^* \rightarrow CH_2{=}O + H_\beta OH + M^* + e^- \quad (15)$$

The electrophilic OH* is nucleophilically attacked by $CH_2H_\beta\text{-}O^-$, which loses one electron to form formaldehyde.

$$CH_2{=}O + M\text{-}OH^* + OH^- \rightarrow HCOO^- + H_2O + M^* \quad (16)$$

Then formaldehyde can be oxidized to formate with the assistance of OH*.

From the mechanism presented above, it can be seen that adsorption energy of OH* can be probed by MOR qualitatively. If adsorption energy of OH* on active sites is too low in Eq (14), the catalyst surface will be free of OH*, and thus there is no response to methanol oxidation. However, when the adsorption energy of OH* is large enough, OH* will be accumulated on the surface of catalyst, which will contribute to MOR. Generally, lower onset potential of MOR means higher surface coverage of OH* as well as higher adsorption energy of OH*.

Supplementary References

1. Mahmood, J. et al. An efficient and pH-universal ruthenium-based catalyst for the hydrogen evolution reaction. *Nat. Nanotechnol.* **12**, 441-446 (2017).
2. Mahmood, J. et al. Encapsulating iridium nanoparticles inside a 3D cage-like organic network as an efficient and durable catalyst for the hydrogen evolution reaction. *Adv. Mater.* **30**, 1805606 (2018).
3. Sheng, Z.-H. et al. Catalyst-free synthesis of nitrogen-doped graphene via thermal annealing graphite oxide with melamine and its excellent electrocatalysis. *ACS Nano* **5**, 4350-4358 (2011).
4. Zhang, B.-W. et al. Long-life room-temperature sodium-sulfur batteries by virtue of transition metal nanocluster-sulfur interactions. *Angew. Chem. Int. Ed.* **131**, 1498-1502 (2019).
5. Yang, H. B. et al. Atomically dispersed Ni(I) as the active site for electrochemical CO_2 reduction. *Nat. Energy* **3**, 140-147 (2018).
6. Clancy, J. P. et al. Spin-orbit coupling in iridium-based 5*d* compounds probed by x-ray absorption spectroscopy. *Phys. Rev. B* **86**, 195131 (2012).
7. Oh, H.-S. et al. Electrochemical catalyst-support effects and their stabilizing role for IrO_x nanoparticle catalysts during the oxygen evolution reaction. *J. Am. Chem. Soc.* **138**, 12552-12563 (2016).
8. Zheng, J., Zhuang, Z., Xu, B. & Yan, Y. Correlating hydrogen oxidation/evolution reaction activity with the minority weak hydrogen-binding sites on Ir/C catalysts. *ACS Catal.* **5**, 4449-4455 (2015).
9. Sheng, W. et al. Correlating hydrogen oxidation and evolution activity on platinum at different pH with measured hydrogen binding energy. *Nat. Commun.* **6**, 5848 (2015).
10. Jiang, B. et al. Mesoporous metallic iridium nanosheets. *J. Am. Chem. Soc.* **140**, 12434-12441 (2018).
11. Karlberg, G. S. et al. Cyclic voltammograms for H on Pt(111) and Pt(100) from first principles. *Phys. Rev. Lett.* **99**, 126101 (2007).
12. Nørskov, J. K. et al. Origin of the overpotential for oxygen reduction at a fuel-cell cathode. *J. Phys. Chem. B* **108**, 17886-17892 (2004).
13. Zope, B. N., Hibbitts, D. D., Neurock, M. & Davis, R. J. Reactivity of the gold/water interface during selective oxidation catalysis. *Science* **330**, 74-78 (2010).
14. Reier, T., Nong, H. N., Teschner, D., Schlögl, R. & Strasser, P. Electrocatalytic oxygen evolution reaction in acidic environments – reaction mechanisms and catalysts. *Adv. Energy Mater.* **7**, 1601275 (2017).
15. Tao, H. B. et al. A general method to probe oxygen evolution intermediates at operating conditions.

Joule **3**, 1498-1509 (2019).

16. Rodriguez, P., Kwon, Y. & Koper, M. T. M. The promoting effect of adsorbed carbon monoxide on the oxidation of alcohols on a gold catalyst. *Nat. Chem.* **4**, 177-182 (2012).
