# Supplementary material for: Coordination engineering of iridium nanocluster bifunctional electrocatalyst for highly efficient and pH-universal overall water splitting
Source: Nat Commun. 2020 Aug 25;11:4246. doi: 10.1038/s41467-020-18064-w (PMC7447631; doi:10.1038/s41467-020-18064-w)
Supplement: Supplementary file 2 — Peer Review File [file 41467_2020_18064_MOESM2_ESM.docx]

Peer Review Information

**Journal:** Nature Communications

**Manuscript title:** Coordination engineering of iridium nanocluster bifunctional electrocatalyst for highly efficient and pH-universal overall water splitting

**Corresponding author name(s):** Prof Bin Liu

**Editorial notes:**

**Transferred manuscripts**

This manuscript has been previously reviewed at another journal that is not operating a transparent peer review scheme. This document only contains reviewer comments and rebuttal letters for versions considered at *Nature Communications*.

**Reviewer comments & decisions:**

**Reviewer comments, first version:**

Reviewers' comments:

Reviewer #1 (Remarks to the Author):

This manuscript by Liu et al. reported nitrogen and sulfur coordinated iridium nanoclusters embedded on nitrogen and sulfur co-doped graphene as an efficient electrocatalyst for overall water splitting in a wide range of pH values. This catalyst achieved very good activity with a geometric current density of 10 mA cm-2 at low voltages of 1.53, 1.42 and 1.45 V for overall water splitting in neutral, acidic and alkaline electrolytes, respectively, which is impressive, but not good enough without showing the voltages at current densities at 500 and 1000 mA/cm2, which is very necessary in order for the papers to be accepted. The authors also conducted different experimental measurements to study the HER and OER intermediates, and revealed the mechanisms by operando EXAFS spectroscopy and DFT calculations. I would like to recommend its publication in Nat. Commun. after the authors address the following issues.

1. Provide the characterization at current density of 500 and 1000 mA/cm2.
2. Is this synthesis method suitable for other metal nanoclusters embedded on nitrogen and sulfur co-doped graphene? For example, if iridium(III) chloride was replaced by FeCl3, can we synthesize Fe-N-S on N-S co-doped graphene?
3. Ir is a noble metal. We always want to obtain better performance with less Ir. What’s the loading amount of Ir in the best sample in this work? Is it possible to control the Ir amount by changing the amount of iridium(III) chloride? If so, please optimize the loading amount of Ir.
4. For the XPS analysis on page 6, “Intriguingly, negative shift was observed for S 2p3/2 (163.9 eV) and S 2p1/2 (165.0 eV) in the corresponding high-resolution XPS S 2p spectrum (Supplementary Fig. 10d), implying S might be immobilized through bonding in -C-S- and Ir-S.” Negative shift means lower binding energy of S, but if S was immobilized through bonding in -C-S- and Ir-S, the binding energy of S should be higher. Please double check this sentence.
5. The stability tests were finished in several hours, which are too short. Please test the stability in a longer time (at least 60 h in lab).
6. For practical water electrolysis, iR compensation will not be conducted. Please present the data without iR compensation for overall water splitting.


Reviewer #2 (Remarks to the Author):

In this paper, the authors successfully dispersed Ir nanoclusters coordinated with both nitrogen and sulfur on nitrogen and sulfur co-doped graphene, and used this material as an efficient and robust electrocatalyst for both HER and OER at all pH conditions. They report excellent performance with geometric current density of 10 mA cm-2 at as low as 300, 190 and 220 mV overpotential for overall water splitting in neutral, acidic and alkaline electrolyte, respectively. They have also performed systematic experiments to characterize the structure and conducted DFT calculations combined with operando XAS to investigate the intrinsic mechanism. Overall, this study is interesting and present a nice electrocatalyst for overall water-splitting. However, the following issues should be carefully addressed before the paper can be publised:

1. The co-doped structure is rather complex. The authors have conducted XPS, XAS to characterize the local binding environment of Ir, and found some robust evidence for the Ir-S and Ir-N binding. The TEM and EDX mapping also indicate that N and S are distributed around Ir nanoclusters. However, some detailed information is still not very clear. For example, is the same Ir coordinated with both S and N simultaneously? In this case, how the S and N dopants simultaneously (or synergistically) influence the activity of Ir toward HER and OER. Given the complex co-doped structure, I do not think the DFT calculations, particularly based on the simplified model, is convincing. For example, the calculated deltaG for HER are very close, i.e., 0.04 and -0.07 eV. Even a very small distortion or different binding can completely change the overall results. Have the authors considered the Ir nanoclusters on N-doped graphene or S-doped graphene as control experiments? The individual role of N and S in the electrocatalytic reactions should be elucidated thoroughly.
2. What is the influence of Ir content on the resulting structures and HER/OER performance?
3. I suggest the authors to include more comparison with more recently published literatures. The stability (in acidic media) should also be considered and compared.


Reviewer #3 (Remarks to the Author):

Wang and collaborator synthesized iridium nanoclusters coordinated with both nitrogen and sulfur and supported on nitrogen and sulfur co-doped graphene, and showed that the system can serve as a bifunctional HER and OER pH-universal catalyst. This contributes an important finding. The study employs a variety of experimental measurements and theoretical modeling based on DFT to understand the mechanism. Thus, the approach is of interest to the wider community. The paper is well written. While I find the study interesting especially the collaborative investigations at the atomic level, I had several concerns to have a convincing story. The SI can benefit from more details about the experiment and theory, and not just figures with captions.
1. It is not clear what is the role of N/S in the system based on experimental measurements? Do these improve the catalytic activity?
2. What is the experimental motivation for introducing the DFT models and the Ir-N-S coordination? Can the authors use the DFT models to validate the experimental measurements? Showing that these particular models yield good HER/OER is not sufficient. Experimentally supplementary Figure 7 shows an oxidized state of Ir. Why oxygen adatoms are not included in the models for HER? Given the low overpotential in the system, oxygen adsorbates could be stabilized.
3. Did the authors assess the long-term stability of the catalyst and whether this is accompanied by any variations in the structure of the catalyst?
4. In Figure 3a, the HBE for Ir in alkaline solution is listed as Ir (111) as well as Pt (110) and Pt (100). In operando X-ray absorption spectroscopy, I think only the Ir-NSG and Pt/C can be measured as a whole instead of each termination. This needs more elaboration.
5. I have strong concerns about the DFT modeling and the approach that I feel is too simplistic. Bulk Ir without N/S has already excellent activity without the need for N/S. Also, from Figure S21, I see that there are different adsorption sites with different adsorption strength. It is clearly shown that the first adsorption site (with dG = -0.04 eV) is the best because dG is very close to zero. However, there are several strong adsorption sites with dG from -0.4 to -0.6 eV, and the adsorption sites with dG around - 0.3 eV cannot be even considered as the high activity sites for the large current as shown in cyclic voltammogram. I believe the adsorbed hydrogen will fast diffuse to these strong adsorption sites before waiting for another hydrogen atom to be adsorbed to the nearby adsorption sites to form H2 through the Tafel reaction. The way to test the dG for these adsorption sites is to understand the most stable adsorption configurations at experimental conditions (different pH and at 300 K). This is because the H-H interactions are also a factor for dG. (Chem. Mater. 2020, 32, 1315−1321) To use a single dG to present for the experiment condition correctly, there are important assumptions that are often ignored in several DFT paper; the adsorption sites are needed to be identical, and the H-H interactions are almost independent to hydrogen coverage. (J. Phys. Chem. C 2010, 114, 42, 18182-18197). However, the Ir nanoparticle system does not meet the two requirements.
6. In Figure S17, the cyclic voltammogram of Ir shows only a peak, which seems that these is only one kind of adsorption site. However, the DFT calculation shows that there are several adsorption sites with different adsorption strengths indicated by different dG. Therefore, I expect that the CV may show more than one peak along the voltages.
7. It is not accurate to quantitatively compare dG with HBE obtained from operando X-ray absorption spectroscopy, for example, in line 275. The operando X-ray absorption measures the hydrogen binding energy at potential larger than 0 vs SHE, and this is the measure of the adsorption/desorption of H from/to HClO. However, dG is defined as the free energy between H2 and adsorbed H, which is free energy of HER. Also, dG includes the TdS of H2 gas (Journal of The Electrochemical Society, 152 (3) J23-J26 (2005))
8. In line 278, it is said that “Ir atom is positively charged”. However, in Figure 22, I see lot of cyan color in Ir, which indicates Ir atoms are negatively charged. (In the description, “The purple and cyan regions refer to positive and negative charge density distributions, respectively.”)
9. I am supersized that the Tafel slope for Ir and Pt in acidic environment is below 30 mV/dec. It has been discussed before that the mass transfer limitation for high HER active catalysts cannot be avoid using the rotating disk setting. Additionally, and importantly, the impendence from the electrolyte can also affect current measurements on fast HER electrodes even with highly conductive electrolytes such as 0.1 M HClO4. (Journal of The Electrochemical Society, 162 (14) F1470-F1481 (2015); Journal of The Electrochemical Society, 157 (11) B1529-B1536 (2010); Journal of The Electrochemical Society, 162 (1) F190-F203 (2015); Energy Environ. Sci., 2014, 7, 2255) Due to these limitations, 30 mV/dec should be always shown. More explanation should be provided.

**Author rebuttal, first version:**

**Responses to the remarks of Reviewer #1:**

*This manuscript by Liu et al. reported nitrogen and sulfur coordinated iridium nanoclusters embedded on nitrogen and sulfur co-doped graphene as an efficient electrocatalyst for overall water splitting in a wide range of pH values. This catalyst achieved very good activity with a geometric current density of 10 mA cm^-2^ at low voltages of 1.53, 1.42 and 1.45 V for overall water splitting in neutral, acidic and alkaline electrolytes, respectively, which is impressive, but not good enough without showing the voltages at current densities at 500 and 1000 mA/cm^2^, which is very necessary in order for the papers to be accepted. The authors also conducted different experimental measurements to study the HER and OER intermediates and revealed the mechanisms by operando EXAFS spectroscopy and DFT calculations. I would like to recommend its publication in Nat. Commun. after the authors address the following issues.*

**Response:** We appreciate the reviewer for the positive recommendations and constructive suggestions. To evaluate the performance of our Ir-NSG catalyst at large current densities, we have measured LSV curves of Ir-NSG coupled water electrolysis cell and showed the corresponding voltages at 500 and 1000 mA cm^-2^ in Fig. R1. The detailed discussion is presented in the following.

**Comment 1:** *Provide the characterization at current density of 500 and 1000 mA/cm^2^.*

**Response 1:** We thank the reviewer for the constructive suggestion. According to the reviewer’s suggestion, we have complemented the measurement of overall water splitting activities of Ir-NSG at large current densities and displayed the corresponding LSV curves in Fig. R1. As shown in Fig. R1, the cell with Ir-NSG anode and cathode requires only 2.58, 1.85 and 2.23 V to reach 500 mA cm^-2^ and 2.67, 1.95 and 2.50 V to reach 1000 mA cm^-2^ in 1 M PBS, 0.1 M HClO_4_ and 1 M KOH, respectively, outperforming the coupled benchmark Ir/C || Pt/C electrolyzer.

**Fig. R1 and revised Supplementary Figure 31.** **Overall-water-splitting performance.** LSV curves of Ir-NSG and Ir/C-Pt/C coupled water electrolysis cell at a scan rate of 10 mV s^-1^ in **a,** 1 M PBS, **b,** 0.1 M HClO_4_, and **c,** 1 M KOH. Catalyst loading: 73.3 μg_metal_ cm^-2^. All measurements were conducted in Ar-saturated electrolytes and corrected for 85% *i*-R drop.

Correspondingly, we have added Fig. R1 as Supplementary Fig. 31, and added necessary statements in lines 339-341 of the revised manuscript as “Specially, we further explored the water electrolysis performance at large current densities (500 and 1000 mA cm^-2^) as shown in Supplementary Fig. 31, highlighting the potential of Ir-NSG for practical overall water splitting.”.

**Comment 2:** *Is this synthesis method suitable for other metal nanoclusters embedded on nitrogen and sulfur co-doped graphene? For example, if iridium(III) chloride was replaced by FeCl_3_, can we synthesize Fe-N-S on N-S co-doped graphene?*

**Response 2:** This is an excellent question! We thank the reviewer for raising the helpful comment and interest in our synthesis method. Indeed, we can synthesize a series of metal nanoclusters embedded on nitrogen and sulfur co-doped graphene using the corresponding raw materials via our method. To prove the universality of this synthesis method, we have successfully synthesized Fe nanoclusters loaded on N and S co-doped graphene (Fe-NSG) using FeCl_3_ in substitution for IrCl_3_·xH_2_O. Firstly, a mixture comprising of melamine (99 %, Sigma Aldrich), L-cysteine (≥ 98 %, Sigma Aldrich) and iron(III) chloride (97 %, Sigma Aldrich) was ball-milled until reaching a homogeneous precursor. Subsequently, the fine powder mixture was undergone a two-stage pyrolysis in a tubular furnace under argon atmosphere. The first stage was from 25 to 600 °C at a ramping rate of 2.5 °C min^-1^ and maintained at 600 °C for 2 h, while the second stage was from 600 to 800 °C at a ramping rate of 2.5 °C min^-1^ and maintained at 800 °C for 1 h. The bright-field (BF) TEM images of Fe-NSG are provided in Fig. R2.

**Fig. R2. Bright-field (BF) TEM images of the as-prepared Fe-NSG. a,** BF TEM image showing the homogeneous distribution of Fe nanoclusters within the graphene matrix, scale bar: 20 nm. **b,** BF HR-STEM image of Fe-NSG, scale bar: 5 nm.

**Comment 3:** *Ir is a noble metal. We always want to obtain better performance with less Ir. What’s the loading amount of Ir in the best sample in this work? Is it possible to control the Ir amount by changing the amount of iridium(III) chloride? If so, please optimize the loading amount of Ir.*

**Response 3:** We thank the reviewer for the valuable comments and suggestions. It is possible to control the Ir amount in the final product by changing the amount of IrCl_3_·xH_2_O. In fact, the Ir-NSG that we presented in the original manuscript, whose loading amount of Ir is 7.33 wt. %, is the optimized sample in this work. To address the reviewer’s concerns, we have added the details of optimization process in the revised Supplementary Information. Firstly, we synthesized four Ir-NSG samples with Ir mass content of 4.28, 5.97 7.33 and 8.59 wt. % by changing the amount of IrCl_3_·xH_2_O in the synthesis process, respectively. The morphology of the three control samples are shown in Fig. R3. Then, the effect of Ir amount on the HER and OER performance and the corresponding mass activities in 1 M PBS were evaluated and are presented in Fig. R4. As a conclusion, the sample of Ir-NSG-7.33 wt. % shows the lowest overpotential at 10 mA cm^-2^ and largest mass activities for both HER and OER.

**Fig. R3 and revised Supplementary Figure 11. Bright-field TEM images of samples with various Ir loading amounts. a,** Ir-NSG-4.28 wt. %. **b,** Ir-NSG-5.97 wt. %. **c,** Ir-NSG-8.59 wt. %. Scale bars: 20 nm.

**Fig. R4 and revised Supplementary Figure 12.** **HER and OER performance of Ir-NSG electrocatalysts with different Ir mass contents in neutral electrolyte. a,** HER polarization curves acquired at a sweep rate of 5 mV s^-1^ in Ar-saturated 1 M PBS. The dash-dot line serves as the eye guide for the comparison of the overpotentials at 10 mA cm^-2^. **b,** Comparison of Ir-mass specific activities at -0.015 V (vs. RHE). **c,** OER polarization curves acquired at a sweep rate of 5 mV s^-1^ in 1 M PBS. The dash-dot line serves as the eye guide for the comparison of the overpotentials at 10 mA cm^-2^. **d,** Comparison of Ir-mass specific activities at 1.53 V (vs. RHE).

Figs. R3 and R4 have been added as Supplementary Figs. 11 and 12 in the revised Supplementary Information, respectively. Accordingly, we have changed the statements in lines 113-116 to “The mass content of Ir in Ir-NSG determined by XPS is ~8.34 wt. % and close to that measured by inductively coupled plasma atomic emission spectroscopy (ICP-AES) (7.33 wt. %), which is the optimized sample on the basis of Ir loading amount (Supplementary Figs. 11 and 12)” in the revised manuscript.

**Comment 4:** *For the XPS analysis on page 6, “Intriguingly, negative shift was observed for S 2p_3/2_ (163.9 eV) and S 2p_1/2_ (165.0 eV) in the corresponding high-resolution XPS S 2p spectrum (Supplementary Fig. 10d), implying S might be immobilized through bonding in -C-S- and Ir-S.” Negative shift means lower binding energy of S, but if S was immobilized through bonding in -C-S- and Ir-S, the binding energy of S should be higher. Please double check this sentence.*

**Response 4:** We thank the reviewer for the kind suggestion. Firstly, this phenomenon of negative shift of S 2*p* spectra can indeed be observed in Supplementary Fig. 10d. Moreover, as the electronegativity of S element is larger than C and Ir, the lower binding energy of S can explain the immobilization of S through accepting electrons from C and Ir to form -C-S- and Ir-S bonds (Zhang, B.-W. et al. *Angew. Chem. Int. Ed.* 2019, **131,** 1498-1502).

Correspondingly, the statements in lines 119-122 of the manuscript have been revised as “Intriguingly, negative shift is observed for S 2*p*_3/2_ (163.9 eV) and S 2*p*_1/2_ (165.0 eV) in the corresponding high-resolution XPS S 2*p* spectrum (Supplementary Fig. 10d), implying that S might be immobilized through bonding in -C-S- and Ir-S in consideration of its larger electronegativity than those of C and Ir.”.

**Comment 5:** *The stability tests were finished in several hours, which are too short. Please test the stability in a longer time (at least 60 h in lab).*

**Response 5:** We appreciate the reviewer’s constructive comment and suggestion. The stability is another important figure-of-merit for electrocatalysts. Following the reviewer’s suggestion, we tested the long-term stabilities of Ir-NSG for overall water splitting at 10 mA cm^-2^ in a two-electrode water electrolysis cell (Fig. 4a) in 1 M PBS, 0.1 M HClO_4_ and 1 M KOH solution, respectively. As shown in Fig. R5, there are slight degradations over a 24-hour operation, which are among the most stable overall-water-splitting electrocatalysts reported to date, especially in acidic media (Table R1).

**Fig. R5.** Chronopotentiometric curves of Ir-NSG coupled water electrolysis cell at 10 mA cm^-2^ in **a,** 1 M PBS, **b,** 0.1 M HClO_4_, and **c,** 1 M KOH. All measurements were conducted in Ar-saturated electrolytes without *i*-R drop correction.

**Table R1 and revised Supplementary Table 6.** Comparison of overall-water-splitting activities and stabilities with various recently reported state-of-the-art electrocatalysts in acidic electrolyte.

| *Catalyst* | *Medium and electrode* | *Cell Voltage (V)* | *Durability @ 10 mA cm^-2^ (h)* | *Reference* |
| --- | --- | --- | --- | --- |
| Ir-NSG | 0.1 M HClO_4_; carbon fiber paper | 1.42 | 24 h @ 10 mA cm^-2^ | This work |
| Li-IrSe_2_ | 0.5 M H_2_SO_4_; carbon fiber paper | 1.44 | 24 h @ 1.47 V | *Angew. Chem. Int. Ed.* **58**, 14764-14769 (2019) |
| CoMoNiS-NF-31 | 0.5 M H_2_SO_4_; carbon fiber paper | 1.45 | 80 min @ 1.53 V | *J. Am. Chem. Soc.* **141**, 10417-10430 (2019) |
| IrW/C | 0.5 M H_2_SO_4_; carbon fiber paper | 1.45 | 8 h @ 10 mA cm^-2^ | *ACS Cent. Sci.* **4**, 1244-1252 (2018) |
| RuIrO_x_ | 0.5 M H_2_SO_4_; carbon fiber paper | 1.45 | 24 h @ 10 mA cm^-2^ | *Nat. Commun.* **10**, 4875 (2019) |
| RuCu NCs | 0.5 M H_2_SO_4_; N/A | 1.49 | 15 h @ 10 mA cm^-2^ | *Angew. Chem. Int. Ed.* **58**, 13983-13988 (2019) |
| Co-RuIr | 0.1 M HClO_4_; N/A | 1.52 | 25 h @ 10 mA cm^-2^ | *Adv. Mater.* **31**, 1900510 (2019) |
| a-RuTe_2_ PNRs | 0.5 M H_2_SO_4_; N/A | 1.52 | 24 h @ 7 mA cm^-2^ | *Nat. Commun.* **10**, 5692 (2019) |
| IrCoNi PHNC | 0.1 M HClO_4_; carbon fiber paper | ca. 1.66 | ca. 3 h @ 5 mA cm^-2^ | *Adv. Mater.* **29**, 1703798 (2017) |
| p-FGDY | 0.5 M H_2_SO_4_; carbon cloth | 1.80 | 5 h @ 10 mA cm^-2^ | *Angew. Chem. Int. Ed.* **58**, 13897-13903 (2019) |

Accordingly, we have added the Fig. R5 and Table R1 as insets in Figs. 4b, c, d and Supplementary Table 6 in the revised manuscript and Supplementary Information, respectively. And the corresponding statements in lines 341-345 of the manuscript have been revised as “Insets in Figs. 4b, c, and d manifest that the electrolyzer assembled with Ir-NSG anode and cathode can retain a current density of 10 mA cm^-2^ over long-term continuous tests with slight voltage decay and no detectable structure degradation at all pH values (Supplementary Fig. 32), which is among the most robust overall-water-splitting catalysts reported to date, especially in acidic media (Supplementary Table 6).”.

**Comment 6:** *For practical water electrolysis, iR compensation will not be conducted. Please present the data without iR compensation for overall water splitting.*

**Response 6:** We thank the reviewer for the kind suggestion. We have provided the overall-water-splitting polarization curves without *i*R compensation in Fig. R6. Unfortunately, as we mainly focused on the investigation of intrinsic activities and mechanism of Ir-NSG in this work, we measured the overall-water-splitting performance using only catalysts decorated on carbon papers in a homemade two-electrode cell (Fig. 4a). The cell resistance is 26.5, 29.7 and 14.9 ohm in 1 M PBS, 0.1 M HClO_4_ and 1 M KOH, respectively, which are quite large. In our following work, we will devote more efforts on fabrication of membrane-electrode-assembly and cell setup to minimize uncompensated series resistance in order to investigate the practical application of Ir-NSG catalyst.

**Fig. R6.** LSV curves of Ir-NSG and Ir/C-Pt/C coupled water electrolysis cell at a scan rate of 10 mV s^-1^ in **a,** 1 M PBS, **b,** 0.1 M HClO_4_, and **c,** 1 M KOH. Catalyst loading: 73.3 μg_metal_ cm^-2^. All measurements were conducted in Ar-saturated electrolytes and without *i*-R drop correction.

**Responses to the remarks of Reviewer #2:**

*In this paper, the authors successfully dispersed Ir nanoclusters coordinated with both nitrogen and sulfur on nitrogen and sulfur co-doped graphene, and used this material as an efficient and robust electrocatalyst for both HER and OER at all pH conditions. They report excellent performance with geometric current density of 10 mA cm^-2^ at as low as 300, 190 and 220 mV overpotential for overall water splitting in neutral, acidic and alkaline electrolyte, respectively. They have also performed systematic experiments to characterize the structure and conducted DFT calculations combined with operando XAS to investigate the intrinsic mechanism. Overall, this study is interesting and present a nice electrocatalyst for overall water-splitting. However, the following issues should be carefully addressed before the paper can be published:*

**Response:** We thank the reviewer for the positive comments.

**Comment 1:** *The co-doped structure is rather complex. The authors have conducted XPS, XAS to characterize the local binding environment of Ir, and found some robust evidence for the Ir-S and Ir-N binding. The TEM and EDX mapping also indicate that N and S are distributed around Ir nanoclusters. However, some detailed information is still not very clear. For example, is the same Ir coordinated with both S and N simultaneously? In this case, how the S and N dopants simultaneously (or synergistically) influence the activity of Ir toward HER and OER. Given the complex co-doped structure, I do not think the DFT calculations, particularly based on the simplified model, is convincing. For example, the calculated deltaG for HER are very close, i.e., 0.04 and -0.07 eV. Even a very small distortion or different binding can completely change the overall results. Have the authors considered the Ir nanoclusters on N-doped graphene or S-doped graphene as control experiments? The individual role of N and S in the electrocatalytic reactions should be elucidated thoroughly.*

**Response 1:** We thank the reviewer for raising this meaningful question. We realize that the evidence and discussion in the original manuscript are not enough to elucidate how the N and S dopants synergistically influence the activities of Ir. To fully clarify the role of N and S, we took the reviewer’s suggestions and synthesized N, S-doped graphene (NSG) and Ir nanoclusters on N-doped graphene (Ir-NG) as control samples and conducted DFT calculations on Ir-NG model in this revision. On the basis of these additional experimental and computational results, we conclude that the Ir sites coordinated with both N and S are responsible for the enhanced activities of Ir-NSG. The detailed interpretations are presented from both the experimental and theoretical aspects as listed below:

**i) From experimental aspect:**

We synthesized pristine NSG without adding iridium(III) chloride hydrate and the control sample of Ir-NG using L-alanine (C_3_H_7_NO_2_) in place of L-cysteine (C_3_H_7_NO_2_S). The mesoporous and sheet-like structure of NSG is consistent with that of Ir-NSG (Fig. R7). Moreover, as revealed by the BF TEM image and STEM-EDX elemental mappings of Ir-NG, the Ir nanoclusters are homogeneously distributed in the N-doped graphene framework (Fig. R8). The mass content of Ir in Ir-NG determined by ICP-AES is 7.07 wt. %, which is close to that of Ir-NSG. The chemical composition and valence states at the surface of Ir-NG were further examined by XPS (Fig. R9). The high-resolution XPS N 1*s* spectrum could be deconvoluted into Ir-N, pyridinic, pyrrolic, graphitic and oxidized N species, respectively (Fig. R9b), and the N of Ir-N is in the pyridinic form (Yang, H. B. et al. *Nat. Energy* 2018, **3,** 140-147). Intriguingly, the corresponding binding energy of Ir for Ir-NG is lower than that for IrO_2_ but higher than that for Ir-NSG as highlighted by the dash line, in line with the medium electronegativity of N comparing with that of O and S (Fig. R9c).

To verify the individual role of N coordination in the electrocatalytic reactions, we added the HER and OER measurements of NSG and Ir-NG. Fig. R10 shows the HER and OER polarization curves of Ir-NSG in comparison with NSG, Ir-NG and benchmark Pt/C and Ir/C in neutral, acidic and alkaline electrolytes. It can be found that the HER and OER activities of Ir-NSG are dramatically higher than those of pristine NSG, indicating that Ir can effectively boost HER and OER while N and S are not the active sites. Furthermore, it should also be noted that although Ir-NG can enhance the HER and OER activities, they are still far inferior to those of Ir-NSG.

**Fig. R7 and revised Supplementary Figure 15.** Typical SEM images of NSG, showing the same morphology with Ir-NSG. Scale bars are 3 μm and 300 nm for **a** and **b**, respectively.

**Fig. R8 and revised Supplementary Figure 16. Morphology of Ir-NG. a,** Bright-field TEM image of Ir-NG (with 7.07 wt. %), showing that Ir nanoclusters are homogeneously distributed in the N-doped graphene framework. Scale bar: 20 nm. **b,** Dark-field STEM image and EDX elemental mappings, scale bars: 5 nm.

**Fig. R9 and revised Supplementary Figure 17. XPS spectra of Ir-NG. a,** Survey spectrum of Ir-NG, revealing the absence of S (shadow area) and successful doping of N into graphene. **b,** High-resolution XPS N 1*s* spectrum, confirming the presence of Ir-N coordination in the pyridinic form. **c,** High-resolution XPS Ir 4*f* spectra. The corresponding binding energy of Ir for Ir-NG is between that for Ir-NSG and IrO_2_ as highlighted by the dash line, in accordance with the medium electronegativity of N comparing to that of O and S.

**Fig. R10. HER and OER performance of the Ir-NSG catalyst in comparison with NSG and Ir-NG. a,** HER polarization curves acquired in Ar-saturated 1 M PBS. **b,** OER polarization curves acquired in 1 M PBS. **c,** HER polarization curves acquired in Ar-saturated 0.1 M HClO_4_. **d,** OER polarization curves acquired in 0.1 M HClO_4_. **e,** HER polarization curves acquired in Ar-saturated 1 M KOH. **f,** OER polarization curves acquired in 1 M KOH. All measurements were calibrated with *i*R-compensation. Catalyst loading: 0.3 mg cm^-2^. Rotation speed: 1,600 rpm. The LSV curves were obtained at a scan rate of 1 mV s^-1^.

Correspondingly, we have added the Figs. R7, R8 and R9 as Supplementary Figs. 15, 16 and 17 in the revised Supplementary Information, respectively. Moreover, Figs R10a, b, c, d, e, f have been added as Figs. 2a, 2e and Supplementary Figs. 18a, 20a, 19a and 21a in the revised manuscript and Supplementary Information. We have also added the statement of “From the polarization curves of HER and OER, it can be found that Ir-NSG can effectively boost both HER and OER in comparison with pristine NSG. Moreover, although Ir-NG can enhance the activities of HER and OER, they are still far inferior to those of Ir-NSG. All these results imply that Ir coordinated with both N and S could be the real active site in Ir-NSG.” in the lines of 262-266 in the revised manuscript.

**ii) From theoretical aspect:**

To compare the HER and OER activities of Ir nanoclusters on NSG and NG, the free-energy diagrams of Ir-NG were also calculated, as shown in Figs. R11 and R12. The structure of Ir-NG was constructed based on the model structure of Ir_13_@NSG (Ir-NSG, see revised Supplementary Fig. 25). It can be seen from Fig. R11 that the HER activity of Ir-NG is much lower than that of Ir-NSG, with a larger energy barrier of 0.37 eV, in good agreement with the above-mentioned experimental results. Besides, although the potential determining step (PDS) of Ir-NG for OER is the same as that of Ir-NSG (formation of OOH* from O*), an increased apparent energy barrier from 0.53 eV of Ir-NSG to 0.75 eV of Ir-NG is observed for the PDS (Fig. R12), indicating that N, S-coordinated Ir is the efficient active site.

**Fig. R11.** DFT predicted free-energy diagrams for HER at U = 0 V on Ir-NG (cyan, magenta and orange lines), in comparison with Ir-NSG (red line). Hydrogen adsorption configurations on different Ir sites were considered, where the most favorable Ir site is shown in cyan circle.

**Fig. R12.** DFT predicted free-energy diagrams for OER at U = 1.23 V on Ir-NG (cyan line), compared with Ir-NSG (red line).

In addition, we agree with the reviewer that the Ir-NSG catalyst in experiments is very complicated. Here, in the Ir_13_@NSG model, we have considered the most significant factors that affect the activities based on the experimental observations. First of all, the average diameter of Ir nanocluster is about 1.55 nm, which consists of more than 100 Ir atoms. Thus, Ir nanocluster of 1.55 nm embedded in graphene will require a very large slab structure, which is extremely time-consuming in DFT calculations. Moreover, the effect of different coordination environments of Ir site is the main focus in this work. Therefore, Ir_13_ was used as the simplified model cluster, which includes both core-shell structure and various coordination states. Carbon vacancies of appropriate size are built in graphene to embed the Ir_13_ cluster. Besides, as Ir-N and Ir-S coordinations are identified in experiments, the twelve atoms coordinated to Ir_13_ are replaced by ten N atoms and two S atoms. Meanwhile, as can be seen from structures of intermediates during HER and OER, minimized structural rearrangement is maintained to avoid energy difference caused by distortion. In this way, the energies of different intermediate-adsorption configurations and reaction pathways are comparable. Furthermore, comparisons of HER and OER activities between Ir-NSG and Ir-NG provided a deeper understanding of the real active site. The calculated ∆*G*_H*_ and (∆*G*_OOH*_ − ∆*G*_O*_) at Ir_S_* site of Ir-NSG are favored for both HER and OER while Ir_N_* sites of Ir-NG show evidently lower activities, further illustrating the high performances of the obtained N, S co-coordinated structure.

**Comment 2**: *What is the influence of Ir content on the resulting structures and HER/OER performance?*

**Response 2:** We thank the reviewer for the valuable question. It is possible to control the Ir amount in the final product by changing the amount of IrCl_3_·xH_2_O. In fact, the Ir-NSG that we presented in the original manuscript, whose loading amount of Ir is 7.33 wt. %, is the optimized sample in this work. To address the reviewer’s concerns, we have added the details of optimization process in the revised Supplementary Information. Firstly, we synthesized four Ir-NSG samples with Ir mass content of 4.28, 5.97 7.33 and 8.59 wt. % by changing the amount of IrCl_3_·xH_2_O in the synthesis process, respectively. The structures of the three control samples are shown in Fig. R3. The densities of Ir nanoclusters increase with Ir content, and when the Ir content increases to 8.59 wt.%, agglomeration of Ir nanoclusters can be observed. Then, the influence of Ir amount on the HER and OER performance and the corresponding mass activities in 1 M PBS were evaluated and presented in Fig. R4. As a conclusion, the sample of Ir-NSG-7.33 wt. % shows the lowest overpotential at 10 mA cm^-2^ and largest mass activities for both HER and OER.

**Fig. R3 and revised Supplementary Figure 11. Bright-field TEM images of samples with various Ir loading amounts. a,** Ir-NSG-4.28 wt. %. **b,** Ir-NSG-5.97 wt. %. **c,** Ir-NSG-8.59 wt. %. Scale bars: 20 nm.

**Fig. R4 and revised Supplementary Figure 12. HER and OER performance of Ir-NSG electrocatalysts with different Ir mass contents in neutral electrolyte. a,** HER polarization curves acquired at a sweep rate of 5 mV s^-1^ in Ar-saturated 1 M PBS. The dash-dot line serves as the eye guide for the comparison of the overpotentials at 10 mA cm^-2^. **b,** Comparison of Ir-mass specific activities at -0.015 V (vs. RHE). **c,** OER polarization curves acquired at a sweep rate of 5 mV s^-1^ in 1 M PBS. The dash-dot line serves as the eye guide for the comparison of the overpotentials at 10 mA cm^-2^. **d,** Comparison of Ir-mass specific activities at 1.53 V (vs. RHE).

Figs. R3 and R4 have been added as Supplementary Figs. 11 and 12 in the revised Supplementary Information, respectively. Accordingly, we have changed the statements in lines 113-116 to “The mass content of Ir in Ir-NSG determined by XPS is ~8.34 wt. % and close to that measured by inductively coupled plasma atomic emission spectroscopy (ICP-AES) (7.33 wt. %), which is the optimized sample on the basis of Ir loading amount (Supplementary Figs. 11 and 12)” in the revised manuscript.

**Comment 3:** *I suggest the authors to include more comparison with more recently published literatures. The stability (in acidic media) should also be considered and compared.*

**Response 3:** We appreciate the reviewer for the helpful suggestion. Following the reviewer’s suggestion, we have summarized more exciting works published recently for comparison in Tables R2, R3, R4, R5 and R6. We also listed these catalysts in Fig. R13 for a clearer comparison. The results show that our Ir-NSG catalyst remains at the top among the best HER and OER catalysts at all pH conditions. Moreover, as an important figure-of-merit for catalysts, we tested the long-term stability of Ir-NSG for overall water splitting at 10 mA cm^-2^ in a two-electrode water electrolysis cell in 0.1 M HClO_4_ (Fig. R5) and compared it with other materials in recently published literatures (Table R1), showing that our Ir-NSG catalyst is among the most stable overall-water-splitting catalysts reported to date in acidic media.

**Table R2 and revised Supplementary Table 1.** Comparison of HER activities with various recently reported state-of-the-art catalysts in neutral electrolyte.

| *Catalyst* | *Medium and electrode* | *Scan rate (mV s^-1^)* | *η @ 10 mA cm^-2^ (mV)* | *Tafel slope (mV dec^-1^)* | *Reference* |
| --- | --- | --- | --- | --- | --- |
| Ir-NSG | 1 M PBS; glassy carbon | 1 | 22 | 21.2 | This work |
|  |  | 5 | 16.8 |  |  |
| CrO_x_/Cu-Ni | 1 M PBS; 3D CuO nanowire scaffold | N/A | 48 | 64 | *Nat. Energy* **4**, 107-114 (2019) |
| CoP/Co-MOF | 1 M PBS; carbon fiber paper | 1 | 49 | 63 | *Angew. Chem. Int. Ed.* **58**, 4679-4684 (2019) |
| Li_x_NiO/Ni | 1 M PBS; glassy carbon | 5 | 50 | 66 | *J. Am. Chem. Soc.* DOI: 10.1021/jacs.0c00241 (2020) |
| RuP_2_@NPC | 1 M PBS; glassy carbon | 5 | 57 | 87 | *Angew. Chem. Int. Ed.* **129**, 11717-11722 (2017) |
| NiCo_2_P_x_ | 1 M PBS; carbon felt | 5 | 63 | 63.3 | *Adv. Mater.* **29**, 1605502 (2017) |
| N-Ni | 1 M PBS; Ni foam | N/A | 64 | 106 | *J. Am. Chem. Soc.* **139**, 12283-12290 (2017) |
| Ni/WC@NC | 1 M PBS; N/A | 5 | 73 | 80.9 | *Energy Environ. Sci.* **11**, 2114-2123 (2018) |
| B-CoP/CNT | 1 M PBS; glassy carbon | 2 | 79 | 80 | *Angew. Chem. Int. Ed.* **59**, 4154-4160 (2020) |
| Mn-NiO-Ni | 1 M PBS; Ni foam | 1 | 80 | 121 | *Energy Environ. Sci.* **11**, 1898-1910 (2018) |
| PdP_2_@CB | 1 M PBS; glassy carbon | 5 | 84.6 | 72.3 | *Angew. Chem. Int. Ed.* **57**, 14862-14867 (2018) |
| Ni-C-N NSs | 1 M PBS; glassy carbon | 2 | 92.1 | 38 | *J. Am. Chem. Soc.* **138**, 14546-14549 (2016) |
| Ru@CN-0.16 | 1 M PBS; glassy carbon | 5 | ca. 100 | N/A | *Energy Environ. Sci.* **11**, 800-806 (2018) |
| Karst NF | 1 M PBS; Ni foam | 5 | 110 | 99 | *Energy Environ. Sci.* **13**, 174-182 (2020) |
| CoMoNiS-NF-31 | 1 M PBS; Ni foam | 5 | 117 | 56 | *J. Am. Chem. Soc.* **141**, 10417-10430 (2019) |
| NFP/C-3 | 1 M PBS; carbon fiber paper | 5 | 117 | 70 | *Sci. Adv.* **5**, eaav6009 (2019) |
| Li-IrSe_2_ | 1 M PBS; glassy carbon | 2 | 120 | N/A | *Angew. Chem. Int. Ed.* **58**, 14764-14769 (2019) |
| Ni_0.1_Co_0.9_P | 1 M PBS; carbon fiber paper | 5 | 125 | 103 | *Angew. Chem. Int. Ed.* **130**, 15671-15675 (2018) |
| MHCF-CNTs@PEMAc | 1 M PBS; glassy carbon | 5 | 278 | 108 | *Angew. Chem. Int. Ed.* **58**, 16217-16222 (2019) |
| p-FGDY | 1 M PBS; carbon cloth | 1 | 623 | 300 | *Angew. Chem. Int. Ed.* **58**, 13897-13903 (2019) |

**Table R3 and revised Supplementary Table 2.** Comparison of HER activities with various recently reported state-of-the-art noble metal-based catalysts in acidic electrolyte.

| *Catalyst* | *Medium and electrode* | *Scan rate (mV s^-1^)* | *η @ 10 mA cm^-2^ (mV)* | *Tafel slope (mV dec^-1^)* | *Reference* |
| --- | --- | --- | --- | --- | --- |
| Ir-NSG | 0.1 M HClO_4_; glassy carbon | 1 | 17 | 19.2 | This work |
|  |  | 5 | 5.3 |  |  |
| PtNC/S-C | 0.5 M H_2_SO_4_; glassy carbon | 2 | 11 | 24 | *Nat. Commun.* **10**, 4977 (2020) |
| Ru@MWCNT | 0.5 M H_2_SO_4_; glassy carbon | 5 | 13 | 27 | *Nat. Commun.* **11**, 1278 (2020) |
| AL-Pt/Pd_3_Pb | 0.5 M H_2_SO_4_; glassy carbon | N/A | 13.8 | 18 | *J. Am. Chem. Soc.* **141**, 19964-19968 (2019) |
| Pt-GT-1 | 0.5 M H_2_SO_4_; glassy carbon | 5 | 15 | N/A | *Nat. Energy* **3**, 773-782 (2018) |
| Pt_1_/hNCNC-2.92 | 0.5 M H_2_SO_4_; glassy carbon | 5 | 15 | 24 | *Nat. Commun.* **10**, 1657 (2019) |
| Pt_1_/N-C | 0.5 M H_2_SO_4_; glassy carbon | 5 | 19 | 14.2 | *Nat. Commun.* **11**, 1029 (2020) |
| RuCu NSs | 0.5 M H_2_SO_4_; glassy carbon | 5 | 19 | 26 | *Angew. Chem. Int. Ed.* **58**, 13983-13988 (2019) |
| Pt_5_/HMCS | 0.5 M H_2_SO_4_; glassy carbon | 1 | 20.7 | 28.3 | *Adv. Mater.* **32**, 1901349 (2020) |
| EG-Pt/CoP-1.5 | 0.5 M H_2_SO_4_; carbon fiber paper | 5 | 21 | 42.5 | *Energy Environ. Sci.* **12**, 2298-2304 (2019) |
| PtW_6_O_24_/C | 0.5 M H_2_SO_4_; glassy carbon | 5 | 22 | 29.8 | *Nat. Commun.* **11**, 490 (2020) |
| Ru@C_2_N | 0.5 M H_2_SO_4_; glassy carbon | 5 | 22 | 30 | *Nat. Nanotechnol.* **12**, 441-446 (2017) |
| Pt Sas/DG | 0.5 M H_2_SO_4_; carbon fiber paper | 5 | 23 | 25 | *J. Am. Chem. Soc.* **141**, 4505-4509 (2019) |
| A-CoPt-NC | 0.5 M H_2_SO_4_; glassy carbon | 5 | 27 | 31 | *Angew. Chem. Int. Ed.* **58**, 9404-9408 (2019) |
| Pt-AC/DG-3 | 0.1 M HclO_4_; glassy carbon | 5 | 29 | 34.8 | *J. Am. Chem. Soc.* **142**, 5594-5601 (2020) |
| Mo_2_TiC_2_T_x_-Pt_SA_ | 0.5 M H_2_SO_4_; carbon paper | 5 | 30 | 30 | *Nat. Catal.* **1**, 985-992 (2018) |
| L-Ag NPs | 0.5 M H_2_SO_4_; glassy carbon | 5 | 32 | 31 | *Nat. Catal.* **2**, 1107-1114 (2019) |
| a-RuTe_2_ PNRs | 0.5 M H_2_SO_4_; glassy carbon | N/A | 33 | 35 | *Nat. Commun.* **10**, 5692 (2019) |
| Pt_1_/OLC | 0.5 M H_2_SO_4_; glassy carbon | 5 | ca. 38 | 36 | *Nat. Energy* **4**, 512-518 (2019) |
| ALD50Pt/NG-Ns | 0.5 M H_2_SO_4_; glassy carbon | 2 | ca. 39 | 29 | *Nat. Commun.* **7**, 13638 (2016) |
| Pt SA/m-WO_3-x_ | 0.5 M H_2_SO_4_; glassy carbon | 5 | 47 | 45 | *Angew. Chem. Int. Ed.* **58**, 16038-16042 (2019) |
| Pt-Ru dimer | 0.5 M H_2_SO_4_; glassy carbon | 2 | 50 | 28.9 | *Nat. Commun.* **10**, 4936 (2019) |
| Pd NPs-Bis-24h | 0.5 M H_2_SO_4_; glassy carbon | 5 | 59.6 | 30 | *Adv. Mater.* **32**, 1902964 (2020) |
| Pt-GT-1 | 0.1 M HclO_4_; glassy carbon | 2 | 66 | 24 | *Nat. Energy* **3**, 773-782 (2018) |
| IrNiTa/Si | 0.5 M H_2_SO_4_; Ti | 2 | 99 | 35 | *Adv. Mater.* **32**, 1906384 (2020) |

**Table R4 and revised Supplementary Table 3.** Comparison of HER activities with various recently reported state-of-the-art noble metal-based catalysts in alkaline electrolyte.

| *Catalyst* | *Medium and electrode* | *Scan rate (mV s^-1^)* | *η @ 10 mA cm^-2^ (mV)* | *Tafel slope (mV dec^-1^)* | *Reference* |
| --- | --- | --- | --- | --- | --- |
| Ir-NSG | 1 M KOH; glassy carbon | 1 | 18.5 | 28.3 | This work |
|  |  | 5 | 14.3 |  |  |
| Ru@MWCNT | 1 M KOH; glassy carbon | 5 | 17 | 27 | *Nat. Commun.* **11**, 1278 (2020) |
| Ru@C_2_N | 1 M KOH; glassy carbon | 5 | 17 | 38 | *Nat. Nanotechnol.* **12**, 441-446 (2017) |
| Bped-Pt/GR | 1 M KOH; glassy carbon | 5 | 18 | 46.9 | *Angew. Chem. Int. Ed.* **58**, 19060-19066 (2019) |
| Au-Ru-2 NWs | 1 M KOH; glassy carbon | 2 | 25 | 15.6 | *Nat. Chem.* **10**, 456–461 (2018) |
| Pt-NC/Ni-MOF | 1 M KOH; glassy carbon | 5 | 25 | 42.1 | *Chem* **5**, 2429-2441 (2019) |
| RuCo@NC | 1 M KOH; glassy carbon | 2 | 28 | 31 | *Nat. Commun.* **8**, 14969 (2017) |
| a-RuTe_2_ PNRs | 1 M KOH; glassy carbon | N/A | 36 | 36 | *Nat. Commun.* **10**, 5692 (2019) |
| PtNi-O/C | 1 M KOH; glassy carbon | 5 | 39.8 | 78.8 | *J. Am. Chem. Soc.* **140**, 9046-9050 (2018) |
| RhPd-H | 1 M KOH; glassy carbon | 5 | 40 | 35.7 | *J. Am. Chem. Soc.* **142**, 3645-3651 (2020) |
| Pt_3_Ni_2_-NWs-S/C | 1 M KOH; glassy carbon | 10 | 42 | N/A | *Nat. Commun.* **8**, 14580 (2017) |
| Pt/Ni(HCO_3_)_2_ | 1 M KOH; glassy carbon | 5 | 44 | 40 | *Angew. Chem. Int. Ed.* **58**, 5432-5437 (2019) |
| Pt_1_/N-C | 1 M KOH; glassy carbon | 5 | 46 | 36.8 | *Nat. Commun.* **11**, 1029 (2020) |
| Pt_5_/HMCS | 1 M KOH; glassy carbon | 1 | 46.2 | 48.1 | *Adv. Mater.* **32**, 1901349 (2020) |
| Ru-NC-700 nanowires | 0.1 M KOH; glassy carbon | N/A | 47 | 14 | *Nat. Commun.* **10**, 631 (2019) |
| Ni-branched Pt-islands | 0.1 M KOH; glassy carbon | 10 | 49 | 69 | *J. Am. Chem. Soc.* **141**, 16202-16207 (2019) |
| Ir_1_@Co/NC | 1 M KOH; glassy carbon | 10 | 55 | 119 | *Angew. Chem. Int. Ed.* **58**, 11868-11873 (2019) |
| Sr_2_RuO_4_ | 1 M KOH; glassy carbon | 5 | 61 | 51 | *Nat. Commun.* **10**, 149 (2019) |
| hcp Pt-Ni nano-multipods | 0.1 M KOH; glassy carbon | 10 | 65 | 78 | *Nat. Commun.* **8**, 15131 (2017) |
| SANi-PtNWs | 1 M KOH; glassy carbon | 5 | ca. 70 | 60.3 | *Nat. Catal.* **2**, 495-503 (2019) |

**Table R5 and revised Supplementary Table 4.** Comparison of OER activities with various recently reported state-of-the-art catalysts in neutral electrolyte.

| *Catalyst* | *Medium and electrode* | *Scan rate (mV s^-1^)* | *η @ 10 mA cm^-2^ (mV)* | *Tafel slope (mV dec^-1^)* | *Reference* |
| --- | --- | --- | --- | --- | --- |
| Ir-NSG | 1 M PBS; glassy carbon | 1 | 307 | 74.2 | This work |
|  |  | 5 | 297 |  |  |
| Li-IrSe_2_ | 1 M PBS; carbon fiber paper | 2 | 315 | N/A | *Angew. Chem. Int. Ed.* **58**, 14764-14769 (2019) |
| NiCoFeP | 0.5 M KHCO_3_; Au-plated Ni foam | 1 | 330 | 60 | *Nat. Chem.* **10**, 149–154 (2017) |
| Co_3_(PO4)_2_ nanosheets | 0.1 M PBS; glassy carbon | 5 | 360 | 70 | *Angew. Chem. Int. Ed.* **58**, 14599-14604 (2019) |
| Ni-Fe-Mg | 0.5 M KHCO_3_; Ni foam | 1 | 360 | 150 | *Adv. Mater.* **32**, 1906806 (2020) |
| CoIr-0.2 | 1 M PBS; glassy carbon | 10 | 373 | 117.5 | *Adv. Mater.* **30**, 1707522 (2018) |
| Co-Pi NA/Ti | 1 M PBS; Ti mesh | 5 | 380 | 187 | *Angew. Chem. Int. Ed.* **56**, 1064-1068 (2017) |
| CoMoNiS-NF-31 | 1 M PBS; Ni foam | 5 | 405 | 71 | *J. Am. Chem. Soc.* **141**, 10417-10430 (2019) |
| NiFe_2_O_4_/FeNi_2_S_4_ HNSs | 0.2 M PBS; carbon fiber paper | 2 | 429 | N/A | *J. Am. Chem. Soc.* **140**, 17624-17631 (2018) |
| Karst NF | 1 M PBS; Ni foam | 5 | 432 | 249 | *Energy Environ. Sci.* **13**, 174-182 (2020) |
| IrO_2_ | 0.5 M NaHCO_3_; Ti foil | 10 | 460 | N/A | *Nat. Commun.* **6**, 7326 (2015) |
| Co4Mo | 0.1 M PBS; glassy carbon | 10 | 490 | 144 | *Angew. Chem. Int. Ed.* **131**, 145-149 (2019) |
| Co-Bi NS/G | 1 M PBS; glassy carbon | 5 | ca. 500 | 160 | *Angew. Chem. Int. Ed.* **55**, 2488-2492 (2016) |
| 1@Fe_3_O_4_ NR | 0.1 M PBS; Ti mesh | 5 | 510 | 61.4 | *Angew. Chem. Int. Ed.* **58**, 18883-18887 (2019) |
| Ni_0.1_Co_0.9_P | 1 M PBS; carbon fiber paper | 5 | 550 | 133 | *Angew. Chem. Int. Ed.* **130**, 15671-15675 (2018) |
| LT-LiCoO_2_ | 1 M PBS; glassy carbon | 5 | 570 | 75 | *Energy Environ. Sci.* **9**, 184-192 (2016) |
| p-FGDY | 1 M PBS; carbon cloth | 1 | ca. 700 | 330 | *Angew. Chem. Int. Ed.* **58**, 13897-13903 (2019) |

**Table R6 and revised Supplementary Table 5.** Comparison of OER activities with various recently reported state-of-the-art noble metal-based catalysts in acidic electrolyte.

| *Catalyst* | *Medium and electrode* | *Scan rate (mV s^-1^)* | *η @ 10 mA cm^-2^ (mV)* | *Tafel slope (mV dec^-1^)* | *Reference* |
| --- | --- | --- | --- | --- | --- |
| Ir-NSG | 0.1 M HClO_4_; glassy carbon | 1 | 265 | 44.2 | This work |
|  |  | 5 | 255 |  |  |
| Amorphous Ir nanosheets | 0.1 M HClO_4_; glassy carbon | 5 | 255 | 40 | *Nat. Commun.* **10**, 4855 (2019) |
| Ru-N-C | 0.5 M H_2_SO_4_; glassy carbon | 5 | 267 | 52.6 | *Nat. Commun.* **10**, 4849 (2019) |
| IrO_x_/SrIrO_3_ | 0.5 M H_2_SO_4_; SrIrO_3_ film | 10 | 270 | 40 | *Science* **353**, 1011-1014 (2016) |
| 40-IG | 0.5 M H_2_SO_4_; glassy carbon | 5 | 278 | 57 | *Angew. Chem. Int. Ed.* **58**, 12540-12544 (2019) |
| Ru@IrO_x_ | 0.05 M H_2_SO_4_; gold electrode | 5 | 282 | 69.1 | *Chem* **5**, 445-459 (2019) |
| Pr_2_Ir_2_O_7_ | 0.1 M HClO_4_; glassy carbon | 5 | 290 | N/A | *Adv. Mater.* **31**, 1805104 (2019) |
| IrO_x_-Ir | 0.5 M H_2_SO_4_; glassy carbon | 5 | 290 | 43.5 | *Angew. Chem. Int. Ed.* **128**, 752-756 (2016) |
| Dtf-IrOs | 0.1 M HClO_4_; glassy carbon | 10 | ca. 290 | N/A | *Nat. Commun.* **8**, 1449 (2017) |
| Li-IrO_x_ | 0.5 M H_2_SO_4_; glassy carbon | 1 | 300 | 39 | *J. Am. Chem. Soc.* **141**, 3014-3023 (2019) |
| IrCoNi PHNC | 0.1 M HClO_4_; glassy carbon | 5 | 303 | 53.8 | *Adv. Mater.* **29**, 1703798 (2017) |
| IrW/C | 0.1 M HClO_4_; glassy carbon | 5 | ca. 310 | 56.6 | *ACS Cent. Sci.* **4**, 1244-1252 (2018) |
| Ba_2_YIrO_6_ | 0.1 M HClO_4_; gold electrode | 10 | ca. 330 | 67 | *Nat. Commun.* **7**, 12363 (2016) |
| N-HC@G-900 | 0.5 M H_2_SO_4_; glassy carbon | 5 | 350 | 88.1 | *Angew. Chem. Int. Ed.* **130**, 16749-16753 (2018) |
| W_0.57_Ir_0.43_O_3-σ_ | 1 M H_2_SO_4_; glassy carbon | 20 | 370 | 125 | *Energy Environ. Sci.* **10**, 2432-2440 (2017) |
| Sr_2_CoIrO_6_ | 0.1 M HClO_4_; glassy carbon | 10 | ca. 370 | N/A | *Angew. Chem. Int. Ed.* **131**, 4619-4623 (2019) |
| Rh_2_P/C | 0.5 M H_2_SO_4_; glassy carbon | 20 | 550 | N/A | *J. Am. Chem. Soc.* **139**, 5494-5502 (2017) |

**Fig. R5.** Chronopotentiometric curves of Ir-NSG coupled water electrolysis cell at 10 mA cm^-2^ in **a,** 1 M PBS, **b,** 0.1 M HClO_4_, and **c,** 1 M KOH. All measurements were conducted in Ar-saturated electrolytes without *i*-R drop correction.

**Table R1 and revised Supplementary Table 6.** Comparison of overall-water-splitting activities and stabilities with various recently reported state-of-the-art catalysts in acidic electrolyte.

| *Catalyst* | *Medium and electrode* | *Cell Voltage (V)* | *Durability @ 10 mA cm^-2^ (h)* | *Reference* |
| --- | --- | --- | --- | --- |
| Ir-NSG | 0.1 M HClO_4_; carbon fiber paper | 1.42 | 24 h @ 10 mA cm^-2^ | This work |
| Li-IrSe_2_ | 0.5 M H_2_SO_4_; carbon fiber paper | 1.44 | 24 h @ 1.47 V | *Angew. Chem. Int. Ed.* **58**, 14764-14769 (2019) |
| CoMoNiS-NF-31 | 0.5 M H_2_SO_4_; carbon fiber paper | 1.45 | 80 min @ 1.53 V | *J. Am. Chem. Soc.* **141**, 10417-10430 (2019) |
| IrW/C | 0.5 M H_2_SO_4_; carbon fiber paper | 1.45 | 8 h @ 10 mA cm^-2^ | *ACS Cent. Sci.* **4**, 1244-1252 (2018) |
| RuIrO_x_ | 0.5 M H_2_SO_4_; carbon fiber paper | 1.45 | 24 h @ 10 mA cm^-2^ | *Nat. Commun.* **10**, 4875 (2019) |
| RuCu NCs | 0.5 M H_2_SO_4_; N/A | 1.49 | 15 h @ 10 mA cm^-2^ | *Angew. Chem. Int. Ed.* **58**, 13983-13988 (2019) |
| Co-RuIr | 0.1 M HClO_4_; N/A | 1.52 | 25 h @ 10 mA cm^-2^ | *Adv. Mater.* **31**, 1900510 (2019) |
| a-RuTe_2_ PNRs | 0.5 M H_2_SO_4_; N/A | 1.52 | 24 h @ 7 mA cm^-2^ | *Nat. Commun.* **10**, 5692 (2019) |
| IrCoNi PHNC | 0.1 M HClO_4_; carbon fiber paper | ca. 1.66 | ca. 3 h @ 5 mA cm^-2^ | *Adv. Mater.* **29**, 1703798 (2017) |
| p-FGDY | 0.5 M H_2_SO_4_; carbon cloth | 1.80 | 5 h @ 10 mA cm^-2^ | *Angew. Chem. Int. Ed.* **58**, 13897-13903 (2019) |

**Fig. R13. Comparisons of HER and OER performance. a,** Comparison of overpotential (@ 10 mA cm^-2^) and Tafel slope for various state-of-the-art HER catalysts in neutral medium. **b,** Comparison of overpotential (@ 10 mA cm^-2^) and Tafel slope for various state-of-the-art OER catalysts in neutral medium. **c,** Comparisons of overpotential (@ 10 mA cm^-2^) and Tafel slope for various state-of-the-art noble metal-based HER catalysts in acidic medium. **d,** Comparisons of overpotential (@ 10 mA cm^-2^) and Tafel slope for various state-of-the-art noble metal-based OER catalysts in acidic medium. **e,** Comparisons of overpotential (@ 10 mA cm^-2^) and Tafel slope for various state-of-the-art noble metal-based HER catalysts in alkaline medium. The overpotential of Ir-NSG was determined from LSV curve obtained at a scan rate of 5 mV s^-1^ and other values were plotted from references in Tables R2, R5, R3, R6 and R4 for **a**, **b**, **c**, **d** and **e**, respectively.

Correspondingly, we have added Tables R2, R3, R4, R5, R6 and R1 as Supplementary Tables 1, 2, 3, 4, 5 and 6 in the revised Supplementary Information, respectively. Figs. R13a, b, c, d and e have also been added as Figs. 2d, 2h, Supplementary Figs. 18d, 20d and 19d in the revised manuscript and Supplementary Information, respectively.

**Responses to the remarks of Reviewer #3:**

*Wang and collaborator synthesized iridium nanoclusters coordinated with both nitrogen and sulfur and supported on nitrogen and sulfur co-doped graphene, and showed that the system can serve as a bifunctional HER and OER pH-universal catalyst. This contributes an important finding. The study employs a variety of experimental measurements and theoretical modeling based on DFT to understand the mechanism. Thus, the approach is of interest to the wider community. The paper is well written. While I find the study interesting especially the collaborative investigations at the atomic level, I had several concerns to have a convincing story. The SI can benefit from more details about the experiment and theory, and not just figures with captions.*

**Response:** We thank the reviewer for the positive comment and evaluating our work as “an important finding and well-written paper”. According to the reviewer’s suggestion, we have added Supplementary Notes 1 and 2 about the mechanism of determining HBE by CV method and adsorption energy of OH* by methanol oxidation reaction in the revised Supplementary Information, respectively, as shown in Notes R1 and R2. Moreover, we have added more details about experiments and DFT calculations in the revised manuscript and Supplementary Information.

**Note R1 and revised Supplementary Note 1.** **Determination of hydrogen binding energy (HBE) from underpotentially deposited hydrogen (H_upd_) using cyclic voltammogram (CV) method**

Hydrogen binding energy (HBE) on metal surface Δ*H* is defined by the enthalpy change of dissociative adsorption of H_2_ (Sheng, W. et al. *Nat. Commun.* 2015, **6**, 5848).

$\frac{\text{1}}{\text{2}}\text{H}_{\text{2}}\text{ + * = }\text{H}^{\text{*}}$	(1)

Since this is a simplified model to compare HBEs qualitatively, we assume H adsorption obeys Langmuir isotherm with only considering the configurational entropy (Karlberg, G. S. et al. *Phys. Rev. Lett.* 2007, **99**, 126101). Thus, for Rxn (1) at temperature *T*,

$\text{Δ}\text{G}_{\text{1}}\text{ = Δ}\text{H}\text{ }-\text{ }\text{T}\text{Δ}\text{S}_{\text{conf}}\text{ = Δ}\text{H}\text{ }-\text{ }\text{k}_{\text{b}}\text{T}\text{ln}\frac{\text{1-}\text{θ}}{\text{θ}}$	(2)

where *k*_b_ is the Boltzmann constant and *θ* is the coverage of H.

As the adsorption/desorption process of H can be evaluated via

$\text{H}^{\text{+}}\text{ +}{\text{ }\text{e}}^{\text{-}}\text{ + * = }\text{H}^{\text{*}}$	(3)

Rxn (3) – Rxn (1) can give the overall reaction of HER

$\text{H}^{\text{+}}\text{ + }\text{e}^{\text{-}}\text{ = }\frac{\text{1}}{\text{2}}\text{H}_{\text{2}}$	(4)

The Gibbs free energy (${\text{∆}\text{G}}_{\text{4}}^{\text{o}}$) of Rxn (4) is zero at a potential of 0 V under standard conditions (*T* = 298 K, $\text{p}_{\text{H}_{\text{2}}}\text{ }\text{=}\text{ }\text{1 atm}$ and *p*H = 0).

Then the correlation between HBEs and the H_upd_ peak potentials can be derived in CVs assuming adsorption/desorption equilibrium throughout the measurements (Zheng, J. et al. *ACS Catal.* 2015, **5**, 4449-4455).

Following the computational hydrogen electrode (CHE) model by Nørskov et al., one can assume that Δ*G*(H^+^ + *e*^-^) is equal to −*e*_0_*E* at any potential *E* (vs. standard hydrogen electrode (SHE)), which circumvents the explicit treatment of solvated proton in electrolyte solution (Nørskov, J. K. et al. *J. Phys. Chem. B* 2004, **108**, 17886-17892).

For Rxn (3), at potential *E*_3_ (vs. SHE), Δ*G*_3_ = −*FE*_3_, where *F* is the Faraday constant.

Since $\text{E}_{\text{RHE}}\text{ =}{\text{ }\text{E}}_{\text{SHE}} - \frac{\text{RT}\text{ln}\left[ \text{H}^{\text{+}} \right]}{\text{F}}\text{ =}{\text{ }\text{E}}_{\text{SHE}}\text{ + }\frac{\text{2.303}\text{RT}\left( \text{p}\text{H} \right)}{\text{F}}$,

$\text{E}\text{ =}{\text{ }\text{E}}_{\text{3}}\text{ + }\frac{\text{2.303}\text{RT}\text{(}\text{p}\text{H)}}{\text{F}}\text{ = }-\frac{\text{∆}\text{G}_{\text{3}}}{\text{F}}\text{ + }\frac{\text{2.303}\text{RT}\text{(}\text{p}\text{H)}}{\text{F}}$	(5)

where *E* is on the reversible hydrogen electrode (RHE) scale.

Under reaction conditions (*T* = 298 K, $\text{p}_{\text{H}_{\text{2}}}\text{ = 1 atm}$ and different $\text{a}_{\text{H}^{\text{+}}}$),

$$\text{∆}\text{G}_{\text{4}}\text{ = }{\text{∆}\text{G}}_{\text{4}}^{\text{o}}\text{ + }\text{RT}\text{ln}\frac{\text{p}_{\text{H}_{\text{2}}}^{\text{1/2}}}{\text{a}_{\text{H}^{\text{+}}}}\text{ = 2.303}\text{RT}\text{(}\text{p}\text{H)}$$

Because Δ*G*_4_ = Δ*G*_3_ – Δ*G*_1_,

Δ*G*_3_ = Δ*G*_1_ + 2.303*RT*(*p*H)	(6)

Substitute Eqs (2) and (6) into Eq (5),

$\text{E}\text{ = }-\frac{\text{∆}\text{H}}{\text{F}}\text{ + }\frac{\text{k}_{\text{b}}\text{T}}{\text{F}}\text{ln}\frac{\text{1}-\text{θ}}{\text{θ}}$	(7)

Differentiate Eq (7) with respect to *θ*,

$\frac{\text{d}\text{E}}{\text{d}\text{θ}}\text{ = }-\frac{\text{k}_{\text{b}}\text{T}}{\text{F}}\frac{\text{1}}{\text{θ}\text{(1}-\text{θ}\text{)}}$	(8)

Assuming transfer of one electron per adsorbed H, the relationship between the charge transferred per area *Q* and the coverage *θ* is

*Q* = *Q*_tot_*θ*	(9)

where *Q*_tot_ is *e* times of the densities of active sites in the surface layer (Karlberg, G. S. et al. *Phys. Rev. Lett.* 2007, **99**, 126101).

During CVs, the relationship between current density *i(t)* and the coverage *θ* is as follows:

$\text{i}\text{(}\text{t}\text{) = ±}\frac{\text{d}\text{Q}}{\text{d}\text{t}}\text{ = ±}\text{Q}_{\text{tot}}\frac{\text{d}\text{θ}}{\text{d}\text{t}}$	(10)

Substitute Eq (8) into Eq (10),

$\text{i}\text{(}\text{t}\text{) = ±}\text{Q}_{\text{tot}}\frac{\text{d}\text{θ}}{\text{d}\text{t}}\text{ = ±}\text{Q}_{\text{tot}}\frac{\text{d}\text{θ}}{\text{d}\text{E}}\frac{\text{d}\text{E}}{\text{d}\text{t}}\text{ = ±}\text{Q}_{\text{tot}}\frac{\text{d}\text{E}}{\text{d}\text{t}}\frac{\text{F}}{\text{k}_{\text{b}}\text{t}}\text{θ}\text{(1}-\text{θ}\text{)}$	(11)

where $\text{±}\frac{\text{d}\text{E}}{\text{d}\text{t}}$ is the potential sweep rate.

At peak potential, current density *i(t)* reaches the maximum value, so d*i(t)*/d*θ* will be 0. In Eq (11), when d*i(t)*/d*θ* = 0, *θ* is 0.5.

In Eq (7), at *θ* = 0.5,

$\text{E}_{\text{peak}}\text{ = }-\frac{\text{∆}\text{H}}{\text{F}}\text{ + }\frac{\text{k}_{\text{b}}\text{T}}{\text{F}}\text{ln}\frac{\text{1}-\text{θ}}{\text{θ}}\text{ = }-\frac{\text{∆}\text{H}}{\text{F}}$	(12)

Therefore, a direct link between the HBEs on metal surfaces and experimental CVs has been built by Eq (12).

**Note R2 and revised Supplementary Note 2. Chemical principle of probing adsorption energy of OH* with methanol oxidation reaction (MOR)**

OH* adsorbed on metal catalysts is an essential species in electrooxidation of methanol as well as the first intermediate for OER catalytic cycle (Zope, B. N. et al. *Science* 2010, **330**, 74-78; Reier, T. et al. *Adv. Energy Mater.* 2017, **7**, 1601275). A method of using alcohol molecules to probe reactive OH* generated in OER has been proposed recently (Tao, H. B. et al. *Joule* 2019, **3**, 1498-1509). The reaction between methanol and surface adsorbed OH* takes place very fast at low potentials, while its selectivity declines after the raise of OER because of the faster deprotonation of OH* and favourable kinetics of OER than MOR at high potentials (Rodriguez, P. et al. *Nat. Chem.* 2012, **4**, 177-182).

The detailed explanation of this mechanism is discussed as follows:

CH_2_H_β_-OH_α_ + OH^−^ ↔ CH_2_H_β_-O^−^ + H_α_OH	(13)

where OH^−^ is present in base and from water dissociation in neutral or acidic media (α and β indicate the protons bonded at O and C, respectively).

M* + OH^−^ ↔ M-OH* + *e*^−^	(14)

This is the first step in OER cycle, M* refers to the active site.

CH_2_H_β_-O^−^ + M-OH* → CH_2_=O + H_β_OH + M* + *e*^−^	(15)

The electrophilic OH* is nucleophilically attacked by CH_2_H_β_-O^−^, which loses one electron to form formaldehyde.

CH_2_=O + M-OH* + OH^−^ → HCOO^−^ + H_2_O + M*	(16)

Then formaldehyde can be oxidized to formate with the assistance of OH*.

From the mechanism presented above, it can be seen that adsorption energy of OH* can be probed by MOR qualitatively. If adsorption energy of OH* on active sites is too low in Eq (14), the catalyst surface will be free of OH*, and thus there is no response to methanol oxidation. However, when the adsorption energy of OH* is large enough, OH* will be accumulated on the surface of catalyst, which will contribute to MOR. Generally, lower onset potential of MOR means higher surface coverage of OH* as well as higher adsorption energy of OH*.

In addition, we also provide more computational details about free energy calculations in the revised manuscript (at lines of 493-503).

To derive the reaction free energy (∆*G*), differences in zero-point energy (∆ZPE) and entropy effects are taken into account as follows: ∆*G* = ∆*E* + ∆ZPE – *T*∆*S*, where ∆(*PV*) = 0 for the solution system. The reaction energy ∆*E* is available from quantum mechanical calculations (e.g., DFT) and change in entropy (∆*S*) at *T* = 298.15 K is obtained from vibrational frequency calculations. Free energies are used for all absorbed species (Nørskov, J. K. et al. *J. Electrochem. Soc.* 2005, **152**, J23-J26; Rossmeisl, J. et al. *J. Electroanal. Chem.* 2007, **607**, 83-89), while the corrections for gas phase molecules are taken from standard thermodynamics tables (Atkins, P. W. *Physical Chemistry* 6th edn Oxford Univ. Press, 1998). The standard hydrogen electrode (SHE) is used as the reference electrode to define the potential. The free energy change of 1/2H_2_ → H^+^ + *e*^−^ reaction will be zero at the potential of 0 V and 1/2*G*(H_2_) is used to represent the free energy of proton and electron. The free energy of gaseous O_2_ is derived as *G*(O_2_) = 2*G*(H_2_O) – 2*G*(H_2_) + 4.92 (eV) (Fang, Y.-H. et al. *J. Am. Chem. Soc.* 2010, **132**, 18214-18222). The free energy at an applied overpotential U is calculated as *G*_U_ = *G* – *ne*U, where *n* is the number of (H^+^ + *e*^−^) pairs involved and *e* is the transferred electron.

**Comment 1:** *It is not clear what is the role of N/S in the system based on experimental measurements? Do these improve the catalytic activity?*

**Response 1:** We are grateful to the reviewer for raising this interesting and meaningful question. We realize that the original experimental results cannot thoroughly elucidate how the N and S dopants synergistically influence the activities of Ir. To fully clarify the role of N and S, we synthesized N, S-doped graphene (NSG) and Ir nanoclusters on N-doped graphene (Ir-NG) as control samples in this revision. On the basis of these additional experimental results, we conclude that the Ir sites coordinated with both N and S are responsible for the enhanced activities of Ir-NSG. The detailed interpretations are presented below:

We synthesized pristine NSG without adding iridium(III) chloride hydrate and the control sample of Ir-NG using L-alanine (C_3_H_7_NO_2_) in place of L-cysteine (C_3_H_7_NO_2_S). The mesoporous and sheet-like structure of NSG is consistent with that of Ir-NSG (Fig. R7). Moreover, as revealed by the BF TEM image and STEM-EDX elemental mappings of Ir-NG, the Ir nanoclusters are homogeneously distributed in the N-doped graphene framework (Fig. R8). The mass content of Ir in Ir-NG determined by ICP-AES is 7.07 wt. %, which is close to that of Ir-NSG. The chemical composition and valence states at the surface of Ir-NG were further examined by XPS (Fig. R9). The high-resolution XPS N 1*s* spectrum could be deconvoluted into Ir-N, pyridinic, pyrrolic, graphitic and oxidized N species, respectively (Fig. R9b), and the N of Ir-N is in the pyridinic form (Yang, H. B. et al. *Nat. Energy* 2018, **3,** 140-147). Intriguingly, the corresponding binding energy of Ir for Ir-NG is lower than that for IrO_2_ but higher than that for Ir-NSG as highlighted by the dash line, in line with the medium electronegativity of N comparing with that of O and S (Fig. R9c).

To verify the individual role of N/S coordination in the electrocatalytic reactions, we added the HER and OER measurements of NSG and Ir-NG. Fig. R10 shows the HER and OER polarization curves of Ir-NSG in comparison with NSG, Ir-NG and benchmark Pt/C and Ir/C in neutral, acidic and alkaline electrolytes. It can be found that the HER and OER activities of Ir-NSG are dramatically higher than those of pristine NSG, indicating that Ir can effectively boost HER and OER while N and S are not the active sites. Furthermore, it should also be noted that although Ir-NG can enhance the HER and OER activities, they are still far inferior to those of Ir-NSG.

**Fig. R7 and revised Supplementary Figure 15.** Typical SEM images of NSG, showing the same morphology with Ir-NSG. Scale bars are 3 μm and 300 nm for **a** and **b**, respectively.

**Fig. R8 and revised Supplementary Figure 16. Morphology of Ir-NG. a,** Bright-field TEM image of Ir-NG (with 7.07 wt. %), showing that Ir nanoclusters are homogeneously distributed in the N-doped graphene framework. Scale bar: 20 nm. **b,** Dark-field STEM image and EDX elemental mappings, scale bars: 5 nm.

**Fig. R9 and revised Supplementary Figure 17. XPS spectra of Ir-NG. a,** Survey spectrum of Ir-NG, revealing the absence of S (shadow area) and successful doping of N into graphene. **b,** High-resolution XPS N 1*s* spectrum, confirming the presence of Ir-N coordination in the pyridinic form. **c,** High-resolution XPS Ir 4*f* spectra. The corresponding binding energy of Ir for Ir-NG is between that for Ir-NSG and IrO_2_ as highlighted by the dash line, in accordance with the medium electronegativity of N comparing to that of O and S.

**Fig. R10. HER and OER performance of the Ir-NSG catalyst in comparison with NSG and Ir-NG. a,** HER polarization curves acquired in Ar-saturated 1 M PBS. **b,** OER polarization curves acquired in 1 M PBS. **c,** HER polarization curves acquired in Ar-saturated 0.1 M HClO_4_. **d,** OER polarization curves acquired in 0.1 M HClO_4_. **e,** HER polarization curves acquired in Ar-saturated 1 M KOH. **f,** OER polarization curves acquired in 1 M KOH. All measurements were calibrated with *i*R-compensation. Catalyst loading: 0.3 mg cm^-2^. Rotation speed: 1,600 rpm. The LSV curves were obtained at a scan rate of 1 mV s^-1^.

Correspondingly, we have added the Figs. R7, R8 and R9 as Supplementary Figs. 15, 16 and 17 in the revised Supplementary Information, respectively. Moreover, Figs R10a, b, c, d, e, f have been added as Figs. 2a, 2e and Supplementary Figs. 18a, 20a, 19a and 21a in the revised manuscript and Supplementary Information. We have also added the statement of “From the polarization curves of HER and OER, it can be found that Ir-NSG can effectively boost both HER and OER in comparison with pristine NSG. Moreover, although Ir-NG can enhance the activities of HER and OER, they are still far inferior to those of Ir-NSG. All these results imply that Ir coordinated with both N and S could be the real active site in Ir-NSG.” in the lines of 262-266 in the revised manuscript.

**Comment 2:** *What is the experimental motivation for introducing the DFT models and the Ir-N-S coordination? Can the authors use the DFT models to validate the experimental measurements? Showing that these particular models yield good HER/OER is not sufficient. Experimentally supplementary Figure 7 shows an oxidized state of Ir. Why oxygen adatoms are not included in the models for HER? Given the low overpotential in the system, oxygen adsorbates could be stabilized.*

**Response 2:** We thank the reviewer for the useful comments. From the results of STEM-EDX mappings, XPS and XAFS, we have concluded that there are Ir-N and Ir-S coordination in Ir-NSG. Moreover, based on HER and OER experiments, we observed outstanding electrocatalytic performance of Ir-NSG. Thus, we introduced DFT model calculations (the DFT models were constructed based on Ir sites coordinated with both N and S, with two N atoms and one S atom) to understand the coordination environment of the active sites and the nature of reaction mechanism at the atomic scale for HER and OER on Ir-NSG. As a conclusion, DFT calculations support the finding that Ir sites coordinated with both N and S are active for both HER and OER, and the obtained corresponding energy profiles are also consistent with the experimental results (HUPD and MOR measurements) as stated in the Discussion Section in the revised Manuscript.

Moreover, to address the reviewer’s concern for the variety of DFT models, we have added DFT model of Ir-NG to assess the individual role of N coordination. The results are in line with the control experiments as stated in Response 1 (Fig. R10). The free-energy diagrams of Ir-NG for HER and OER shown in Figs. R11 and R12 all indicate that Ir site of Ir-NSG is far more active than that of Ir-NG, as Ir-NG shows higher energy barriers (0.37 eV for HER and 0.75 eV for OER, respectively).

**Fig. R11.** DFT predicted free-energy diagrams for HER at U = 0 V on Ir-NG (cyan, magenta and orange lines), in comparison with Ir-NSG (red line). Hydrogen adsorption configurations on different Ir sites were considered, where the most favorable Ir site is shown in cyan circle.

**Fig. R12.** DFT predicted free-energy diagrams for OER at U = 1.23 V on Ir-NG (cyan line), compared with Ir-NSG (red line).

In fact, the oxidized state of Ir is dependent on the coordination with N and S, as there is no O atom at the first coordination shell. Maybe we did not express clearly enough that seems to cause some confusions here: the label of Ir O_2, 3_ in Fig. R14 (Supplementary Fig. 7) means the fifth electron shell (O shell) of Ir atom instead of adsorbed oxygen atom. So we have added the statement of “The labels of Ir N_6, 7_ and Ir O_2, 3_ represent the fourth and fifth electron shell of Ir atom, respectively” as details in the caption of Supplementary Fig. 7 in the revised Supplementary Information. Moreover, Ir-NSG cannot be further oxidized or adsorb oxygen atom during HER (detailed discussion is shown in Fig. R15 and Response 3), thus oxygen adatoms are not included in the DFT models for HER.

**Fig. R14 and revised Supplementary Figure 7.** Power-law background subtracted EELS spectrum of Ir-NSG. The labels of Ir N_6, 7_ and Ir O_2, 3_ represent the fourth and fifth electron shell of Ir atom, respectively.

**Comment 3:** *Did the authors assess the long-term stability of the catalyst and whether this is accompanied by any variations in the structure of the catalyst?*

**Response 3:** We thank the reviewer for raising this important question. We have tested the long-term stabilities of Ir-NSG for overall water splitting at 10 mA cm^-2^ in a two-electrode water electrolysis cell in 1 M PBS, 0.1 M HClO_4_ and 1 M KOH solution, respectively. As shown in Fig. R5, there are slight degradations over a 24-hour operation, which are among the most stable overall-water-splitting catalysts reported to date. Moreover, the morphology and structure of Ir-NSG were examined by TEM and XPS after long-term HER and OER. As shown in the TEM images (Figs. R15a and b), Ir-NSG catalyst keeps uniformly dispersed nanoclusters without aggregation after undergoing long-term HER and OER. Moreover, as shown in Fig. R15c, the XPS result demonstrates that Ir in Ir-NSG maintains its original valence state without being oxidized during HER. However, on the other hand, Ir in Ir-NSG was further oxidized to an oxidation state > +4 after OER test, in accordance with the results of *in-situ* XAFS measurements in the manuscript.

**Fig. R5.** Chronopotentiometric curves of Ir-NSG coupled water electrolysis cell at 10 mA cm^-2^ in **a,** 1 M PBS, **b,** 0.1 M HClO_4_, and **c,** 1 M KOH. All measurements were conducted in Ar-saturated electrolytes and without *i*-R drop correction.

**Fig. R15 and revised Supplementary Figure 32.** **Structure of Ir-NSG after long-term stability test. a,** Bright-field TEM image of Ir-NSG after HER test, scale bar: 20 nm. **b,** Bright-field TEM image of Ir-NSG after OER test, scale bar: 20 nm. As shown in **a** and **b**, the Ir-NSG catalyst keeps uniformly dispersed nanoclusters without aggregation after undergoing long-term HER and OER. **c,** High-resolution XPS Ir 4*f* spectra. The corresponding binding energy of Ir highlighted by the dash line reveals that the valence state of Ir keeps almost constant during HER (green dash line) while becoming higher than that of IrO_2_ after OER (blue dash line), which is in accordance with the results of *in-situ* XAFS measurements.

Accordingly, we have added the Figs. R5a, b, c as insets in Figs. 4b, c, d in the revised manuscript. Fig. R15 has also been added as Supplementary Fig. 32 in the revised Supplementary Information. And the corresponding statements in lines 341-345 of the manuscript have been revised as “Insets in Figs. 4b, c, and d manifest that the electrolyzer assembled with Ir-NSG anode and cathode can retain a current density of 10 mA cm^-2^ over long-term continuous tests with slight voltage decay and no detectable structure degradation at all pH values (Supplementary Fig. 32), which is among the most robust overall-water-splitting catalysts reported to date, especially in acidic media (Supplementary Table 6).”.

**Comment 4:** *In Figure 3a, the HBE for Ir in alkaline solution is listed as Ir (111) as well as Pt (110) and Pt (100). In operando X-ray absorption spectroscopy, I think only the Ir-NSG and Pt/C can be measured as a whole instead of each termination. This needs more elaboration.*

**Response 4:** We thank the reviewer for the comments. The HBEs for Ir-NSG and Pt/C were determined by the CV method instead of *operando* XAS (Note R1), which has been demonstrated useful to correlate HBE with the corresponding metal facet via H_upd_ desorption peak (Durst, J. et al. *Energy Environ. Sci.* 2014, **7**, 2255-2260). As shown in Fig. R16 (revised Supplementary Fig. 22c), HBEs are determined to be -0.121 and -0.212 eV for Ir(111) and Ir(200), while -0.255 and -0.356 eV for Pt(110) and Pt(100) in alkaline solution, respectively (Zheng, J. et al. *ACS Catal.* 2015, **5,** 4449-4455). Additionally, the peaks above ~0.6 V vs. RHE are related to OH^−^ anion adsorption/desorption (Sheng, W. et al. *Nat. Commun.* 2015, **6,** 5848). H_upd_ desorption region is chosen for analysis because the H_upd_ adsorption process is susceptible to the effect of pre-adsorbed species whereas the H_upd_ desorption process remains essentially stable (Zheng, J. et al. *ACS Catal.* 2015, **5,** 4449-4455).

**Fig. R16.** Underpotentially deposited hydrogen of the Ir-NSG catalyst and commercial Pt/C in 1 M KOH. Catalyst loading: 0.3 mg cm^-2^. All the measurements were operated in Ar-saturated electrolytes with *i*R-compensation. The dashed lines serve as the eye guide for comparison.

**Comment 5:** *I have strong concerns about the DFT modeling and the approach that I feel is too simplistic. Bulk Ir without N/S has already excellent activity without the need for N/S. Also, from Figure S21, I see that there are different adsorption sites with different adsorption strength. It is clearly shown that the first adsorption site (with dG = -0.04 eV) is the best because dG is very close to zero. However, there are several strong adsorption sites with dG from -0.4 to -0.6 eV, and the adsorption sites with dG around -0.3 eV cannot be even considered as the high activity sites for the large current as shown in cyclic voltammogram. I believe the adsorbed hydrogen will fast diffuse to these strong adsorption sites before waiting for another hydrogen atom to be adsorbed to the nearby adsorption sites to form H_2_ through the Tafel reaction. The way to test the dG for these adsorption sites is to understand the most stable adsorption configurations at experimental conditions (different pH and at 300 K). This is because the H-H interactions are also a factor for dG. (Chem. Mater. 2020, 32, 1315−1321) To use a single dG to present for the experiment condition correctly, there are important assumptions that are often ignored in several DFT paper; the adsorption sites are needed to be identical, and the H-H interactions are almost independent to hydrogen coverage. (J. Phys. Chem. C 2010, 114, 42, 18182-18197). However, the Ir nanoparticle system does not meet the two requirements.*

**Response 5:** We understand the concerns and thank the reviewer for the insightful comments. Although bulk Ir exhibits excellent activity, enhanced HER and OER activities are found with Ir-NSG as ∆*G*_H*_ at Ir(111) is about -0.19 eV and (∆*G*_OOH*_ − ∆*G*_O*_) of Ir_13_@G is 0.88 eV, both are higher than the corresponding energy barriers for Ir_13_@NSG, which are also observed in experiments.

By considering all the possible hydrogen adsorption configurations (H*) of Ir-NSG, we found that the N, S-coordinated Ir site with Δ*G*_H*_ of -0.04 eV shows the best HER activity, as shown in Figure S21 (revised Supplementary Fig. 26). We are sorry that saying “Hydrogen adsorption configurations on atomic Ir active sites” in the original caption clearly caused some confusions. Actually, Ir sites with |Δ*G*_H*_| > 0.30 eV are not highly active for HER. Therefore, we have changed the caption of Figure S21 (revised Supplementary Fig. 26) into “Hydrogen adsorption configurations (H*) on atomic Ir sites and the corresponding Gibbs free energies (Δ*G*_H*_) of Ir_13_@NSG” and added “All the possibilities of H* structures are considered while those with |Δ*G*_H*_| > 0.56 eV are not shown” as details in the revised Supplementary Information.

We agree with the reviewers that the adsorbed hydrogen will easily diffuse to the strong adsorption Ir sites firstly and the hydrogen coverage has been added as a factor to study its effect on the HER activity in our model. As Ir sites of the Ir_13_-NSG model are distinct due to the coordination environment, distinguished HER activities are observed on different Ir sites. According to Δ*G*_H*_ calculated in the original manuscript, Ir_S_* with Ir atom coordinated to one S and one N atom was predicted to be the most active site for HER (Δ*G*_H*_ = -0.04 eV). Thus, we can compare the change of hydrogen adsorption energy on this Ir_S_* site to simply simulate the influence of hydrogen coverage on the HER activity. Two new structure models with different hydrogen coverages are considered, structure A with all iridium sites that show |Δ*G*_H*_| > 0.30 eV adsorbed by hydrogen (Figs. R17a, b) and structure B with all iridium sites except for Ir_S_* adsorbed by hydrogen (Figs. R17d, e). The hydrogen adsorption energy on the Ir_S_* site is -0.06 eV for structure A and -0.08 eV for structure B, indicating that they are still highly active for HER and show small differences in Δ*G*_H*_ compared with that of pure Ir_13_-NSG (Δ*G*_H*_ = -0.04 eV). Besides, the adsorption configuration of each hydrogen atom in structure A or B is almost the same as the corresponding single hydrogen adsorption structure. Therefore, we can conclude that hydrogen coverage and H-H interaction have relatively small effect on the HER activity, and consequently, single hydrogen adsorption energy Δ*G*_H*_ can be used to qualitatively characterize the HER activity of different adsorption sites and address the influence of coordination environment.

**Fig. R17. Geometric structures and hydrogen adsorption structures (H*) of A (all Ir sites with |Δ*G*_H*_| > 0.30 eV are adsorbed by hydrogen atoms) and B (all Ir sites except for Ir_S_* are adsorbed by hydrogen atoms). a,** top view of A. **b,** side view of A. **c,** hydrogen adsorption structure of A. **d,** top view of B. **e,** side view of B. **f,** hydrogen adsorption structure of B.

**Comment 6:** *In Figure S17, the cyclic voltammogram of Ir shows only a peak, which seems that these is only one kind of adsorption site. However, the DFT calculation shows that there are several adsorption sites with different adsorption strengths indicated by different dG. Therefore, I expect that the CV may show more than one peak along the voltages.*

**Response 6:** We thank the reviewer for the helpful comment. On the one hand, as we have stated in the caption of Figure S17 (revised Supplementary Fig. 22), though there is only one H_upd_ peak in the CV curve of Ir-NSG, its broad feature could be interpreted as the existence of a variety of Ir sites, which could be further deconvoluted into multiple peaks to determine the contribution of each site (Zheng, J. et al. *ACS Catal.* 2015, **5,** 4449-4455). On the other hand, in reality, the multiple adsorption sites would be competing based on the Boltzmann distribution of the adsorption energies at different sites. As a result, the most stable ones would be dominant, if not the only one mode, depending upon the adsorption energy differences (and other enthalpic and entropic factors). Due to the ambiguous coordination environment and heterogeneous nature of the sites in Ir-NSG under non-equilibrium thermodynamic conditions in the electrochemical process, it is hardly possible to obtain undoubted peak fitting results at this moment. Therefore, we compared the HBE of Ir-NSG with that of Pt/C as a whole by HUPD measurements qualitatively, and then tested all the possibilities of coordination to identify the most probable active site in Ir-NSG at the atomic level by DFT calculations.

**Comment 7:** *It is not accurate to quantitatively compare dG with HBE obtained from operando X-ray absorption spectroscopy, for example, in line 275. The operando X-ray absorption measures the hydrogen binding energy at potential larger than 0 vs SHE, and this is the measure of the adsorption/desorption of H from/to HClO. However, dG is defined as the free energy between H_2_ and adsorbed H, which is free energy of HER. Also, dG includes the TdS of H_2_ gas (Journal of The Electrochemical Society, 152 (3) J23-J26 (2005))*

**Response 7:** We thank the reviewer for the valuable comment and this is an important point. In fact, the HBEs reported in this work were obtained from CVs instead of *operando* XAFS and we just compared Δ*G* with the obtained HBE qualitatively. To address the reviewer’s concerns, we have added the derivation of the Gibbs free energy for H adsorption (Δ*G*) and HBE (Δ*H*) from H_upd_ using the cyclic voltammogram (CV) method in Note R1. By assuming that H adsorption obeys Langmuir isotherm with considering configurational entropy (Sheng, W. et al. *Nat. Commun.* 2015, **6,** 5848; Karlberg, G. S. et al. *Phys. Rev. Lett.* 2007, **99**, 126101), we can obtain that $\text{Δ}\text{G}\text{ = Δ}\text{H}\text{ }-\text{ }\text{T}\text{Δ}\text{S}_{\text{conf}}\text{ = Δ}\text{H}\text{ }-\text{ }\text{k}_{\text{b}}\text{T}\text{ln}\frac{\text{1-}\text{θ}}{\text{θ}}$, where *k*_b_ is the Boltzmann constant and *θ* is the coverage of hydrogen. At the potential of H_upd_ (*E*_peak_), it can be derived that *θ* is 0.5, and thus Δ*G* = Δ*H* = −*FE*_peak_ (details of the derivation can be found in Note R1). Therefore, a direct and rational link between the Δ*G* and HBE (Δ*H*) determined by experimental CVs appears to have been obtained.

**Comment 8:** *In line 278, it is said that “Ir atom is positively charged”. However, in Figure 22, I see lot of cyan color in Ir, which indicates Ir atoms are negatively charged. (In the description, “The purple and cyan regions refer to positive and negative charge density distributions, respectively.”)*

**Response 8:** We thank the reviewer for pointing out this inconsistency. We are sorry that there is an inadvertent mistake in the caption of Figure S22 (revised Supplementary Fig. 27). Indeed, the purple and cyan regions refer to positive and negative charge density difference distributions, respectively. Thus, positive charge density difference in purple represents electron accumulation while negative charge density difference in cyan represents electron depletion. We have corrected “The purple and cyan regions refer to positive and negative charge density distributions, respectively” as “The purple and cyan regions refer to electron accumulation and depletion, respectively” in the revised Supplementary Information. The conclusion of “Ir atom is positively charged” in the original manuscript is right.

**Comment 9:** *I am supersized that the Tafel slope for Ir and Pt in acidic environment is below 30 mV/dec. It has been discussed before that the mass transfer limitation for high HER active catalysts cannot be avoid using the rotating disk setting. Additionally, and importantly, the impendence from the electrolyte can also affect current measurements on fast HER electrodes even with highly conductive electrolytes such as 0.1 M HClO_4_. (Journal of The Electrochemical Society, 162 (14) F1470-F1481 (2015); Journal of The Electrochemical Society, 157 (11) B1529-B1536 (2010); Journal of The Electrochemical Society, 162 (1) F190-F203 (2015); Energy Environ. Sci., 2014, 7, 2255) Due to these limitations, 30 mV/dec should be always shown. More explanation should be provided.*

**Response 9:** We thank the reviewer for the insightful comments and showing us some excellent background works about Tafel slope. As the reviewer and these works state that the widely accepted Tafel slope of 30 mV decade^-1^ is indeed underestimated due to mass-transport limitation and lack of *i*R correction, there are also many studies showing that a few factors can influence the determination of Tafel slope in rotating disk electrode (RDE) measurement, such as the range of measured current densities and loading amount of catalyst, and etc. For example, Fang et al.’s result shows that the Tafel slope of Pt/C is only 18.6 mV decade^-1^ in a region of small current densities, which increases evidently when the current density is larger than 30 mA cm^-2^ in acidic media (Fang, S. et al. *Nat. Commun.* 2020, **11,** 1029). On the other hand, Lu et al. find that the Tafel slope of Au-Ru-2 NWs is 30.8 and 15.6 mV decade^-1^ at the catalyst loading amount of 0.08 and 0.25 mg cm^-2^, respectively (Lu Q. et al. *Nat. Chem.* 2018, **10,** 456–461). These reports reveal that there are some experimental conditions to mitigate the effect of mass transport limitation and yield low Tafel slope in RDE measurement.

To address the reviewer’s concerns, we measured Tafel slopes of Ir-NSG and Pt/C again and verified the accuracy of our measurement. Firstly, to minimize the possibility of mass transport limitation associated with the reactant to and product from the electrode, solution was stirred during the collection of Tafel data. Secondly, capacitive contributions and additional pseudocapacitive effects were removed by steady-state collection of current density at each applied potential. Solution resistance was also measured prior to data collection and compensated. In addition, sufficient film thickness of catalyst with a loading amount of 0.3 mg cm^-2^ was supplied to ensure a high activity. More details about the experimental measurements are provided in the Experimental Section of the revised manuscript. As shown in Fig. R18, the Tafel slope of Pt/C is still 19.6 mV decade^-1^ in the range of 1 to 10 mA cm^-2^ while increasing to 29.8 mV decade^-1^ when the current density is higher than 10 mA cm^-2^, which is consistent with the finding of Fang et al. (Fang, S. et al. *Nat. Commun.* 2020, **11,** 1029). The higher Tafel slope at large current densities may arise from two reasons. On the one hand, the limitation of reactant transfer becomes more evident at large current densities. On the other hand, at high current densities, excessive H_2_ bubbles can accumulate at the electrode surface due to the transport limitation of product, which leads to a contribution of reverse hydrogen oxidation reaction. All these factors would affect the measured kinetics of HER (Huynh M. et al. *J. Am. Chem. Soc.* 2014, **136,** 6002–6010).

**Fig. R18.** The corresponding Tafel plots for Ir-NSG and commercial Pt/C. All the measurements were operated in Ar-saturated 0.1 M HClO_4_ with *i*R-compensation. Catalyst loading: 0.3 mg cm^-2^. Rotation speed: 1,600 rpm.

**Reviewer comments, second version:**

Reviewer #2 (Remarks to the Author):

The authors have addressed my concerns in the revised manuscript. My last suggestion is to include a more comprehensive comparison in the paper, not just selected some "just so so" data. For example, in some recent studies, RuO2-based and MnO2-based catalysts for acidic OER have been reported. I understand that some parameters (e.g., overpotential) in these studies are even better than those in the present paper. However, I didnot mean that the present paper cannot be published unless all the parameters are the best. A more compresensive comparison would be helpful for the readers in this area.


Reviewer #3 (Remarks to the Author):

The authors did a very good job addressing my concerns. I hence recommend the paper for publications. Minor issues:

Question 1
According to the paper, PRL 99, 126101 (2007) and J. K. Nørskov et al 2005 J. Electrochem. Soc. 152 J23, the entropy change in equation 2 should not only have the configurational term but also the thermodynamic entropy change between the gas and the adsorbed state, which is about +0.2 eV at room temperature. Does this additional entropy change the result for Epeak in Eq 12?
Question 2
Under equation (4), can you make sure the sentence is correct “The Gibbs free energy (∆G4 o) of Rxn (4) is zero at a potential of 0 V under standard conditions” I think that the standard Gibbs free energy of the equation (4) is not zero unless the reaction of equation (4) is under equilibrium condition but not the standard condition.
Question 3
In Equation 1, I believe the equal sign should be a forward arrow sign.

**Author rebuttal, second version:**

**Responses to the remarks of Reviewer #1:**

*The revised manuscript has improved substantially and that publication is recommended. The only remaining suggestion is that more details, on how the authors determined the cell resistance of 26.5, 29.7 and 14.9 ohm for overall water splitting, to be included in the experimental section.*

**Response:** We are grateful to the reviewer for the positive comment and the recommendation for the publication of our work. In our electrochemical tests, all the uncompensated solution resistances were measured via *iR* compensation command on the potentiostat. The measurements were carried out across the test potential of 0 V by applying a step amplitude of 0.05 V to ensure an optimal signal-to-noise ratio and absence of faradic reactions. Besides, the compensation and overshoot levels were set at 100 % and 2 %, respectively. After the desired compensation level was reached or the system was no longer stable, the uncompensated series resistance would be calculated from the measured resistance and the allowed compensation level (He, P. et al. *Anal. Chem.* 1986, **58**, 517-523). The cell resistances of 26.5, 29.7 and 14.9 ohm for overall water splitting were also determined by this method.

Correspondingly, we have added necessary statements in lines 456-459 of the revised manuscript as “Additionally, for all the *i*-*E* curves reported in this study, the solution resistance *R* was measured via *iR* compensation command by applying the test potential, step amplitude, compensation level and overshoot level as 0 V, 0.05 V, 100 % and 2 %, respectively, which was subsequently used to correct the solution Ohmic loss by *E* = *E*_measured_ – *iR*.”.

**Responses to the remarks of Reviewer #2:**

*The authors have addressed my concerns in the revised manuscript. My last suggestion is to include a more comprehensive comparison in the paper, not just selected some "just so so" data. For example, in some recent studies, RuO_2_-based and MnO_2_-based catalysts for acidic OER have been reported. I understand that some parameters (e.g., overpotential) in these studies are even better than those in the present paper. However, I did not mean that the present paper cannot be published unless all the parameters are the best. A more comprehensive comparison would be helpful for the readers in this area.*

**Response:** We appreciate the reviewer for the helpful suggestion and pointing out some nice works about acidic OER catalysts with excellent performance. Following the reviewer’s suggestion, to provide a more comprehensive assessment of state-of-the-art materials, we have thoroughly reviewed recent literatures and complemented CaCu_3_Ru_4_O_12_ (Miao, X. et al. *Nat. Commun.* 2019, **10**, 3809) and Ru_1_-Pt_3_Cu (Yao, Y. et al. *Nat. Catal.* 2019, **2**, 304-313) with low overpotentials for acidic OER as comparisons in Table R1. Consequently, to the best of our knowledge, we have already summarized most recent and impactful works to provide a comprehensive context for our Ir-NSG catalyst in the Supplementary Tables.

**Table R1 and revised Supplementary Table 5.** Comparison of OER activities with various recently reported state-of-the-art noble metal-based catalysts in acidic electrolyte.

| *Catalyst* | *Medium and electrode* | *Scan rate (mV s^-1^)* | *η @ 10 mA cm^-2^ (mV)* | *Tafel slope (mV dec^-1^)* | *Reference* |
| --- | --- | --- | --- | --- | --- |
| Ir-NSG | 0.1 M HClO_4_; glassy carbon | 1 | 265 | 44.2 | This work |
|  |  | 5 | 255 |  |  |
| CaCu_3_Ru_4_O_12_ | 0.5 M H_2_SO_4_; glassy carbon | 5 | 171 | 40 | *Nat. Commun.* **10**, 3809 (2019) |
| Ru_1_-Pt_3_Cu | 0.1 M HClO_4_; glassy carbon | N/A | ca. 220 | N/A | *Nat. Catal.* **2**, 304-313 (2019) |
| Amorphous Ir nanosheets | 0.1 M HClO_4_; glassy carbon | 5 | 255 | 40 | *Nat. Commun.* **10**, 4855 (2019) |
| Ru-N-C | 0.5 M H_2_SO_4_; glassy carbon | 5 | 267 | 52.6 | *Nat. Commun.* **10**, 4849 (2019) |
| IrO_x_/SrIrO_3_ | 0.5 M H_2_SO_4_; SrIrO_3_ film | 10 | 270 | 40 | *Science* **353**, 1011-1014 (2016) |
| 40-IG | 0.5 M H_2_SO_4_; glassy carbon | 5 | 278 | 57 | *Angew. Chem. Int. Ed.* **58**, 12540-12544 (2019) |
| Ru@IrO_x_ | 0.05 M H_2_SO_4_; gold electrode | 5 | 282 | 69.1 | *Chem* **5**, 445-459 (2019) |
| Pr_2_Ir_2_O_7_ | 0.1 M HClO_4_; glassy carbon | 5 | 290 | N/A | *Adv. Mater.* **31**, 1805104 (2019) |
| IrO_x_-Ir | 0.5 M H_2_SO_4_; glassy carbon | 5 | 290 | 43.5 | *Angew. Chem. Int. Ed.* **128**, 752-756 (2016) |
| Dtf-IrOs | 0.1 M HClO_4_; glassy carbon | 10 | ca. 290 | N/A | *Nat. Commun.* **8**, 1449 (2017) |
| Li-IrO_x_ | 0.5 M H_2_SO_4_; glassy carbon | 1 | 300 | 39 | *J. Am. Chem. Soc.* **141**, 3014-3023 (2019) |
| IrCoNi PHNC | 0.1 M HClO_4_; glassy carbon | 5 | 303 | 53.8 | *Adv. Mater.* **29**, 1703798 (2017) |
| IrW/C | 0.1 M HClO_4_; glassy carbon | 5 | ca. 310 | 56.6 | *ACS Cent. Sci.* **4**, 1244-1252 (2018) |
| Ba_2_YIrO_6_ | 0.1 M HClO_4_; gold electrode | 10 | ca. 330 | 67 | *Nat. Commun.* **7**, 12363 (2016) |
| N-HC@G-900 | 0.5 M H_2_SO_4_; glassy carbon | 5 | 350 | 88.1 | *Angew. Chem. Int. Ed.* **130**, 16749-16753 (2018) |
| W_0.57_Ir_0.43_O_3-σ_ | 1 M H_2_SO_4_; glassy carbon | 20 | 370 | 125 | *Energy Environ. Sci.* **10**, 2432-2440 (2017) |
| Sr_2_CoIrO_6_ | 0.1 M HClO_4_; glassy carbon | 10 | ca. 370 | N/A | *Angew. Chem. Int. Ed.* **131**, 4619-4623 (2019) |

Correspondingly, we have added Table R1 as Supplementary Table 5 in the revised Supplementary Information.

**Responses to the remarks of Reviewer #3:**

*The authors did a very good job addressing my concerns. I hence recommend the paper for publications. Minor issues:*

**Response:** We thank the reviewer for the positive comment on our manuscript.

**Comment 1:** *According to the paper, PRL 99, 126101 (2007) and J. K. Nørskov et al. 2005 J. Electrochem. Soc. 152 J23, the entropy change in equation 2 should not only have the configurational term but also the thermodynamic entropy change between the gas and the adsorbed state, which is about +0.2 eV at room temperature. Does this additional entropy change the result for E_peak_ in Eq 12?*

**Response 1:** We are grateful to the reviewer for raising this interesting and meaningful question. In consideration of thermodynamic entropy change for H_2_, Yan et al. has derived the relationship of HBEs and peak potentials in CVs (*E*_peak_) in detail as $\text{∆}\text{H}\text{ = }-{\text{F}\text{E}}_{\text{peak}}\text{ }-\text{ }\frac{\text{1}}{\text{2}}\text{T}S_{H_{2}}^{0}$, where $S_{H_{2}}^{0}$ is the entropy of H_2_ in the gas phase at standard conditions and equal to 130.684 J mol^-1^ K^-1^ (Zheng, J. et al. *ACS Catal.* 2015, **5**, 4449-4455). As all the electrochemical tests were carried out at the same temperature, HBEs are also in a linear correlation with the H_upd_ desorption peak positions as Eq (12) ($\text{E}_{\text{peak}}\text{ = }-\frac{\text{∆}\text{H}}{\text{F}}$). Therefore, this additional entropy change does not affect the feasibility of Eq (12) as a more simple and transparent way to link between the HBEs and experimental CVs qualitatively (Sheng, W. et al. *Nat. Commun.* 2015, **6**, 5848).

**Comment 2:** *Under equation (4), can you make sure the sentence is correct “The Gibbs free energy (*${\text{∆}\text{G}}_{\text{4}}^{\text{o}}$*) of Rxn (4) is zero at a potential of 0 V under standard conditions” I think that the standard Gibbs free energy of the equation (4) is not zero unless the reaction of equation (4) is under equilibrium condition but not the standard condition.*

**Response 2:** We understand this concern and thank the reviewer for the insightful comment. However, a key prerequisite of “at a potential of 0 V” is easily overlooked, which is the equilibrium potential for Rxn (4) ($\text{H}^{\text{+}}\text{ + }\text{e}^{\text{-}}\text{ }\text{⇆}\text{ }\frac{\text{1}}{\text{2}}\text{H}_{\text{2}}$) under standard conditions, and thus this conclusion is indeed also derived under equilibrium condition. Therefore, the sentence of “The Gibbs free energy (${\text{∆}\text{G}}_{\text{4}}^{\text{o}}$) of Rxn (4) is zero at a potential of 0 V under standard conditions” is correct.

**Comment 3:** *In Equation 1, I believe the equal sign should be a forward arrow sign.*

**Response 3:** We thank the reviewer for pointing out this mistake. Accordingly, we have corrected Rxn (1), Rxn (3) and Rxn (4) as $\frac{\text{1}}{\text{2}}\text{H}_{\text{2}}\text{ + * }\text{→}\text{ }\text{H}^{\text{*}}$, $\text{H}^{\text{+}}\text{ +}{\text{ }\text{e}}^{\text{-}}\text{ + * }\text{⇆}\text{ }\text{H}^{\text{*}}$ and $\text{H}^{\text{+}}\text{ + }\text{e}^{\text{-}}\text{ }\text{⇆}\text{ }\frac{\text{1}}{\text{2}}\text{H}_{\text{2}}$ in the revised Supplementary Note 1, respectively

**Reviewer comments, third version:**

**Author rebuttal, third version:**
